# Steric Switching From Photochemical to Thermal N_2_ Splitting: A Computational Analysis of the Isomerization Reaction {(Cp^*^)(Am)Mo}_2_(μ-η^1^:η^1^-N_2_) → {(Cp^*^)(Am)Mo}_2_(μ-N)_2_

**DOI:** 10.3389/fchem.2019.00352

**Published:** 2019-05-16

**Authors:** Vera Krewald

**Affiliations:** Theoretische Chemie, Fachbereich Chemie, TU Darmstadt, Darmstadt, Germany

**Keywords:** nitrogen fixation, molybdenum, density functional theory, isomerization thermodynamics, theoretical UV-vis spectroscopy

## Abstract

A μ-η^1^:η^1^-N_2_-bridged Mo dimer, {(η^5^-C_5_Me_5_)[N(Et)C(Ph)N(Et)]Mo}_2_(μ-N_2_), cleaves dinitrogen thermally resulting in a crystallographically characterized bis-μ-N-bridged dimer, {(η^5^-C_5_Me_5_)[N(Et)C(Ph)N(Et)]Mo}_2_(μ-N)_2_. A structurally related Mo dimer with a bulkier amidinate ligand, ([N(^i^Pr)C(Me)N(^i^Pr)]), is only capable of photochemical dinitrogen activation. These opposing reactivities were rationalized as steric switching between the thermally and photochemically active species. A computational analysis of the geometric and electronic structures of intermediates along the isomerization pathway from Mo_2_(μ-η^1^:η^1^-N_2_) to Mo_2_(μ-η^2^:η^1^-N_2_) and Mo_2_(μ-η^2^:η^2^-N_2_), and finally Mo_2_(μ-N)_2_, is presented here. The extent to which dispersion affects the thermodynamics of the isomers is evaluated, and it is found that dispersion interactions play a significant role in stabilizing the product and making the reaction exergonic. The concept of steric switching is further explored with theoretical models with sterically even less demanding ligands, indicating that systematic ligand modifications could be used to rationally design the N_2_ activation energy landscape. An analysis of electronic excitations in the computed UV-vis spectra of the two complexes shows that a particular type of asymmetric excitations is only present in the photoactive complex.

## Introduction

Catalytic nitrogen fixation with well-defined molecular complexes remains a grand challenge despite decades of research in this field. The research field is driven by the vision that a molecular catalyst capable of catalytically transforming nitrogen atoms from the dinitrogen molecule into ammonia or chemicals of higher economic value would contribute to a more sustainable chemical industry not dependent on fossil resources (Crossland and Tyler, 2010; Broda et al., 2013; Tanabe and Nishibayashi, 2013; Lee et al., 2014; Burford and Fryzuk, 2017; Burford et al., 2017; Connor and Holland, 2017; Creutz and Peters, 2017; Eizawa and Nishibayashi, 2017; Kuriyama and Nishibayashi, 2017; Roux et al., 2017). Industrial ammonia production with the Haber-Bosch process is overall energy efficient, but relies on fossil H_2_ for the steam reforming step (Schlögl, 2008). While a molecular catalyst for NH_3_ production may never be efficient enough to replace the highly optimized Haber-Bosch process, research in this area results in valuable insights into the fundamental principles and electronic structure requirements for N_2_ activation. This in turn may not only be relevant for fertilizer production, but also for alternative fuels that are based on nitrogen instead of carbon (Schlögl, 2010) (Grinberg et al., 2016; Chen et al., 2018). Catalysts that produce ammonia from dinitrogen are based on molybdenum, iron and cobalt (Roux et al., 2017), with many more elements known to be capable of binding N_2_ and activating the strong N-N bond (Burford and Fryzuk, 2017; Klopsch et al., 2017). Strategies toward the development of molecular N_2_ fixation catalysts operating at ambient or close to ambient conditions encompass a better understanding of the nitrogenase cofactor in nature (Lancaster et al., 2011; Spatzal et al., 2011; Sippel and Einsle, 2017), the development of molecular complexes and catalysts that activate or split N_2_ (Dance, 2010; MacLeod and Holland, 2013; MacLeod et al., 2016; Djurdjevic et al., 2017; Eizawa et al., 2017; Sickerman et al., 2017), and the elucidation of the electronic structure of such complexes with computational and spectroscopic studies (Himmel and Reiher, 2006; Studt and Tuczek, 2006; Christian et al., 2007; Huss et al., 2013; Weymuth and Reiher, 2014). Ideally, the complexes will either fully cleave the N_2_ molecule or activate the bond sufficiently that the nitrogen atoms are prepared for subsequent chemical reactions, and be part of a complete catalytic cycle with reasonable turnover numbers and turnover frequencies.

**Figure 1:**
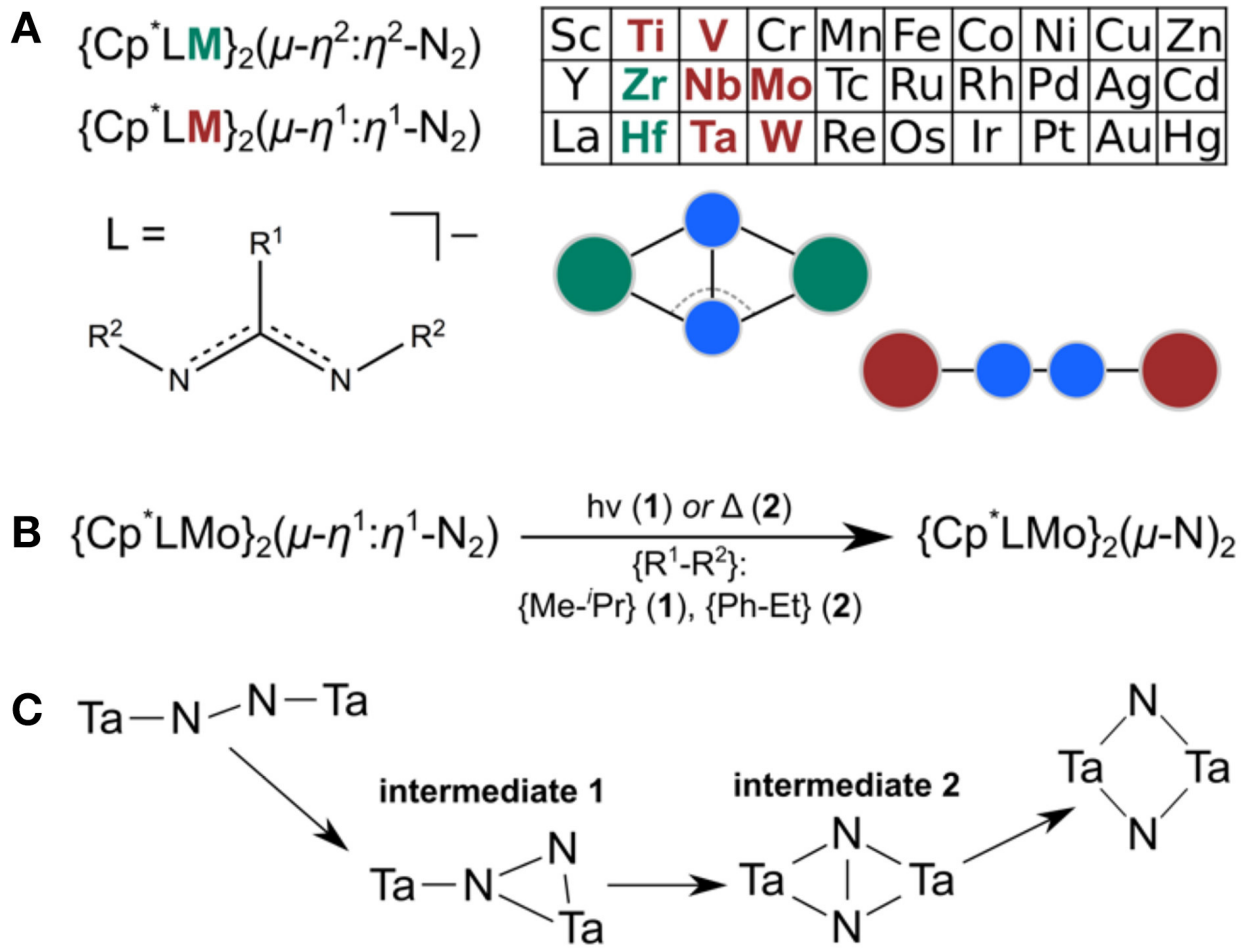
**(A)** Overview of Sita's series of N_2_-bridged complexes; Zr and Hf complexes are non-planar μ-η^2^:η^2^-N_2_-bridged whereas all other known complexes have linear μ-η^1^:η^1^-N_2_-bridged cores. **(B)** Capacity of the molybdenum complexes **1** and **2** for thermal or photochemical dinitrogen activation. **(C)** Isomerization path predicted computationally (Zhang et al., 2011) for the Ta congener using a simplified ligand framework.

Over the past decade, Sita et al. have synthesized and characterized an extensive isostructural series of dinitrogen-bridged dimers which are capable of thermal or photochemical dinitrogen activation (Hirotsu et al., 2007a). The M-(μ-N_2_)-M cores, where M = Ti (Fontaine et al., 2010), V (Keane et al., 2014); Zr (Hirotsu et al., 2007b), Nb (Keane et al., 2014), Mo (Fontaine et al., 2010); Hf (Hirotsu et al., 2007b), Ta (Hirotsu et al., 2007a; Keane et al., 2014), W (Fontaine et al., 2010), are stabilized by a common ligand sphere composed of a Cp^*^ ligand and an amidinate ligand on each metal, see Figure 1A (Yonke et al., 2011a,b; Keane et al., 2013; Farrell et al., 2016; Duman and Sita, 2017). The amidinate ligand can be functionalized at the N-donor atoms or central carbon. Common N-functionalizations are Et and ^i^Pr, and common C-functionalizations are Me, NMe_2_ (i.e., a guanidinate), and Ph. In this paper, the ligand modifications will be denoted as {C-functionalization–N-functionalization}, e.g., {Me–^i^Pr} for the ligand {N(^i^Pr)C(Me)N(^i^Pr)}^−^. The M-(μ-N_2_)-M cores are side-on bridging with a non-planar diamond core for Zr and Hf, and end-on bridging with a linear or near-linear core for Ti, V, Nb, Ta, Mo, and W (Keane et al., 2014).

**Figure 2:**
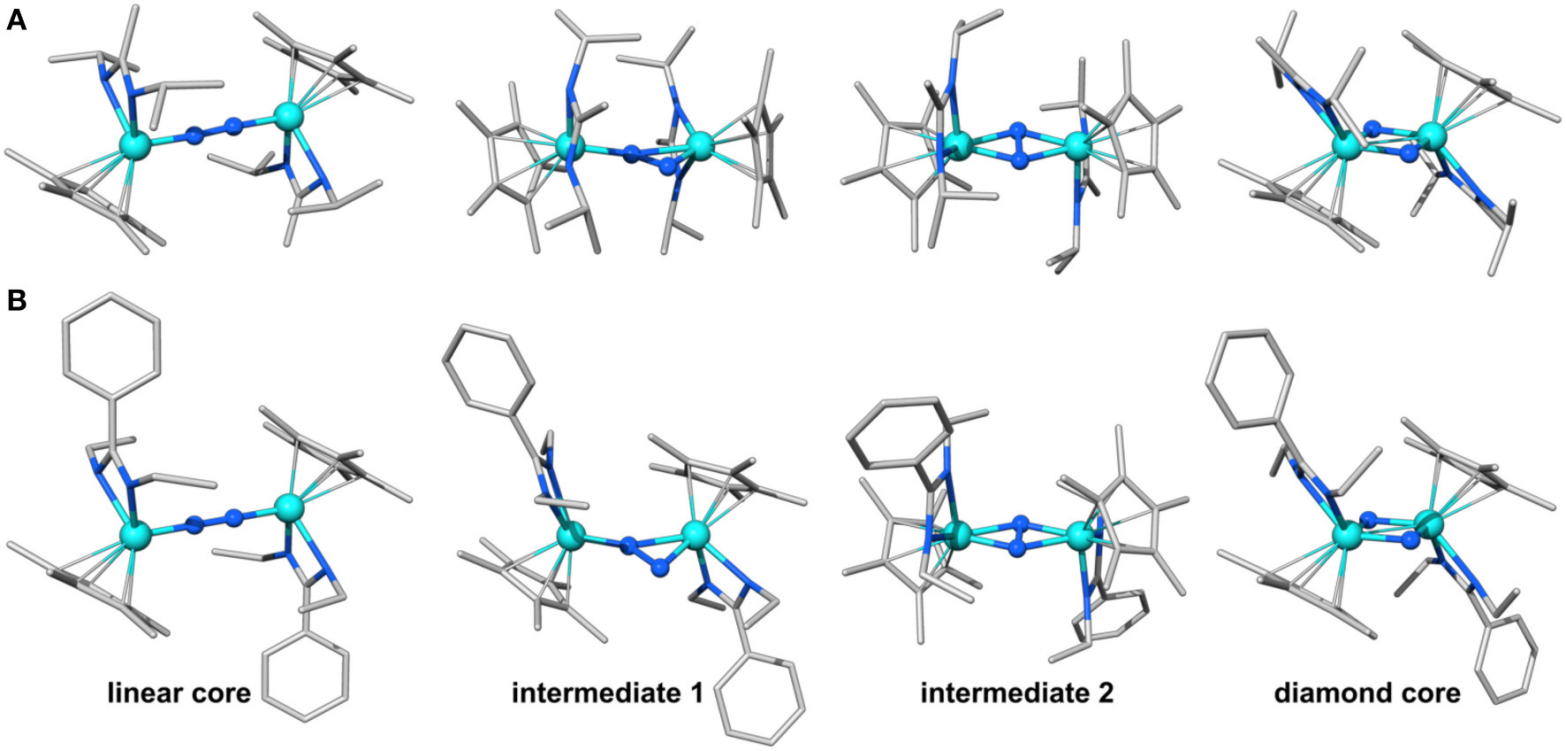
Geometries predicted along the isomerization path of **1 (A)** and **2 (B)**. Color code for atoms is Mo: light blue, N: blue, C: gray, H omitted for clarity.

Most of the complexes in Sita's series are thermally active, i.e., activate or split the N-N bond to yield monomeric metal nitride or dimeric μ-N-bridged complexes initially (Fontaine et al., 2010; Keane et al., 2015; Duman and Sita, 2017). Two complexes based on Mo and W are photoactive, i.e., result in complexes with M_2_(μ-N) and M_2_(μ-N)_2_ cores upon irradiation with a mercury pressure lamp, see Figure 1B (Keane et al., 2015). Photochemical activation of N_2_ (Krewald, 2018) in a well-defined molecular complex was first observed in 2001 (Solari et al., 2001), and has since been shown in several N_2_-bridged dimers based on Mo (Curley et al., 2008; Miyazaki et al., 2014; Keane et al., 2015), W (Keane et al., 2015), Re (Schendzielorz et al., 2019), and Os (Kunkely and Vogler, 2010). Nitrogen photoactivation itself is currently not well-understood with only one time-resolved spectroscopy (Huss et al., 2013) and few computational investigations (Reiher et al., 2004; Krewald and González, 2018) available. The appeal of a light-driven step in nitrogen splitting lies in high spatio-temporal reaction control, high selectivity by depositing a well-defined amount of energy into the catalyst, and potentially the use of sunlight as a free and green source of energy.

For the Mo and W complexes with the {Me–^i^Pr} ligand, Sita et al. showed that they are thermally stable at up to 100°C in hydrocarbon solution, but are sensitive to irradiation from a Rayonet carousel of medium-pressure Hg lamps (Keane et al., 2015). Through photolysis in the presence of a Group 14 alkyl- or aryl-substituted chloride (i.e., R_3_ECl, E = C, Si, Ge), terminal imido products and metal dichloride precursors of the starting complexes are formed. The imido product can react with CO_2_ to form isocyanate derivatives R_3_EN = C = O and a metal oxo complex, which can be transformed into the metal dichloride precursor, thus completing the chemical cycle (Keane et al., 2015). Although the photochemical activation of N_2_ is in principle a desirable reaction, the authors noted that for these particular complexes the reactions were slow and suffered from poor energy efficiency and atom economy. Because the underlying photophysics and photochemistry of nitrogen photofixation are poorly understood in general (Krewald, 2018), Sita et al. subsequently sought to enable the thermal pathway by reducing the steric demands of the {Me–^i^Pr} ligand in the photochemically active Mo and W dimers (Duman et al., 2016).

Steric hindrance as a design feature has been exploited in dinitrogen activation before, e.g., by Cummins et al., who have shown that an undesired mono-μ-N product of their [(N(^t^Bu)(Ar))_3_Mo(μ-N_2_)Mo(N(^t^Bu)(Ar))_3_] complex is avoided (Laplaza and Cummins, 1995; Johnson et al., 1997), unlike in other dinitrogen-splitting complexes (Solari et al., 2001). Similarly, a well-established body of work by Power et al. among others exploits the cumulative effects of dispersion interactions in sterically crowded systems to stabilize otherwise energetically unfavorable complexes (Liptrot et al., 2016; Liptrot and Power, 2017). Seminal work by Grimme has provided the computational basis to explore these effects *in silico* (Grimme, 2004, 2011; Grimme et al., 2011b).

For the Ta, Nb, and Hf congeners an extensive study focused on the link between steric demand and nitrogen activation capacity (Fontaine et al., 2010; Keane et al., 2014). Expanding on these principles, thermal dinitrogen activation in both the Mo and W complexes could indeed be achieved by modifying the amidinate ligand substitution pattern from {Me–^i^Pr} to {Ph–Et} (Duman et al., 2016). Again, the isomerization from a linear to a diamond-shaped core was observed and some of the resulting products were crystallized (Duman et al., 2016). It is not immediately obvious whether the difference in reactivity is mainly due to steric or electronic factors. The set of M_2_(μ-η^1^:η^1^-N_2_) complexes with M = Mo, W are experimentally characterized as having singlet ground states for both the {Me–^i^Pr} and the {Ph–Et} ligands (Fontaine et al., 2010; Keane et al., 2015; Duman et al., 2016). For the M_2_(μ-N)_2_ cores, diamagnetic character is also dominant; however for the molybdenum dimer with {Me–^i^Pr} ligands strong paramagnetic shifting of the ^1^H resonances in the NMR spectra is observed in solution despite SQUID magnetometry data on the solid state sample supporting a closed-shell configuration (Keane et al., 2015). Overall, the crystallographic core geometries and electronic structure properties of the starting complexes are rather similar, warranting a more detailed look at their isomerization reactions.

The isomerization of the Ta dimer from a linear to a diamond-shaped core originally suggested by Fontaine et al. (2010) was explored computationally by Morokuma et al. with a simplified ligand system (Cp instead of Cp^*^; {H–Me} amidinate ligands) (Zhang et al., 2011). According to this study, the linear core, M_2_(μ-η^1^:η^1^-N_2_), transforms into an end-on/side-on bridging core, M_2_(μ-η^2^:η^1^-N_2_), then into a side-on/side-on bridging dimer, M_2_(μ-η^2^:η^2^-N_2_), before fully breaking the N-N bond and forming a diamond-shaped core, M_2_(μ-N)_2_; see Figure 1C (Zhang et al., 2011).

In this contribution, computational analyses based on density functional theory are used to elucidate the ground state geometries and electronic structures of the linear molybdenum complexes {(η^5^-C_5_Me_5_)[N(^*i*^Pr)C(Me)N-(^*i*^Pr)]Mo}_2_(μ-N_2_) (**1**) and {(η^5^-C_5_Me_5_)[N(Et)C(Ph)N-(Et)]Mo}_2_(μ-N_2_) (**2**). The relevant intermediates along the isomerization paths to molybdenum dimers with diamond-shaped cores are identified and characterized to evaluate the idea of steric switching along the isomerization paths. The relevance of dispersion corrections for all intermediates is discussed, and suggestions for complexes with further reduced steric bulk are made. To gain some insight into the photoactivity of the complex with bulkier ligands, the electronic UV-vis absorption spectra are predicted and differences to the spectrum obtained for the thermally active compound are discussed. It is found that a group of transitions with asymmetric MLCT and LMCT character is present in the photoactive, but absent in the thermally active compound. This contribution aims to provide computational insight into the previously unclear ground state electronic structure of the complexes with linear Mo-N-N-Mo cores and identifies possible reasons for the observed differences in reactivity of these structurally similar complexes which may be of relevance for future experimental studies on the complexes' thermodynamic and kinetic properties as well as their photophysical and photochemical processes.

## Materials and Methods

All calculations were performed with the ORCA program package as unrestricted Kohn-Sham calculations (Neese, 2012). Geometries were optimized with the BP86(Perdew, 1986; Becke, 1988) density functional using the resolution of the identity approximation, the def2-TZVP basis set for molybdenum and nitrogen atoms with def2-ECP for molybdenum atoms, the def2-SVP basis for carbon and hydrogen atoms, and the def2/J auxiliary basis (Andrae et al., 1990; Weigend and Ahlrichs, 2005; Weigend, 2006). The grid size was increased to 7 in ORCA nomenclature, and the integration accuracy was set to 7.0. Tight SCF and optimization convergence criteria were chosen. The CPCM implicit solvent model with benzene (ε = 2.28) was used. Grimme's atom-pairwise dispersion correction with Becke-Johnson damping (D3BJ) was used (Grimme et al., 2010, 2011a), except where explicitly excluded as mentioned in the main text.

For the relative energies of spin states, single point energy calculations were performed with the density functionals PBE0 (Adamo and Barone, 1999), TPSSh (Staroverov et al., 2003), B3LYP (Lee et al., 1988; Becke, 1993), M06 (Zhao and Truhlar, 2008), including the chain-of-spheres approximation and the def2/JK basis set (Weigend, 2008; Neese et al., 2009b). To confirm the predicted geometries along the isomerization paths as true minima, the absence of any imaginary frequencies was verified with frequency calculations using the same computational details as for the geometry optimizations, except for the omission of the CPCM solvent model. To obtain a full thermodynamic picture, enthalpies, entropies and Gibbs free enthalpies were taken from these calculations, supplemented with electronic energy calculations with the B3LYP or PBE0 functional using the RIJCOSX approximation with the def2/JK auxiliary basis set.

The UV-vis spectra of the linear complexes were predicted with TD-DFT using various density functionals due to the absence of a calibration study for similar molybdenum dimers. Even though compared with the experimental spectrum of {(η^5^-C_5_Me_5_)[N(^*i*^Pr)C(Me)N-(^*i*^Pr)]Mo}_2_(μ-N_2_), the predicted spectra are shifted to higher energies by ca. 1 eV, the main features are reproduced satisfactorily. Using otherwise identical settings for the electronic structure calculations as for the single point calculations above, the density functionals tested are BP86 (Perdew, 1986; Becke, 1988), TPSS (Tao et al., 2003), TPSSh (Staroverov et al., 2003), B3LYP (Lee et al., 1988; Becke, 1993), PBE0 (Adamo and Barone, 1999), CAM-B3LYP (Yanai et al., 2004), LC-BLYP (Iikura et al., 2001), and ωB97X (Chai and Head-Gordon, 2008). The Tamm-Dancoff approximation(Hirata and Head-Gordon, 1999) was used and 100 roots were calculated (Neese and Olbrich, 2002). The solvent modeled was methyl cyclohexane (ε = 2.071). All line spectra are generated with the ORCA utility program *orca_mapspc* with spectral broadening of 2,500 cm^−1^.

## Results

### Geometries and Electronic Structures of Molybdenum Dimers With Linear and Diamond-Shaped Cores

The starting complexes are {(η^5^-C_5_Me_5_)[N(^*i*^Pr)C(Me)N-(^*i*^Pr)]Mo}_2_(μ-N_2_), **1**_lin_, which is photoactive, and {(η^5^-C_5_Me_5_)[N(Et)C(Ph)N-(Et)]Mo}_2_(μ-N_2_), **2**_lin_, which is thermally active. They may be formally viewed as two Mo(II), d^4^, with a neutral N_2_ bridge, or as two Mo(IV), d^2^, with a ${\text{N}}_{2}^{4-}$ bridge (Fontaine et al., 2010). A crystal structure is only available for **2**_lin_, where the N-N bond length of 1.288 Å corresponds approximately to a dinitrogen double bond (Holland, 2010). As noted by Fontaine et al., the true electronic configuration probably lies somewhere in-between the oxidation states that can be assigned formally (Fontaine et al., 2010).

The crystal structures of the product complexes {(η^5^-C_5_Me_5_)[N(^*i*^Pr)C(Me)N-(^*i*^Pr)]Mo}_2_(μ-N)_2_, **1**_dia_, and {(η^5^-C_5_Me_5_)[N(Et)C(Ph)N-(Et)]Mo}_2_(μ-N)_2_, **2**_dia_, show diamond-shaped Mo_2_N_2_ cores. Formally, the molybdenum ions are now oxidized to Mo(V), d^1^, implying that in principle a singlet and a triplet ground state are accessible. In the solid state, magnetic data obtained from SQUID magnetometry indicate a closed-shell electronic structure for **1**_dia_. Notably, the ^1^H NMR resonances of solutions of **1**_dia_ are subject to paramagnetic shifting whereas for **2**_dia_ no indication for open-shell character is found (Keane et al., 2015; Duman et al., 2016). The N-N distances are >2.5 Å and thus no residual bonding interaction is to be expected. The crystallographic Mo-Mo distances on the other hand are slightly shorter than twice Pauling's covalent bond radius for Mo (1.371 Å). At 2.676 and 2.648 Å, respectively, they are in the range of distances expected for a Mo-Mo single or double bond (Pauling and Kamb, 1986; Shin and Parkin, 1998; Neary and Parkin, 2017). Both diamond cores are to some extent asymmetric: the molybdenum ions in **1**_dia_ have different bond lengths with the bridging nitrogen atoms (Mo^1^-N^1, 2^: 1.850 Å, Mo^2^-N^1, 2^: 1.964 Å), whereas each molybdenum in **2**_dia_ has two different bond lengths with the nitrogen bridges (1.892, 1.927 Å) (Keane et al., 2015; Duman et al., 2016). From a purely structural point of view, the two complexes have strikingly similar geometries that do not indicate any major influence of the ligand sphere on the geometry of the Mo_2_N_2_ core.

**Figure 3:**
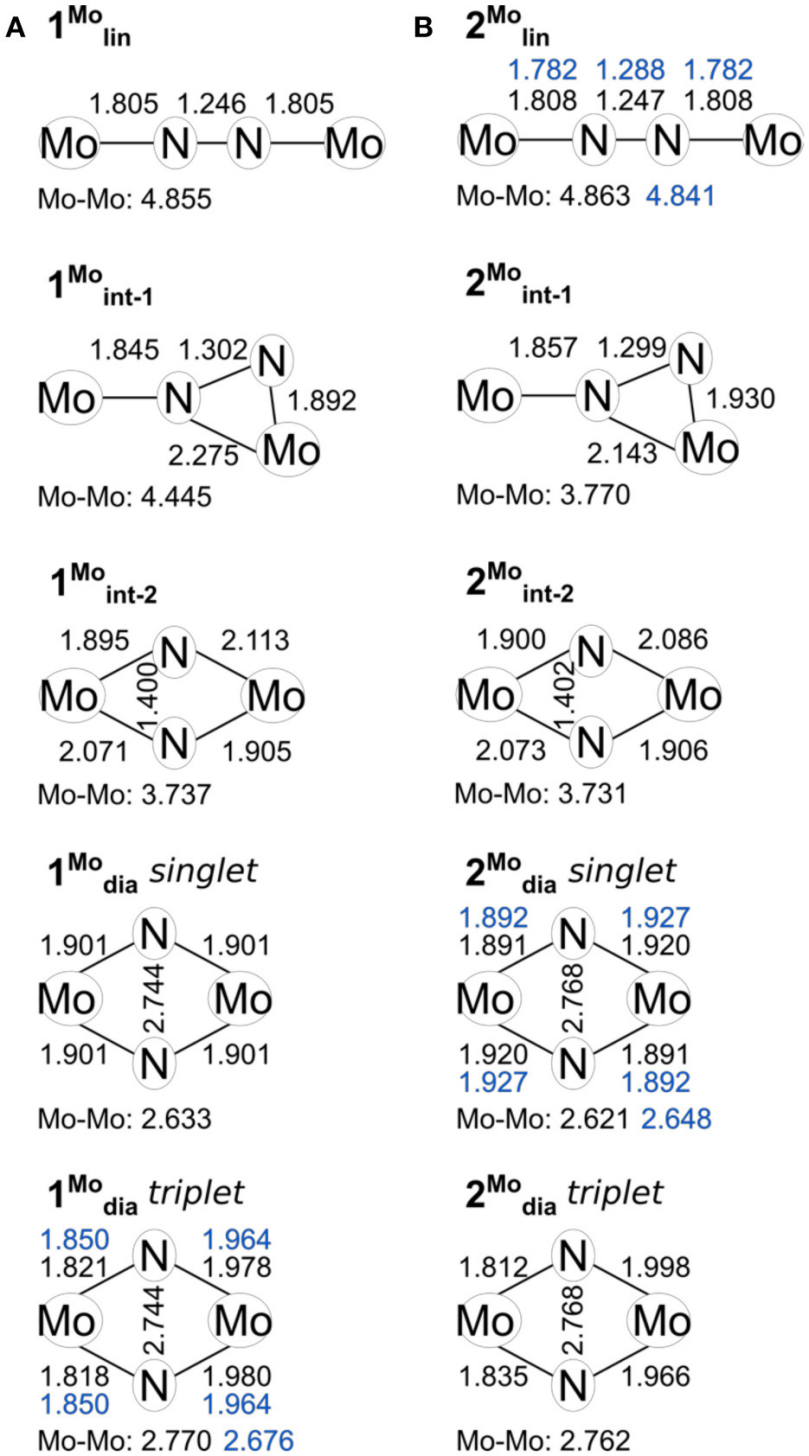
Key geometric parameters computed for the isomers of **1 (A)** and **2 (B)**, including the core geometries of **1**/**2**_dia_ in their triplet states. Values found in crystal structures where available are given in blue.

The structural parameters obtained for **1**_lin_ from a geometry relaxation are in good agreement with those of related complexes such as **2**_lin_ (Figure 3). In the absence of a crystal structure for **1**_lin_, no direct comparison to experimental data is possible. The geometry optimization of **2**_lin_ results in excellent agreement with the available crystal structure. The N-N distance is found to be 1.247 Å (exp.: 1.288 Å) and the Mo-N bond lengths are predicted to within 0.03 Å. In both linear molybdenum dimers, the Mayer bond orders indicate a N-N double bond (1.64) and bonds that are in-between single and double bonds for the M-N interaction (1.39, see Table 1).

**Table 1:** Computed Mayer bond orders along the isomerization coordinate of **1** and **2**, including for the singlet (s) and triplet (t) configurations of **1**/**2**_dia_.

**Cpd**.	**Mo-Mo**	**Mo-N**	**Mo-N**	**Mo-N**	**Mo-N**	**N-N**
1_lin_	0.32	1.39	0.12	1.39	0.12	1.60
1_int−1_	0.26	1.31	0.14	1.23	0.36	1.24
1_int−2_	0.35	1.14	0.69	1.12	0.61	0.88
1_dia_ (s)	0.70	1.23	1.23	1.23	1.23	<0.1
1_dia_ (t)	0.43	1.56	1.53	1.00	1.02	<0.1
2_lin_	0.31	1.39	0.11	1.39	0.11	1.64
2_int−1_	0.27	1.19	0.17	1.15	0.51	1.14
2_int−2_	0.33	1.13	0.68	1.09	0.64	0.89
2_dia_ (s)	0.73	1.13	1.36	1.36	1.13	<0.1
2_dia_ (t)	0.42	1.47	1.66	1.11	0.94	<0.1

For the product **1**_dia_, the geometry relaxation with a singlet electronic configuration yields a symmetric diamond core with a Mo-Mo distance of 2.633 Å, a N-N distance of 2.744 Å, and Mo-N bond lengths of 1.901 Å. This structure is clearly at odds with the crystallographically determined asymmetry of the core, in which the two molybdenum ions have different bond lengths with the bridging nitrogen atoms. Therefore, a separate geometry relaxation was performed using the triplet state of **1**_dia_. This resulted in a geometry that contains one Mo center with shorter Mo-N bond lengths (1.818, 1.821 Å) and one Mo center with longer Mo-N bond lengths (1.978, 1.980 Å). While overall an improvement in relation to the experimentally found structure, the Mo-Mo distance is now somewhat overestimated (calc: 2.770 Å, exp: 2.676 Å, Figure 3A).

The singlet and triplet geometries of **1**_dia_ are energetically separated by 7.4 kcal/mol at the level of theory of the geometry optimization, which would indicate that the higher-lying triplet state is not significantly populated. Although without careful calibration it is uncertain whether any given density functional yields accurate spin state energetics, hybrid functionals may be considered as somewhat more suitable in this case. Single point energy calculations show a consistently smaller separation between the singlet and triplet state geometries, with the singlet state remaining lower in energy (PBE0: 1.9 kcal/mol, TPSSh: 4.1 kcal/mol, B3LYP: 2.0 kcal/mol, M06: 3.9 kcal/mol). It must be stressed, however, that a reliable estimate of the energy separation could only be achieved with highly accurate methods such as DLPNO-CCSD(T) in combination with a large basis set (Neese et al., 2009a; Paulechka and Kazakov, 2017; Saitow et al., 2017). The Mayer bond orders of the singlet state are reflective of the symmetric core (Mo-Mo: 0.70, all Mo-N: 1.23). The triplet state features a significantly reduced Mo-Mo bond strength (0.43) and asymmetry in the Mo-N bond orders (1.53, 1.56; 1.02, 1.00). The Mulliken spin populations of the triplet state are 0.50 and 1.43, which is indicative of a degree of spin delocalization between a Mo(VI) center and a Mo(IV) center, respectively.

For the product **2**_dia_, a geometry optimization with an electronic singlet state configuration reproduces the crystal structure parameters very well, see Figure 3B. The distortion of the diamond core is accompanied by different bond distances to the amidinate-N donor atoms (2.200, 2.220 Å), all of which are longer than in the starting complex **2**_lin_ (2.130–2.137 Å). The Mayer bond orders indicate a single bond between the two metals (0.73) and strong Mo-N interactions that mirror the asymmetry of the core (1.13, 1.36 for each Mo).

Naturally, the question arises whether a triplet state can also be found for **2**_dia_, and what its energetic separation from the singlet state would be. There is no experimental indication for any open-shell character of this complex. Nevertheless, the geometric and electronic structure resulting from the triplet state relaxation of **2**_dia_ is surprisingly similar to that of **1**_dia_ in its triplet state. The Mo-Mo distance is elongated to 2.762 Å, and asymmetric Mo centers with respect to their interaction with the bridging nitrogen atoms are found (1.812, 1.835, 1.998, 1.966 Å). Similarly, the Mo-Mo Mayer bond order is reduced to 0.42, and the Mo center with short M-N bonds has higher bond orders (1.47, 1.66) than that with longer M-N bonds (1.11, 0.94). The Mulliken spin populations (0.46, 1.38) match those found for the triplet state of **1**_dia_. The relative total energies of the singlet and triplet state of **2**_dia_ are increased compared with **1**_dia_ with the singlet state again remaining the more stable one (BP86: 8.3 kcal/mol, PBE0: 4.0 kcal/mol, TPSSh: 5.5 kcal/mol, B3LYP: 4.01 kcal/mol, M06: 6.15 kcal/mol). Although a clear differentiation between the relative abundance of singlet and triplet state for the diamond cores in **1** and **2** will require further experimental information coupled to high accuracy computations, the present results corroborate that a triplet state may be more readily accessible in **1**_dia_ than in **2**_dia_. The small energy differences between singlet and triplet states are furthermore in agreement with the experimental observation that changes in the environment, e.g., crystal structure vs. solution phase, may well be sufficient to introduce a spectroscopically significant amount of the higher spin state in **1**_dia_ (Keane et al., 2015).

### Isomerization Paths

The structures of the molybdenum dimers with the same multiplicity are strikingly similar, regardless of the ligand sphere. Merely from the inspection of their geometries and electronic structures it is therefore not obvious why **1**_lin_ requires activation with light to achieve the transformation to the diamond-shaped core whereas **2**_lin_ is capable of thermal isomerization. The next step toward elucidating this difference in reactivity was therefore to identify intermediates along the isomerization pathway. This may also lead to a better understanding of the atomic-level realization of “steric switching” (Duman et al., 2016) from the photochemical to the thermal process. Morokuma et al. evaluated a four-step isomerization path for the Ta complexes with a simplified ligand sphere ({H–Me}, i.e., R^1^: H, R^2^: Me), as sketched in Figure 1C (Zhang et al., 2011). It involves a change in dinitrogen coordination from Ta_2_(μ-η^1^:η^1^-N_2_) to Ta_2_(μ-η^2^:η^1^-N_2_) in intermediate 1 and subsequently to Ta_2_(μ-η^2^:η^2^-N_2_) in intermediate 2 with a residual N-N bond, before fully breaking the N-N interaction in Ta_2_(μ-N)_2_. Given that the geometries and reactivities of the complexes in the Sita series are largely similar, these intermediates represent a reasonable starting point for an investigation of the isomerization paths of the Mo dimers.

Indeed, the analogous intermediates were identified for both dimers (Figures 2, 3). Overall, their respective geometries are of striking similarity. Upon formation of **1**/**2**_int−1_ with a μ-η^2^:η^1^-N_2_ bridge, the N-N distance increases to ca. 1.30 Å in both complexes, concomitant with a significant reduction in N-N bond order (Table 1). The Mo-N bond lengths of the η^1^-coordinated Mo are 1.845 Å (**1**_int−1_) and 1.857 Å (**2**_int−1_), thus increased by only ca. 0.05 Å compared to the linear precursors. As expected, the η^2^-coordinated Mo-N interactions are significantly longer than the previous η^1^-distances at now 1.892 Å (**1**_int−1_) and 1.930 Å (**2**_int−1_) for the ‘short' and 2.275 Å (**1**_int−1_) and 2.143 Å (**2**_int−1_) for the “long” interactions. Despite the latter distances being relatively long, the bond orders support a very weak bond (Table 1). In the second intermediates, **1**/**2**_int−2_ with a μ-η^2^:η^2^-N_2_ unit, the nitrogen-nitrogen distances are ca. 1.40 Å and the Mayer bond orders indicate that a single N-N bond is still present (0.88, 0.89). Both Mo_2_N_2_ cores show asymmetry in the Mo-μ-N bond lengths: similar to the singlet form of **2**_dia_, each Mo ion has a short (ca. 1.9 Å) and a long (ca. 2.1 Å) separation from the bridges. The bond orders show a discrepancy in the interaction strength of less than half a single bond.

**Figure 4:**
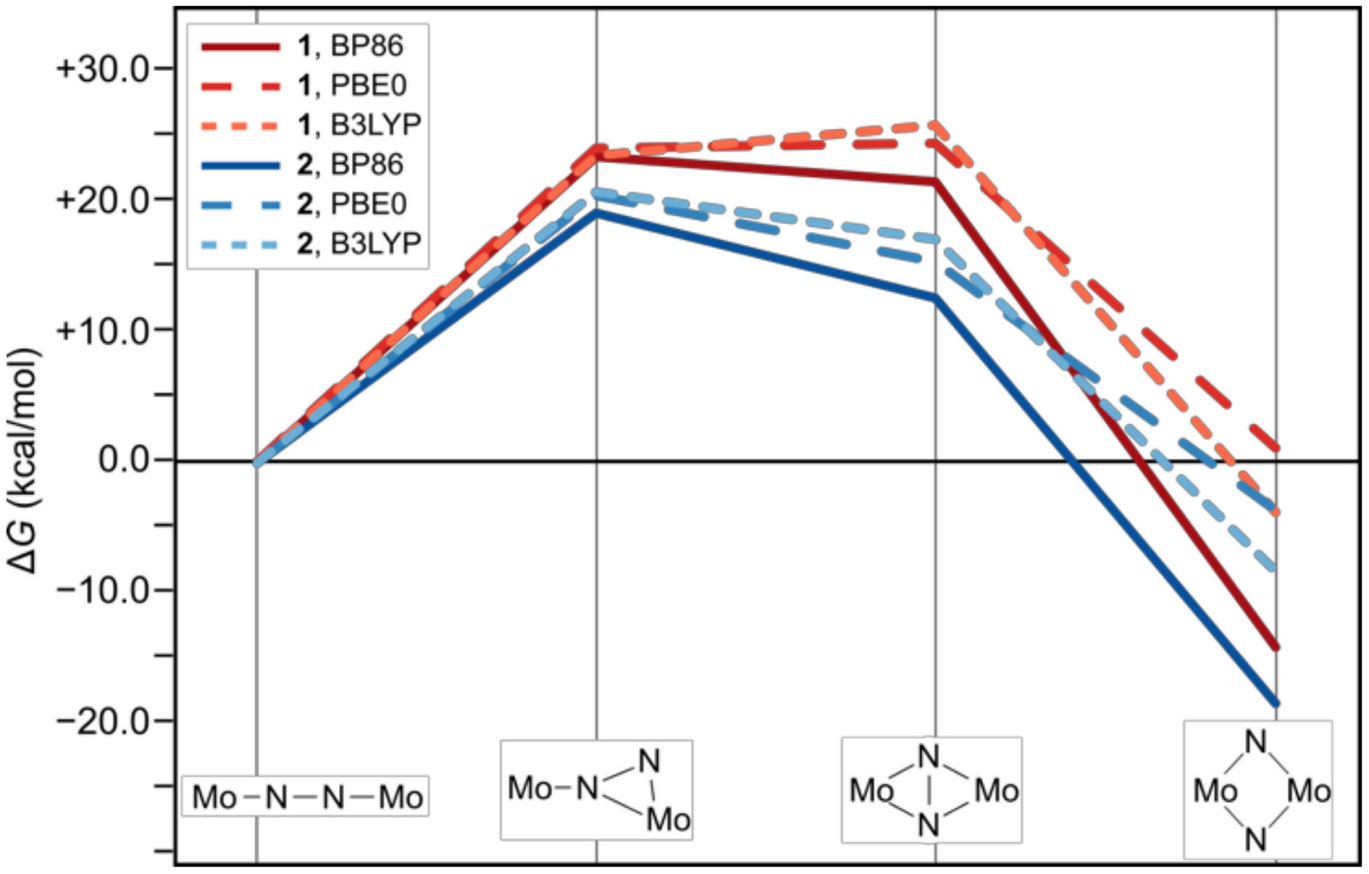
Thermodynamic profile of the isomerization paths for **1** (red) and **2** (blue). Data points for which the electronic structures are calculated with PBE0 and B3LYP are long-dashed and short-dashed, respectively.

The thermodynamic profiles of the isomerization paths in Figure 4 show that the formation of all intermediates is endergonic. At the level of theory used for optimizing the geometries and obtaining the Hessians, the first intermediates are predicted at Δ*G* values of 23.5 kcal/mol (**1**_int−1_) and 19.1 kcal/mol (**2**_int−1_). The formation of the second intermediates is only slightly less endergonic for **1**_int−2_ at 21.7 kcal/mol, but significantly less so for **2**_int−2_ at 12.5 kcal/mol. Finally, the driving force for the overall isomerization appears to be the formation of the dimers with diamond-shaped cores at −14.3 kcal/mol for **1**_dia_ and −18.7 kcal/mol for **2**_dia_ according to the Δ*G* values predicted at this level of theory. To verify whether the electronic structure description would have a significant influence on the overall profiles, single point energy calculations with the two hybrid functionals PBE0 and B3LYP were carried out. The resulting reaction profiles, using the thermodynamic data calculated with the BP86 density functional, shows that the change in Δ*G* for **1**/**2**_int−1_ is <2 kcal/mol while **1**/**2**_int−2_ are predicted to be 3–5 kcal/mol less stable. More importantly, however, the product with a diamond-shaped Mo_2_(μ-N)_2_ core for **1** is calculated to be significantly less exergonic (−3.9 kcal/mol with B3LYP) or even endergonic (+1.0 kcal/mol for PBE0). In contrast, while the product of the isomerization reaction for **2** is also less stabilized with hybrid functionals it is clearly still exergonic at −8.5 kcal/mol with B3LYP or −3.8 kcal/mol with PBE0.

The reaction profiles thus show that isomerization can in principle occur in both complexes, albeit at higher cost for intermediate formation and a significantly lower driving force in complex **1** compared with complex **2**. Comparison with the experimental kinetic data would require the identification of transition states along the isomerization path, which is beyond the scope of this work. The core geometries are strikingly similar and do not readily explain the thermodynamic differences, therefore any attempt to analyze and rationalize the underlying reasons for the experimentally observed “steric switching” must inspect the interactions between the two halves of the ligand sphere. Indeed, an interesting point was noted when analyzing the contributions from the dispersion correction.

### Influence of Dispersion on the Isomerization Path Energetics

**Figure 5:**
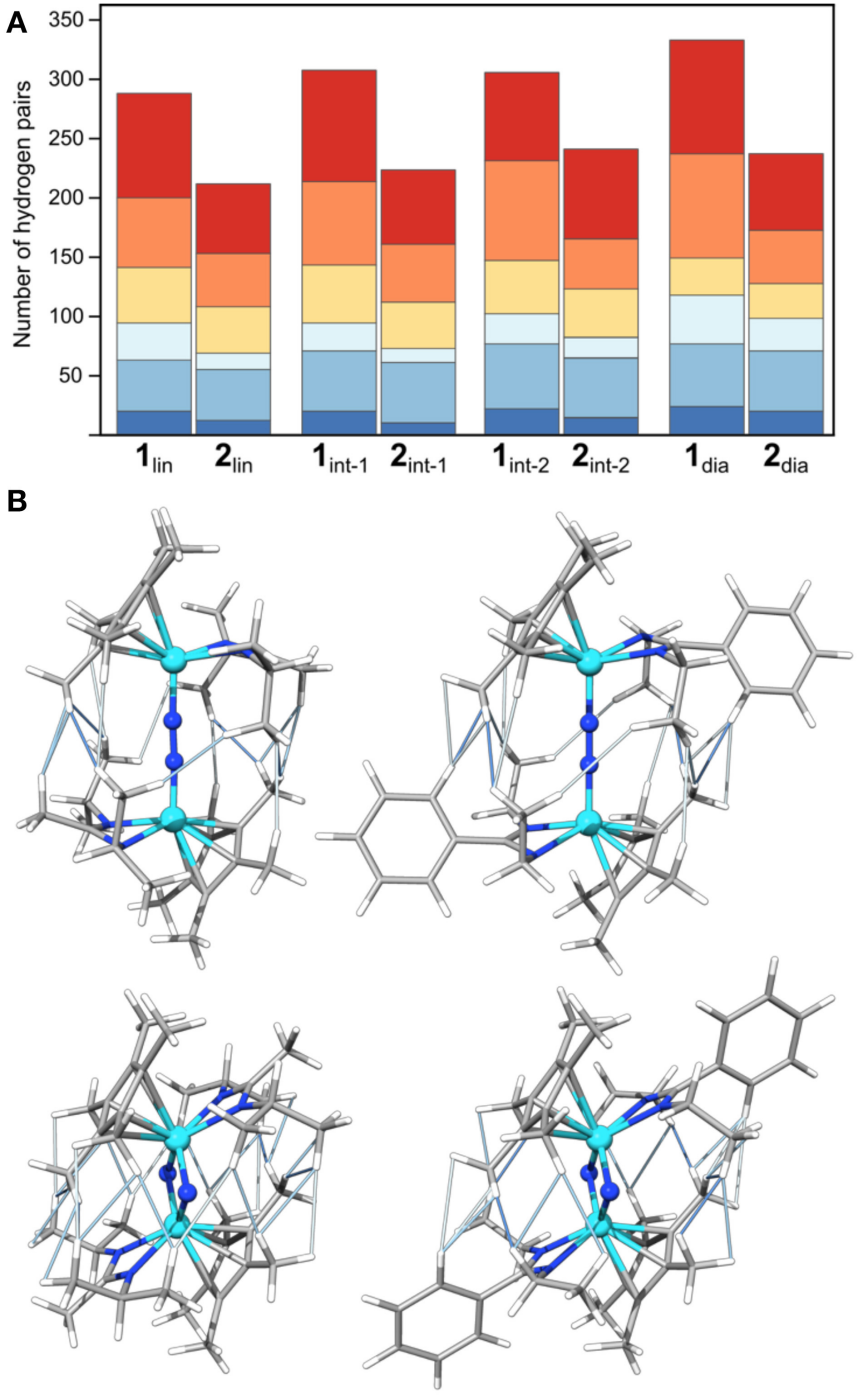
Visualization of the number of hydrogen pairs along the isomerization pathway of **1** and **2, (A)** in terms of absolute numbers in bins of 1.8–2.2 Å (dark blue), 2.2–2.6 Å (middle blue), 2.6–3.0 Å (light blue), 3.0–3.4 Å (yellow), 3.4–3.8 Å (orange), 3.8–4.2 Å (red), and **(B)** as a representation of the actual geometric interactions in **1**_lin_ (top left), **1**_dia_ (top right), **2**_lin_ (bottom left) and **2**_dia_ (bottom right); H-H interactions are shown as thin lines in dark blue (1.8–2.2 Å), middle blue (2.2–2.6 Å), and light blue (2.6–3.0 Å). Color code for atoms is Mo: light blue N: blue, C: gray, H light gray.

**Figure 6:**
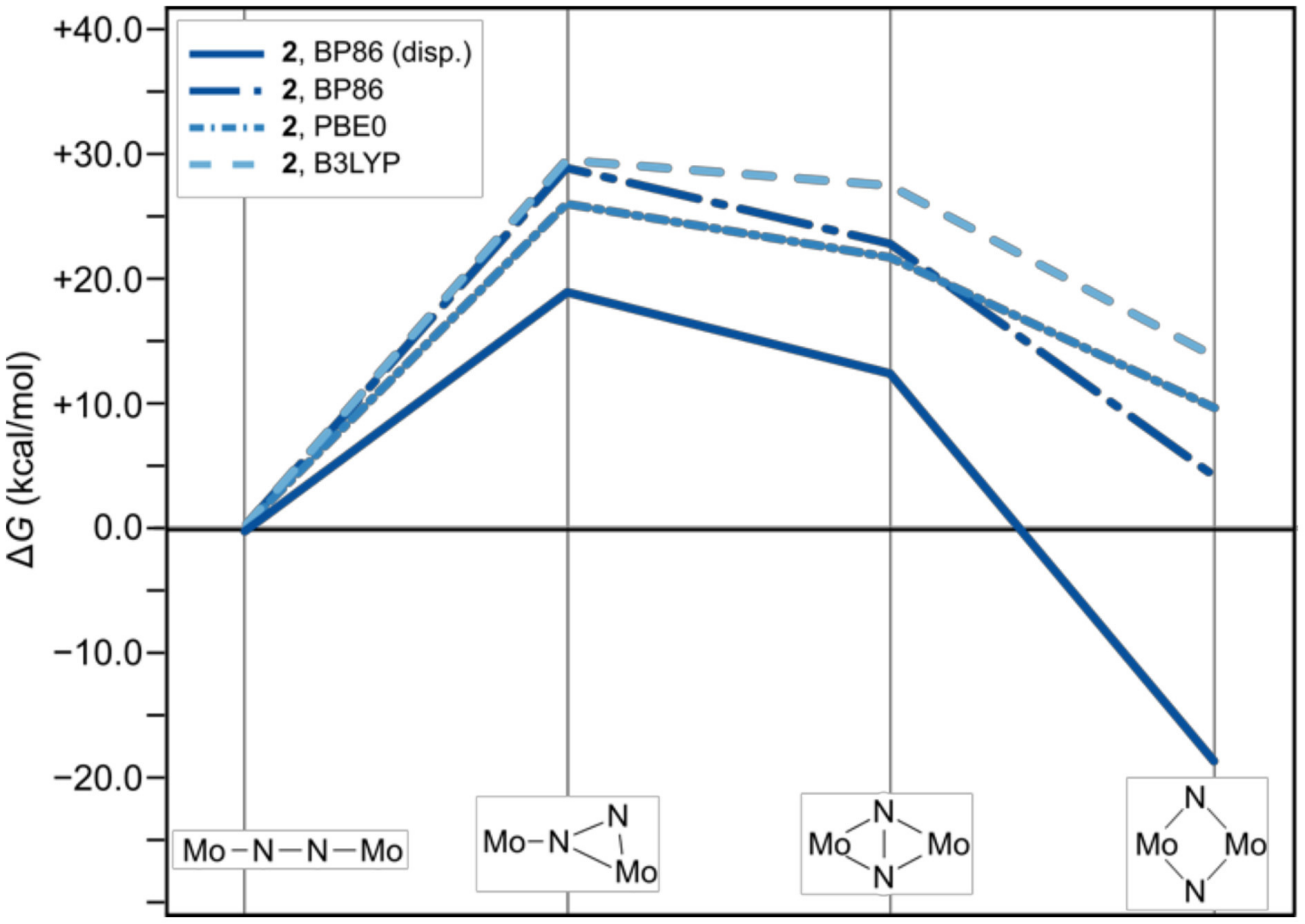
Effect of omitting dispersion corrections on the thermodynamic profile of the isomerization paths of **2**. The thermodynamic profile including dispersion is shown as a solid line for reference; data points for which the electronic structures are calculated with BP are long-dashed-dotted (dark blue), with PBE0 are short-dash-dotted (mid-blue), and with B3LYP short-dashed (light blue).

Initially, the number and distance of pairs of hydrogen atoms will serve as a measure for steric interactions in the intermediates. This provides an intuitive picture of the steric clashes while at the same time facilitating a quantitative comparison along the isomerization paths. As reference values, hydrogen atoms of the same methyl group are usually <1.8 Å apart; distances between hydrogen atoms attached to adjacent carbon atoms in phenyl and ethyl groups are ~2.5 Å. Figure 5 shows the number of H-H distances for the species along the isomerization coordinate in bins of 0.4 Å width between 1.8 and 4.2 Å. The precise numbers, average values and combined lengths for the H-H pairs are given in the SI. Generally, complex **1** has more H-H pairs than complex **2**, in line with the chemical expectations for the “bulky” {Me-^i^Pr} and “sterically reduced” {Ph-Et} ligand systems. For both complexes the number of H-H contacts increases during the overall isomerization process from **1**/**2**_lin_ to **1**/**2**_dia_ (286 to 330 for **1**, 210 to 236 for **2**). For **1**, the increase stems largely from longer distances (+36 for 3.4–4.2 Å, orange and red in Figure 5, cf. +6 for **2** in this range), whereas for **2** the number of shorter distances increases most (+16 for 1.8–2.6 Å, dark and medium blue in Figure 5; cf. +15 for **1** in this range). The inter-hydrogen distances between ligands coordinated at different metals in the bins 1.8–2.2 Å (dark blue), 2.2–2.6 Å (middle blue), and 2.6–3.0 Å (light blue) are visualized in Figure 6 for the linear and diamond-shaped complexes of **1** and **2**. In terms of absolute numbers, these fall in the range of 2.03–2.73 Å for **1**_lin_ and 2.15–2.98 Å for **2**_lin_; 2.03–2.78 Å for **1**_dia_ and 1.96–2.84 Å for **2**_dia_.

The effect of these interactions on the relative energetics are expected to be dominated by two factors: destabilization due to closer nuclei positions, and stabilization due to dispersion interactions. Since dispersion interactions are always attractive (Grimme, 2011), and a larger number of interhydrogen contacts is built up along the reaction coordinate, it is clear that dispersion interactions should stabilize the products to a significant extent. In terms of single point energies, dispersion corrections account for a stabilization of ca. −23 kcal/mol for **1**/**2**_dia_ relative to **1**/**2**_lin_, i.e. the energy gain in both complexes is practically identical. The stabilization of intermediates **1**/**2**_int−1_ and **1**/**2**_int−2_ due to dispersion is also almost identical in the two complexes and <10 kcal/mol relative to the linear starting compounds. Therefore, despite an overall larger number of H-H pairs <4.2 Å apart, complex **1** benefits only as much as complex **2** from stabilization through dispersion, implying that each H-H pair in **1** has a smaller effect. The absolute dispersion corrections in all intermediates of **1** are ca. 5 kcal/mol less than for those of **2** (see SI for details).

The effect of omitting dispersion effects can be tested *in silico* by removing the dispersion corrections in the geometry optimizations, which is presented exemplarily for complex **2** starting from the fully optimized structures that included dispersion corrections. As an aside, it is noted that upon removal of dispersion corrections the geometry of intermediate **1**_int−1_ could not be optimized despite several attempts; instead the molecule relaxed to a linear Mo–(μ-η^1^:η^1^-N_2_)–Mo core. Likewise the optimization of **1**_dia_ without dispersion corrections resulted in a frequency analysis with significant imaginary entries that could not be removed, indicating that the structures obtained are not a proper minima on the potential energy surface. For complex **2** with its starting complex, intermediates and product, the changes in geometry range from barely visible by eye inspection to subtle in many of the isomers, while in some the phenyl group rotates (see SI for Cartesian coordinates of all geometries). Comparing the total number of H contacts in the structures optimized with and without dispersion corrections shows that **2** loses 18 pairs in its linear form and 12 pairs in its diamond form (see Supporting Information).

The thermodynamic profiles for all forms of **2** optimized without dispersion corrections show that the isomerization path is significantly destabilized. Dispersion interactions thus have a more pronounced stabilizing effect on the dimer with a diamond-shaped core compared to the starting complex with a linear core, consistent with the increase in H-H pairs with distances smaller than 4.2 Å. The isomerization product of **2** has a Δ*G* value of 4.1 kcal/mol using the BP86 electronic structure description. An even more distinct destabilization is predicted with the PBE0 (**2**_dia_: 9.6 kcal/mol) and B3LYP (**2**_dia_: 13.7 kcal/mol) density functionals.

### *In silico* Modifications

With the above information on the isomerization paths for **1** and **2** at hand, *in silico* modifications of the amidinate ligand are carried out. The leading question is whether any further reduction of the steric demands of the ligands would lead to lower energy intermediates. The ligand systems considered are {Me-Et} (**3**) and {Ph-H} (**4**). While a substitution pattern of {H-H} would obviously represent the sterically least demanding amidinate ligand, and thus serve as a sort of base line, geometry optimizations with this ligand were unsuccessful for all steps of the isomerization except intermediate 2. All energetic minima identified showed negative frequencies of significant magnitude (>>100 cm^−1^) in their Hessians, indicating that these hypothetical structures would not be stable.

The starting compound **3** has a linear core with key geometric parameters that are almost identical to that of the original compounds **1** and **2**. In contrast, the optimized geometry of **4** has a “pre-bent” core with Mo-N-N angles of 163.5 and 163.3°, compared with 177.2–179.1° for the other three complexes discussed here. None of the other key geometric parameters show a great variation between the four substitution patterns, see Table 2. Likewise, the degree of N-N bond activation as judged by bond length and Mayer bond order is basically unchanged by different amidinate substitution patterns. The only exception to this is intermediate 1 where the bond order is 1.05 for **3** and 1.07 for **4** vs. 1.24 in **1** and 1.14 in **2**, indicating that a lower steric demand or fewer attractive dispersion interactions allow for a greater weakening of the N-N bond.

**Table 2:** Key interatomic distances in Å of the Mo_2_N_2_ cores in the hypothetical compounds **3** and **4**.

**Cpd**.	**Mo-Mo**	**Mo-N**	**Mo-N**	**Mo-N**	**Mo-N**	**N-N**
3_lin_	4.86	1.81	3.05	1.81	3.05	1.25
3_int−1_	3.66	1.85	3.08	1.93	2.12	1.31
3_int−2_	3.72	1.89	2.08	1.89	2.08	1.41
3_dia_ (s)	2.62	1.91	1.90	1.91	1.90	2.77
4_lin_	4.83	1.81	3.03	1.81	3.03	1.24
4_int−1_	3.55	1.84	3.10	1.94	2.16	1.29
4_int−2_	3.72	1.90	2.06	1.90	2.06	1.40
4_dia_ (s)	2.63	2.03	1.85	1.80	1.95	2.72

**Figure 7:**
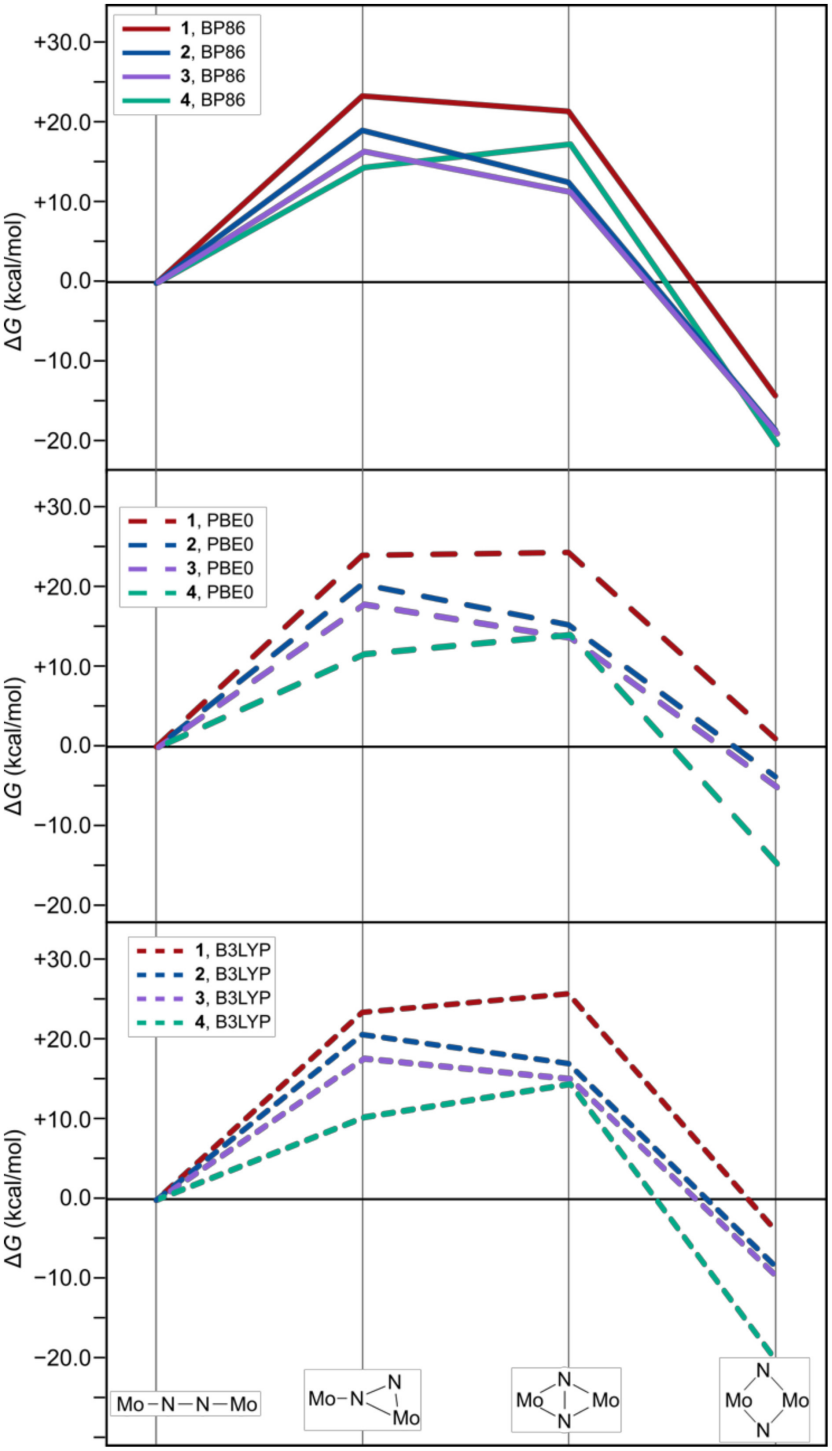
Thermodynamic profiles for **1**, **2**, and {Me-Et} (**3**) and {Ph-H} (**4**) as two systems with further reduced steric bulk. The top, middle and bottom panel show data for electronic structures calculated with the density functionals BP86, PBE0, and B3LYP, respectively.

As expected from the design concept of lowering the steric bulk, both changes in ligand sphere result in thermodynamic profiles that lie overall energetically below those of the original systems, see Figure 7. The isomerization path of complex **3** with the {Me-Et} substitution pattern is consistently situated 1–3 kcal/mol below that of complex **2**. Thus, the formation of intermediate **1** is still the most costly in terms of relative free energies. For complex **4**, however, intermediate 1 lies distinctly lower than intermediate 2, by 2.5 kcal/mol for the PBE0 density functional (2.9 kcal/mol for BP86, 4.2 kcal/mol for B3LYP), therefore achieving a markedly different topology of the isomerization energy surface.

### UV-vis Spectra and Photoactivity

**Figure 8:**
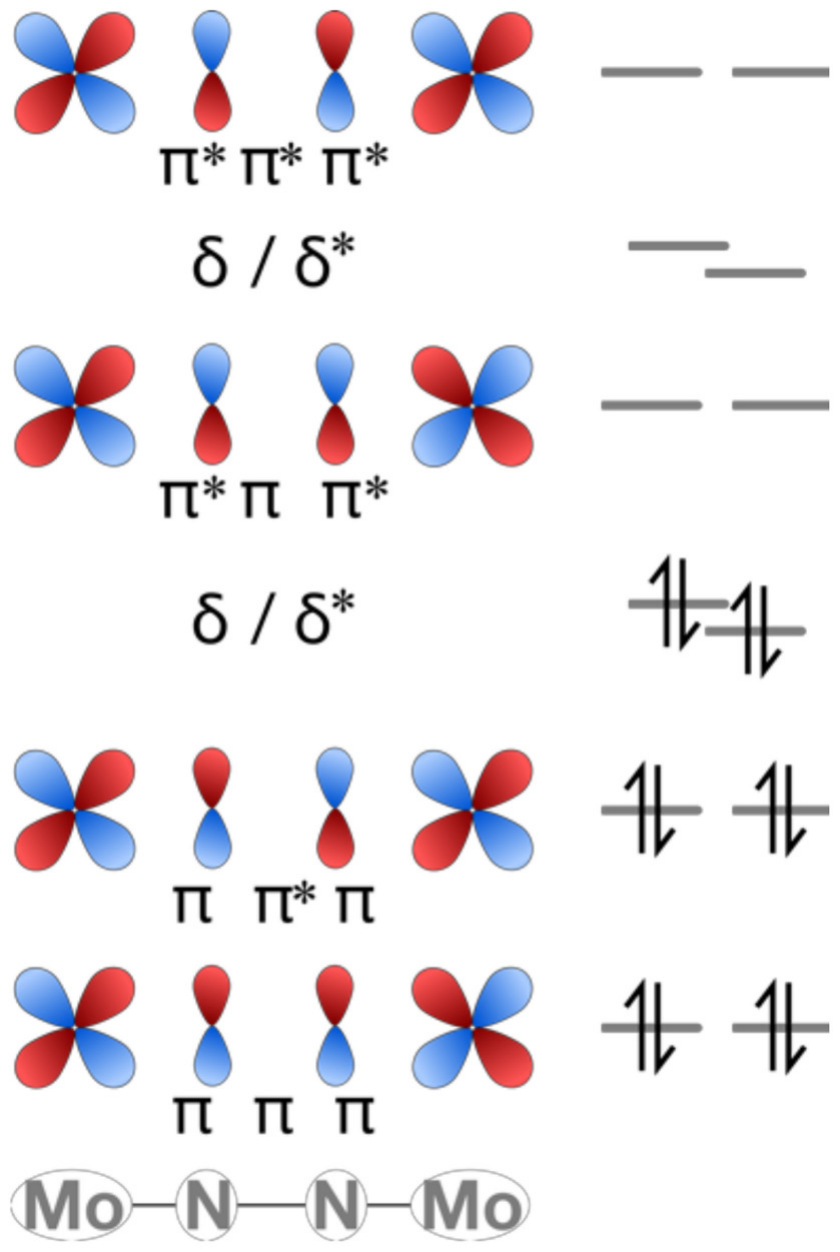
Partial MO diagram of the π and δ manifold for the linear cores of complexes **1** and **2**.

The molecular orbital pattern of the starting compounds is largely as expected for linear M-N-N-M complexes based on a ligand field picture. The d and p valence orbitals of Mo and N form σ, π and δ combinations with alternating bonding and antibonding character between adjacent nuclei, i.e., σ-σ-σ, σ-σ^*^-σ, σ^*^-σ-σ^*^, σ^*^-σ^*^-σ^*^, and similarly for the other molecular orbitals (Krewald and González, 2018). The HOMO and HOMO-1 correspond to δ-orbitals dominated by Mo(d) atomic orbital contributions. The LUMO is of π^*^-π-π^*^ character, i.e., dominated by a π-interaction between μ-N(p) orbitals and π^*^-interaction between μ-N(p) and Mo(d) orbitals. Figure 8 shows the π and δ manifold of the Mo_2_N_2_ core schematically.

To determine any electronic structure differences that may lead to the differences in thermal and photo-reactivity observed for **1** and **2**, their UV-vis spectra are computed and analyzed. While an experimental UV-vis spectrum of **1**_lin_ is published, for **2**_lin_ no literature reference is available. The UV-vis spectra for **1**_lin_ and **2**_lin_ were computed with TD-DFT, testing various GGA, meta-GGA, hybrid and range-separated density functionals to achieve a close agreement with the experimental spectrum (see computational details and Supporting Information).

**Figure 9:**
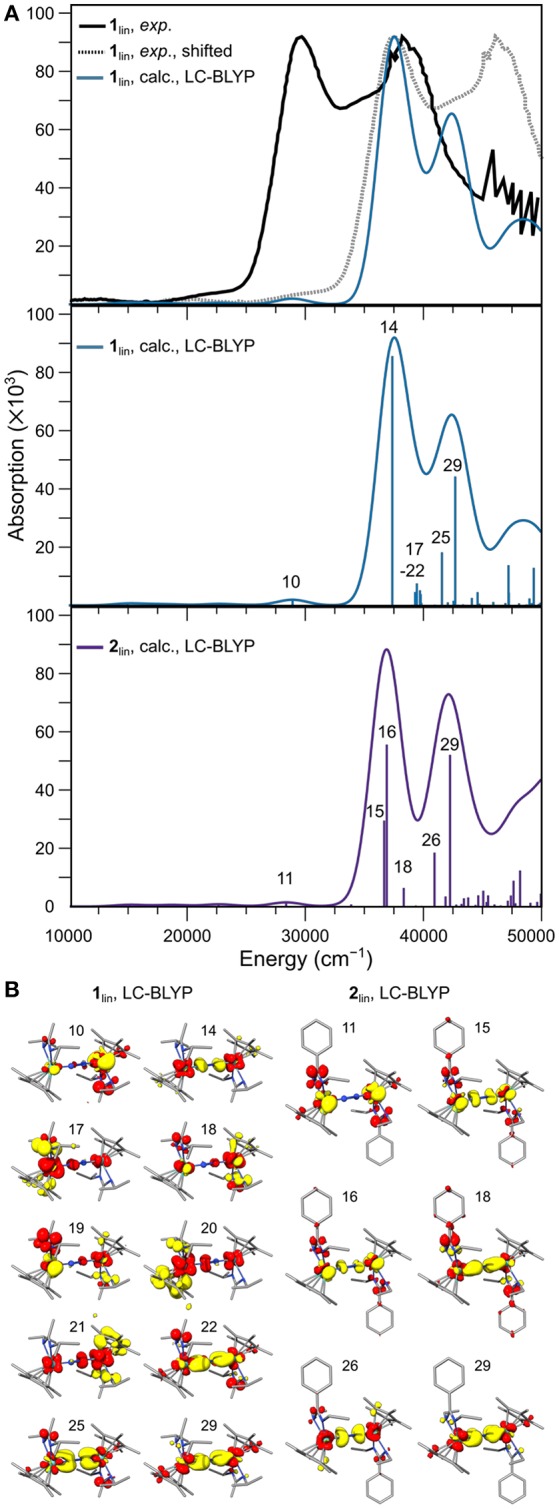
**(A)** Experimental UV-vis spectrum of **1**_lin_ (black: digitized, gray: digitized and blue-shifted) and the broadened line spectrum predicted with TDDFT using the LC-BLYP density functional (top), calculated oscillator strengths and intensities of the individual transitions and broadened line spectrum for **1**_lin_ (middle) and **2**_lin_ (bottom). **(B)** Difference densities for the individual transitions labeled in **(A)**; yellow and red isosurfaces correspond to density loss and gain, respectively.

The best visual agreement between the experimental spectrum of **1**_lin_ and a calculated counterpart was achieved with the range-separated functional LC-BLYP, although it must be recognized that all spectra obtained with TD-DFT are significantly blue-shifted. To facilitate a visual comparison with the digitized experimental spectrum, a gray line spectrum of the experimental data is also blue-shifted by 7,869 cm^−1^ in Figure 9A. It can be seen that the relative energies and intensities of the spectral features at ca. 29,000 cm^−1^ (calc.; exp.: 22,000 cm^−1^) and 37,500 cm^−1^ (calc.; exp.: 30,000 cm^−1^) are reproduced well. The signal at 42,500 cm^−1^ (calc.) would then correspond to the shoulder at 35,800 cm^−1^ (exp.) in the experimental spectrum. The second high-intensity signal at ca. 38,800 cm^−1^ in the experimental spectrum cannot be assigned firmly to the calculated spectral features of **1**_lin_. The predicted spectrum for **2**_lin_ shows overall similar features with a slightly altered intensity distribution.

In the middle and lower panel of Figure 9A, the energies and oscillator strengths of the individual transitions predicted for **1**_lin_ and **2**_lin_ are depicted. At first glance, the line spectra are almost identical. A subtle difference worth noting is a cluster of excitations just below 40,000 cm^−1^ in the calculated spectrum of **1**_lin_, in contrast to a solitary transition at slightly lower energy in the calculated spectrum of **2**_lin_. In both complexes, the low-energy feature at ca. 29,000 cm^−1^ is due to a metal-to-ligand charge transfer excitation from Mo to the amidinate ligand, as assigned based on the difference density (Figure 9B). The first high-intensity feature at ca. 37,500 cm^−1^ in **1**_lin_ can be assigned to an LMCT transition from the dinitrogen bridge to the metal ions (14). In contrast, both intense transitions (15, 16) at the same energy in the spectrum of **2**_lin_ are due to core-to-amidinate excitations. The second intense signal at 42,500 cm^−1^ is dominated by excitations from a π-π^*^-π orbital to molybdenum d and amidinate ligand orbitals. A similar character can be assigned to the corresponding feature in the spectrum of **2**_lin_.

A possibly very relevant difference between the two complexes is found when assessing the character of the six transitions below 40,000 cm^−1^ in the spectrum of **1**_lin_, i.e., transitions 17–22. The first five excitations have difference densities that are distinctly asymmetric. All of them are LMCT transitions, with excitations originating from both amidinate and Cp^*^ ligands. The sixth transition is a core-to-amidinate excitation, similar to the transitions assigned to the higher-energy feature. It is important to stress that the character of the features is not dependent on the density functional chosen for the analysis, but that similar characteristics can be assigned based on other density functionals. As can be seen from a comparison of the difference densities of states 22 (**1**_lin_) and 18 (**2**_lin_), the character of that solitary excitation in the sub-40,000 cm^−1^ energy region in the spectrum of complex **2**_lin_ is almost identical. This difference in the spectra of **1**_lin_ and **2**_lin_, i.e., the fact that there are asymmetric excitations in one complex that are entirely absent in the other, may be the reason that only **1**_lin_ is photoactive (Duman et al., 2016). Sita et al. had put forward the idea that during the photo-driven isomerization and/or dissociation of **1**_lin_, a ligand may have to temporarily detach (Duman et al., 2016). The asymmetric character of the excitations assigned above align with this hypothesis.

## Discussion and Conclusions

The Sita complexes are the most complete series of isostructural dinitrogen-bridged compounds that span groups 4–6 across all transition metal rows: Ti, V; Zr, Nb, Mo; Hf, Ta, W (Hirotsu et al., 2007a,b; Fontaine et al., 2010; Yonke et al., 2011a; Keane et al., 2013, 2014, 2015; Duman et al., 2016; Duman and Sita, 2017). The only missing complex is the Cr dimer, which has been attributed to its much smaller covalent radius than the other early transition metals. Besides a Cp^*^ ligand, one guanidinate or amidinate ligand in one of their five variations, with ^*i*^Pr or Et at the N-donor atoms and NMe_2_, Me, H or Ph at the central carbon, is bound at each metal. This series is of great value for a systematic insight into the fundamental concepts of nitrogen activation due to the variety of metals that is stabilized by ligands of the same family. Comparison of the similarities and differences in this series allows for systematic comparisons of electronic and structural effects in dinitrogen activation. With two members of the series, Sita et al. have shown that by reducing the steric bulk of the substituents in the photochemically active {(η^5^-C_5_Me_5_)[N(^i^Pr)C(Me)N-(^i^Pr)]Mo}_2_(μ-N_2_) (**1**) complex to {(η^5^-C_5_Me_5_)[N(Et)C(Ph)N-(Et)]Mo}_2_(μ-N_2_) (**2**), a thermal pathway to dinitrogen splitting becomes accessible (Keane et al., 2015; Duman et al., 2016). In this paper, computational predictions for likely isomerization intermediates were presented and the thermodynamics of the isomerization reaction were analyzed.

It was confirmed that steric bulk has a significant influence on the thermodynamic profile of the isomerization reactions. The ΔG values for the formation of the intermediates during the isomerization of complex **1** with bulkier ligands were found to be several kcal/mol above those for complex **2** with a sterically less demanding ligand sphere. Furthermore, dispersion interactions along the reaction path appear to be highly relevant factor in the overall driving force of the reaction. In geometry optimizations that omitted dispersion effects, the isomerization of **2** was seen to be energetically disfavored, while no stable intermediates and products were found for the isomerization of **1** without consideration of dispersion effects. In other words, the rearrangement of bonds from approximate double bonds in the linear cores of the starting complexes (Mo-N ca. 1.4, N-N ca. 1.6) and strong single bonds in the diamond-shaped cores of the products (Mo-N ca. 1.2, Mo-Mo ca. 0.7) appears to be almost isoenergetic or perhaps even disfavored. The reactions become overall favorable through the greater dispersion stabilization of the product compared to the starting compound. Since the differences in dispersion correction are approximately equivalent between **1**_lin_/**1**_dia_ and **2**_lin_/**2**_dia_, the remaining difference in reactivity must be attributed to repulsive effects. Based on experimental observations, it is clear that in at least one intermediate along the isomerization pathway of **1**, dispersion effects are not sufficient to overcome steric hindrances. This will be addressed in forthcoming work.

To further explore the concept of reducing the steric requirements of the ligand sphere, *in silico* modifications with {Me-Et} and {Ph-H} were considered. Indeed, the thermodynamics of the reaction path become even more favorable than for the thermally active system {Ph-Et}. For {Ph-H}, the overall energy landscape of the reaction was even altered. However, this also means that changing the ligand substitution pattern can drastically alter the picture that is generated computationally. Especially for reactions with subtle changes in relative energy caution is in order when truncating the ligand system to lower the computational cost. It appears advisable to test the results for artifacts. The change in relative Δ*G* values for the intermediates 1 and 2 obtained with different ligand substitutions shows that this approach can be highly effective in engineering an ideal energy landscape for dinitrogen activation. Besides steric tunic, a future target of computational and experimental studies on this isostructural series will likely be electronic tuning through electron-donating or electron-withdrawing substituents.

To better understand the photoactivity of complex **1**, the UV-vis spectra of **1**_lin_ and **2**_lin_ were computed. Even though the line spectra appear very similar at first glance, the first intense feature is assigned to transitions with different excitation character. In the photoactive complex **1**, there is a single excitation with high oscillator strength due to a LMCT from the bridging N_2_ to the molybdenum ions, i.e., shifting electron density away from the N_2_ unit and hence likely weakening the dinitrogen bond further. In the photochemically inactive complex **2**, there are two transitions of similar MLCT character shifting electron density from the entire Mo_2_N_2_ core to the amidinate ligand. While little is known about the photoactive states in dinitrogen photoactivation complexes, a recent study on a rhenium dimer with a PNP pincer ligand (Schendzielorz et al., 2019) identified states in the photoactive region of μ-N_2_-to-metal character, i.e., similar to the high-intensity excitation in complex **1**. A similar excitation character was also found for a N_2_-bridged photoactive molybdenum pincer complex by Nishibayashi et al. (Miyazaki et al., 2014). If it were confirmed experimentally that irradiation of *only* the first signal results in photoactivation of complex **1**, a general pattern regarding the requirements for successful N_2_ photoactivation could emerge that is independent of the ligand platform and metal chosen.

However, the spectrum of complex **1**_lin_ also contains a cluster of medium-high intensity transitions that are *not* predicted for the photo-inactive complex **2**_lin_. These are due to asymmetric excitations that originate from the ligand sphere of only one molybdenum ion and shift density into the Mo-N-N-Mo π system. The character of these excitations is in line with Sita's hypothesis of a temporary, photo-induced ligand detachment being required for isomerization of the molybdenum dimer and splitting of the N-N bond (Duman et al., 2016).

A better understanding of dinitrogen photoactivation in general will require more spectroscopic studies on the character of the photoactive states and the excited state decay. Additionally, an exhaustive comparison of the photo-activation and thermal activation pathways for the isostructural complexes in Sita's series requires the character(s) of the specific photoactive state(s) to be known so that its features can be related to the transition state structure of the thermal path. However, already at this point, it is interesting to see that despite the geometric similarities of the two complexes, the ligand sphere appears to have a distinct influence on their electronic structures that is mapped in the computed excitation spectra. Further computational and experimental studies, especially in terms of time-resolved spectroscopy, are required to fully elucidate these subtle differences.

## Data Availability

The raw data supporting the conclusions of this manuscript will be made available by the author, without undue reservation, to any qualified researcher.

## Author Contributions

VK designed the research, carried out the required calculations and analyses, and wrote the paper.

### Conflict of Interest Statement

The author declares that the research was conducted in the absence of any commercial or financial relationships that could be construed as a potential conflict of interest.
