# Supplementary material for: Steric Switching From Photochemical to Thermal N2 Splitting: A Computational Analysis of the Isomerization Reaction {(Cp*)(Am)Mo}2(μ-η1:η1-N2) → {(Cp*)(Am)Mo}2(μ-N)2
Source: Front Chem. 2019 May 16;7:352. doi: 10.3389/fchem.2019.00352 (PMC6535493; doi:10.3389/fchem.2019.00352)
Supplement: Supplementary file 1 [file Data_Sheet_1.PDF]

Supporting Information for

Steric Switching From Photochemical to Thermal N2 Splitting: A Computational Analysis of the Isomerization Reaction {(Cp*)(Am)Mo}$_2$(μ-η1:η1-N2) → {(Cp*)(Am)Mo}$_2$(μ-N)2

Vera Krewald[1*]

[1]Theoretische Chemie, Fachbereich Chemie, TU Darmstadt, Alarich-Weiss-Str. 4, 64287 Darmstadt, Germany

*** Correspondence:**
Vera Krewald, krewald@chemie.tu-darmstadt.de

Contents:

Table S1. Key interatomic distances of complexes **1**, **2**, **3**, **4** in all their isomeric forms (lin, int-1, int-2, dia). **p.2**

Table S2. Key angles in the cores of complexes **1**, **2**, **3**, **4** in all their isomeric forms (lin, int-1, int-2, dia). **p.3**

Table S3. H-H pairs of intermediates of **1**: numbers, combined lengths (Å) and average distances (Å). **p.4**

Table S4. H-H pairs of intermediates of **2**: numbers, combined lengths (Å) and average distances (Å). **p.5**

Table S5. Relative final single point energies (kcal/mol) along the isomerisation coordinate of complexes **1**, **2**, **3** and **4** with the density functionals BP86, PBE0, B3LYP. **p.6**

Table S6. Relative final single point energies (kcal/mol) and relative Δ*G* values (kcal/mol) for the isomerisation coordinate of complex 2 without consideration of dispersion corrections. **p.6**

Table S7. Relative Δ*G* values (kcal/mol) for the isomerisation coordinate of complexes **1**, **2**, **3** and **4** with thermodynamic parameters calculated with the BP86 density functional and electronic structure calculations with the density functionals BP86, PBE0, B3LYP. **p.7**

Table S8. Computed Mayer bond orders along the isomerisation coordinate of **3** and **4**. **p.7**

Figure S1. TD-DFT spectra for complex $\mathbf{1}_{lin}$ calculated with various density functionals **p.8**

Figure S2. TD-DFT spectra for complex $\mathbf{2}_{lin}$ calculated with various density functionals **p.9**

Cartesian coordinates of all complexes **p.10ff**

Table S1. Key interatomic distances (Å) of complexes **1**, **2**, **3**, **4** in all their isomeric forms (lin, int-1, int-2, dia).

Cpd.	Mo-Mo	Mo-N	Mo-N	Mo-N	Mo-N	N-N	M-N_L	M-N_L	M-N_L	M-N_L
$\mathbf{1}_{lin}$	4.85	1.81	3.05	1.81	3.05	1.25	2.15	2.14	2.14	2.14
$\mathbf{1}_{int\text{-}1}$	4.45	1.84	3.00	1.89	2.27	1.30	2.12	2.15	2.20	2.14
$\mathbf{1}_{int\text{-}2}$	3.74	1.89	2.11	1.90	2.07	1.40	2.16	2.14	2.18	2.14
$\mathbf{1}_{dia}$ (s)	2.63	1.90	1.90	1.90	1.90	2.74	2.23	2.23	2.23	2.23
$\mathbf{1}_{dia}$ (t)	2.77	1.82	1.82	1.98	1.98	2.59	2.27	2.28	2.19	2.19
$\mathbf{2}_{lin}$	4.86	1.81	3.05	1.81	3.05	1.25	2.14	2.13	2.13	2.14
$\mathbf{2}_{int\text{-}1}$	3.77	1.86	3.08	1.93	2.14	1.30	2.12	2.20	2.10	2.17
$\mathbf{2}_{int\text{-}2}$	3.73	2.07	1.90	1.91	2.09	1.40	2.15	2.14	2.13	2.15
$\mathbf{2}_{dia}$ (s)	2.62	1.92	1.89	1.89	1.92	2.77	2.20	2.22	2.22	2.20
$\mathbf{2}_{dia}$ (t)	2.76	1.81	1.84	2.00	1.97	2.61	2.27	2.23	2.16	2.15
$\mathbf{3}_{lin}$	4.86	1.81	3.05	1.81	3.05	1.25	2.12	2.12	2.12	2.12
$\mathbf{3}_{int\text{-}1}$	3.66	1.85	3.08	1.93	2.12	1.31	2.15	2.12	2.16	2.10
$\mathbf{3}_{int\text{-}2}$	3.72	1.89	2.08	1.89	2.08	1.41	2.15	2.14	2.14	2.15
$\mathbf{3}_{dia}$	2.62	1.91	1.90	1.91	1.90	2.77	2.20	2.22	2.22	2.20
$\mathbf{4}_{lin}$	4.83	1.81	3.03	1.81	3.03	1.24	2.09	2.13	2.09	2.13
$\mathbf{4}_{int\text{-}1}$	3.55	1.84	3.10	1.94	2.16	1.29	2.16	2.11	2.10	2.17
$\mathbf{4}_{int\text{-}2}$	3.72	1.90	2.06	1.90	2.06	1.40	2.14	2.15	2.15	2.14
$\mathbf{4}_{dia}$	2.63	2.03	1.85	1.80	1.95	2.72	2.12	2.24	2.13	2.36

Table S2. Key angles (°) in the cores of complexes **1**, **2**, **3**, **4** in all their isomeric forms (lin, int-1, int-2, dia).

Cpd.	MNN	MNN	MNM	MNM	NMMN
$\mathbf{1}_{lin}$	176.3	176.7	177.8	178.3	-91.5
$\mathbf{1}_{int-1}$	144.6	88.8	158.4	109.5	-10.4
$\mathbf{1}_{int-2}$	78.2	75.9	140.9	136.9	-164.0
$\mathbf{1}_{dia}$ (s)	43.8	43.8	87.6	87.6	-180.0
$\mathbf{1}_{dia}$ (t)	44.6	49.1	93.5	93.5	173.2
$\mathbf{2}_{lin}$	179.1	179.1	179.7	179.6	173.0
$\mathbf{2}_{int-1}$	154.3	80.6	140.9	94.9	10.0
$\mathbf{2}_{int-2}$	76.1	76.5	138.8	139.3	-176.7
$\mathbf{2}_{dia}$ (s)	43.0	43.9	86.9	86.9	-180.0
$\mathbf{2}_{dia}$ (t)	44.6	49.3	92.8	93.1	-174.6
$\mathbf{3}_{lin}$	177.2	177.2	178.9	178.8	176.7
$\mathbf{3}_{int-1}$	153.7	79.4	134.1	90.9	17.4
$\mathbf{3}_{int-2}$	76.5	76.5	138.7	138.7	180.0
$\mathbf{3}_{dia}$	43.7	43.7	86.9	86.9	-180.0
$\mathbf{4}_{lin}$	163.3	163.5	171.4	171.7	102.9
$\mathbf{4}_{int-1}$	162.5	81.5	124.8	86.5	12.6
$\mathbf{4}_{int-2}$	75.6	75.5	138.9	138.8	179.9
$\mathbf{4}_{dia}$	43.0	41.5	86.5	87.5	-157.5

Table S3. H-H pairs of intermediates for complex **1**: numbers, combined lengths (Å) and average distances (Å).

	bin	# H-H pairs	Combined length (Å)	Average length (Å)
$\mathbf{1}_{lin}$	1.8–2.2	*18*	35.49	1.97
	2.2–2.6	*43*	105.43	2.45
	2.6–3.0	*32*	87.81	2.74
	3.0–3.4	*47*	150.52	3.20
	3.4–3.8	*59*	213.14	3.61
	3.8–4.2	*87*	346.36	3.98
	**total**	***286***	**938.74**	
$\mathbf{1}_{int\text{-}1}$	1.8–2.2	*19*	38.10	2.01
	2.2–2.6	*51*	123.10	2.41
	2.6–3.0	*23*	64.41	2.80
	3.0–3.4	*49*	156.07	3.19
	3.4–3.8	*70*	253.24	3.62
	3.8–4.2	*93*	365.42	3.93
	**total**	***305***	**1000.34**	
$\mathbf{1}_{int\text{-}2}$	1.8–2.2	*20*	39.72	1.99
	2.2–2.6	*55*	134.24	2.44
	2.6–3.0	*26*	73.12	2.81
	3.0–3.4	*44*	140.49	3.19
	3.4–3.8	*84*	307.08	3.66
	3.8–4.2	*74*	294.47	3.98
	**total**	***303***	**989.12**	
$\mathbf{1}_{dia}$	1.8–2.2	*22*	43.84	1.99
	2.2–2.6	*54*	133.29	2.47
	2.6–3.0	*40*	111.05	2.78
	3.0–3.4	*32*	102.12	3.19
	3.4–3.8	*88*	321.32	3.65
	3.8–4.2	*94*	375.08	3.99
	**total**	***330***	**1086.71**	

Table S4. H-H pairs of intermediates for complex **2**: numbers, combined lengths (Å) and average distances (Å).

	bin	# H-H pairs	Combined length (Å)	Average length (Å)
$\mathbf{2}_{lin}$	1.8–2.2	*10*	19.45	1.95
	2.2–2.6	*44*	106.81	2.43
	2.6–3.0	*14*	39.33	2.81
	3.0–3.4	*38*	122.14	3.21
	3.4–3.8	*46*	167.05	3.63
	3.8–4.2	*58*	229.48	3.96
	**total**	***210***	**684.27**	
$\mathbf{2}_{int\text{-}1}$	1.8–2.2	*8*	15.14	1.89
	2.2–2.6	*52*	126.75	2.44
	2.6–3.0	*12*	33.21	2.77
	3.0–3.4	*39*	125.09	3.21
	3.4–3.8	*49*	177.33	3.62
	3.8–4.2	*61*	243.44	3.99
	**total**	***221***	**720.95**	
$\mathbf{2}_{int\text{-}2}$	1.8–2.2	*14*	*27.59*	*1.97*
	2.2–2.6	*50*	*122.31*	*2.45*
	2.6–3.0	*17*	*46.90*	*2.76*
	3.0–3.4	*41*	*132.39*	*3.23*
	3.4–3.8	*42*	*153.20*	*3.65*
	3.8–4.2	*75*	*299.28*	*3.99*
	**total**	***239***	***781.67***	
$\mathbf{2}_{dia}$	1.8–2.2	*18*	36.24	2.01
	2.2–2.6	*52*	130.00	2.50
	2.6–3.0	*26*	72.35	2.78
	3.0–3.4	*30*	95.75	3.19
	3.4–3.8	*44*	160.24	3.64
	3.8–4.2	*66*	263.40	3.99
	**total**	***236***	**757.99**	

Table S5. Relative final single point energies (kcal/mol) along the isomerisation coordinate of complexes **1**, **2**, **3** and **4** with the density functionals BP86, PBE0, B3LYP.

	E_{rel} (kcal/mol) [BP86]	E_{rel} (kcal/mol) [PBE0]	E_{rel} (kcal/mol) [B3LYP]
$\mathbf{1}_{lin}$	0.00	0.00	0.00
$\mathbf{1}_{int-1}$	23.64	23.75	23.21
$\mathbf{1}_{int-2}$	20.54	22.91	24.31
$\mathbf{1}_{dia}$ (s)	-16.36	-1.30	-6.25
$\mathbf{1}_{dia}$ (t)	-8.94		
$\mathbf{2}_{lin}$	0.00	0.00	0.00
$\mathbf{2}_{int-1}$	17.22	18.63	18.89
$\mathbf{2}_{int-2}$	10.75	14.15	15.92
$\mathbf{2}_{dia}$ (s)	-22.94	-7.76	-12.46
$\mathbf{2}_{dia}$ (t)	-14.66		
$\mathbf{3}_{lin}$	0.00	0.00	0.00
$\mathbf{3}_{int-1}$	13.51	15.44	15.19
$\mathbf{3}_{int-2}$	11.68	13.22	14.81
$\mathbf{3}_{dia}$	-21.68	-7.96	-12.66
$\mathbf{4}_{lin}$	0.00	0.00	0.00
$\mathbf{4}_{int-1}$	13.40	12.16	10.75
$\mathbf{4}_{int-2}$	19.51	16.65	17.02
$\mathbf{4}_{dia}$	-18.50	-12.45	-18.22

Table S6. Relative final single point energies (kcal/mol) and relative ΔG values (kcal/mol) for the isomerisation coordinate of complex 2 without consideration of dispersion corrections.

	E_{rel} (kcal/mol) [BP86]	E_{rel} (kcal/mol) [PBE0]	E_{rel} (kcal/mol) [B3LYP]	ΔG_{rel} (kcal/mol) [BP86]	ΔG_{rel} (kcal/mol) [PBE0]	ΔG_{rel} (kcal/mol) [B3LYP]
$\mathbf{2}_{lin}$	0.00	0.00	0.00	0.00	0.00	0.00
$\mathbf{2}_{int-1}$	26.99	24.57	28.07	28.92	26.03	29.53
$\mathbf{2}_{int-2}$	21.81	20.39	26.04	22.95	21.73	27.38
$\mathbf{2}_{dia}$ (s)	1.16	6.95	11.06	4.10	9.59	13.69

Table S7. Relative Δ*G* values (kcal/mol) for the isomerisation coordinate of complexes **1**, **2**, **3** and **4** with thermodynamic parameters calculated with the BP86 density functional and electronic structure calculations with the density functionals BP86, PBE0, B3LYP.

	ΔG_{rel} (kcal/mol) [BP86]	ΔG_{rel} (kcal/mol) [PBE0]	ΔG_{rel} (kcal/mol) [B3LYP]
**1**$_{lin}$	0.00	0.00	0.00
**1**$_{int-1}$	23.47	24.09	23.55
**1**$_{int-2}$	21.66	24.50	25.90
**1**$_{dia}$ (s)	-14.31	1.03	-3.92
**2**$_{lin}$	0.00	0.00	0.00
**2**$_{int-1}$	19.13	20.49	20.75
**2**$_{int-2}$	11.77	15.27	17.04
**2**$_{dia}$ (s)	-18.65	-3.77	-8.46
**3**$_{lin}$	0.00	0.00	0.00
**3**$_{int-1}$	16.30	17.79	17.53
**3**$_{int-2}$	11.30	13.50	15.09
**3**$_{dia}$	-18.92	-5.03	-9.74
**4**$_{lin}$	0.00	0.00	0.00
**4**$_{int-1}$	14.31	11.50	10.08
**4**$_{int-2}$	17.22	13.99	14.35
**4**$_{dia}$	-20.28	-14.58	-20.35

Table S8. Computed Mayer bond orders along the isomerisation coordinate of **3** and **4**.

	Mo-Mo	Mo-N	Mo-N	Mo-N	Mo-N	N-N
**3**$_{lin}$	0.30	1.41	0.12	0.12	1.41	1.60
**3**$_{int-1}$	0.30	1.13	0.16	0.51	1.19	1.05
**3**$_{int-2}$	0.33	0.65	1.13	1.13	0.65	0.86
**3**$_{dia}$	0.71	1.14	1.35	1.35	1.14	<0.1
**4**$_{lin}$	0.28	1.32	0.12	0.12	1.32	1.52
**4**$_{int-1}$	0.30	1.16	0.16	0.49	1.23	1.07
**4**$_{int-2}$	0.34	0.69	1.14	1.14	0.69	0.86
**4**$_{dia}$	0.73	1.24	1.28	1.28	1.24	<0.1

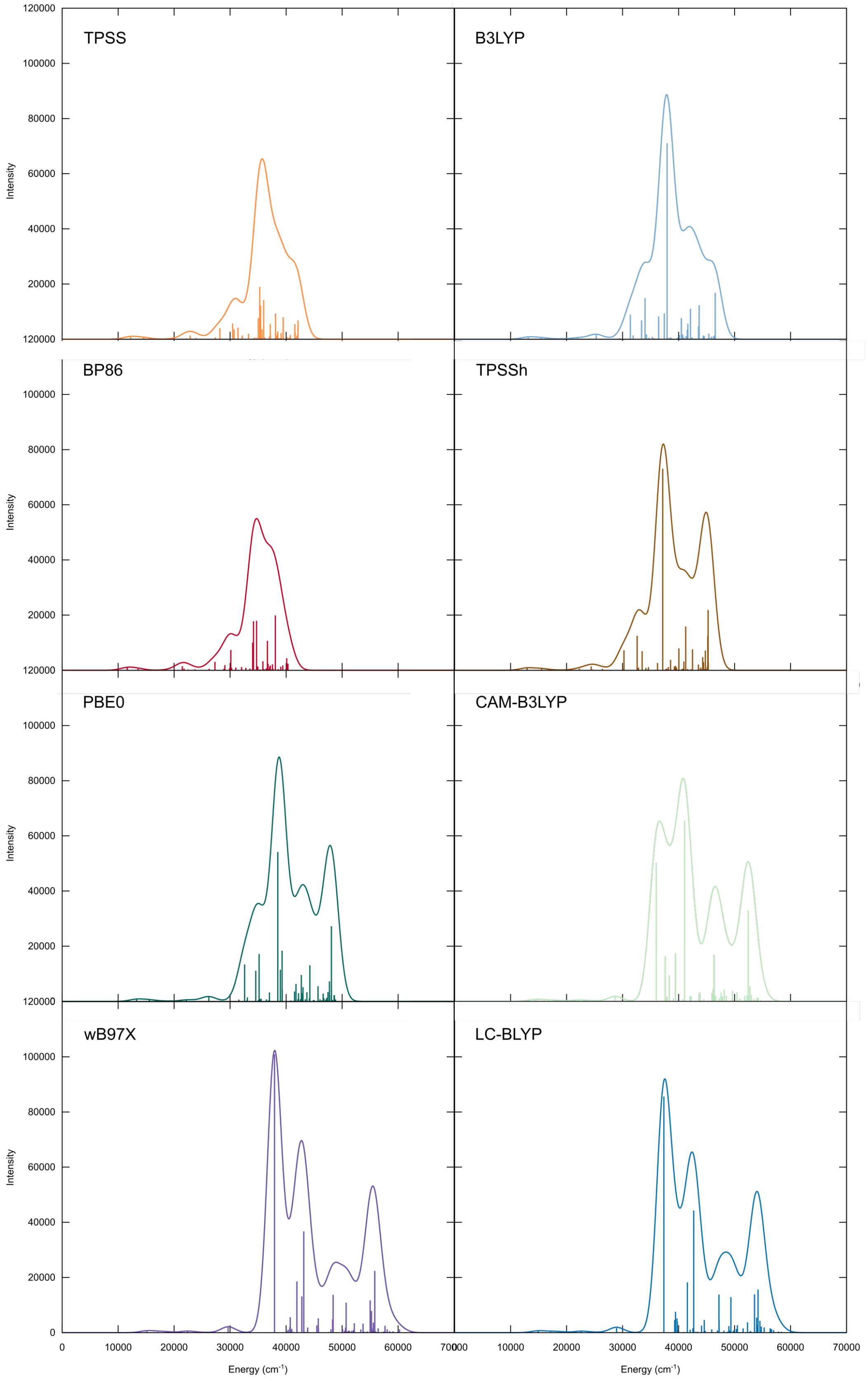

Figure S1. Spectra predicted for complex $\mathbf{1}_{\mathrm{lin}}$ with various density functionals.

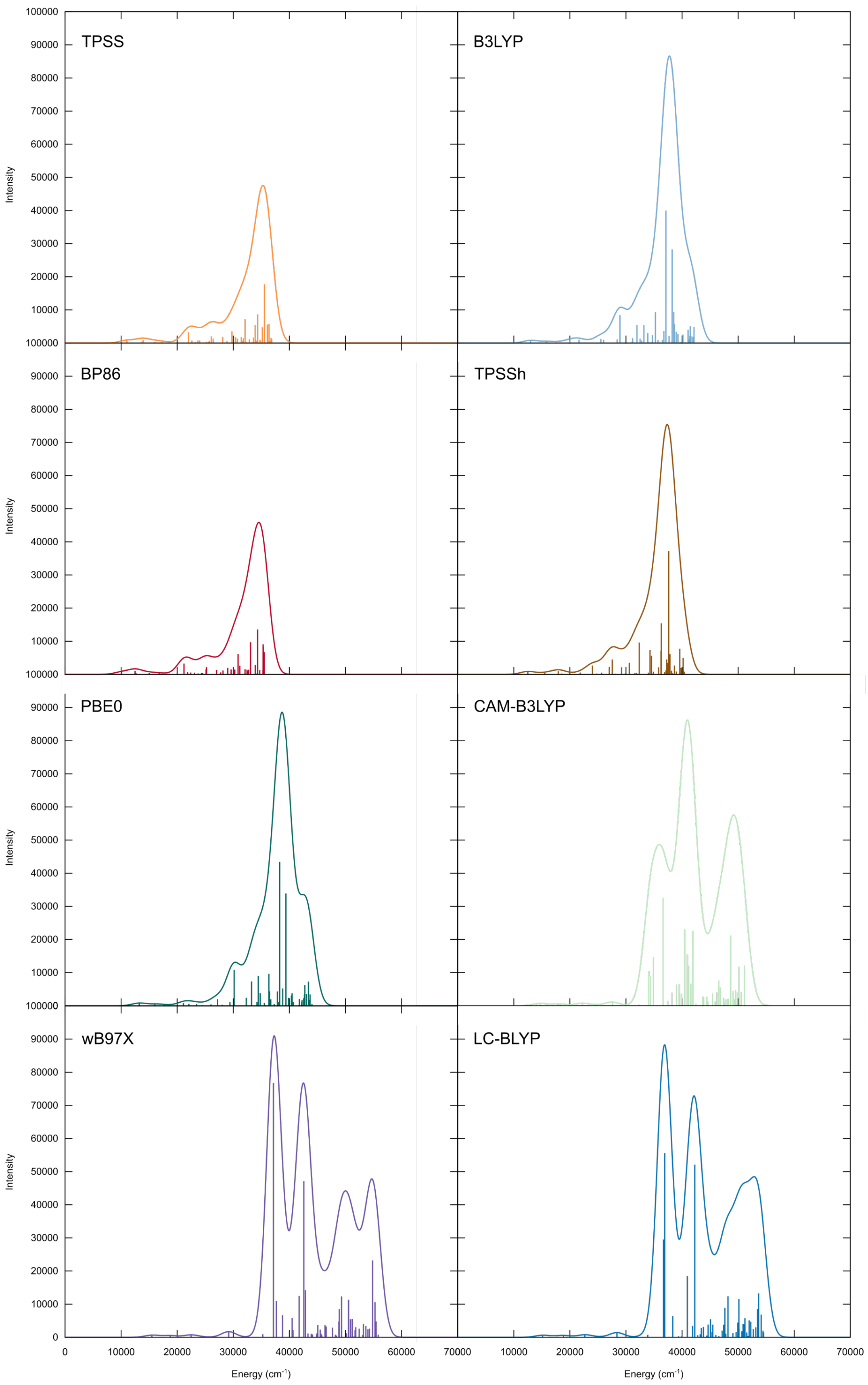

Figure S1. Spectra predicted for complex $\mathbf{2}_{\text{lin}}$ with various density functionals.

Cartesian Coordinates of all complexes

108

1_lin

Mo 6.94286343851745 5.57436692020574 14.73340175254993
Mo 10.49590957197906 4.36779487341300 17.81364805821962
N 9.15865247325316 4.78810305071939 16.67624454405195
N 8.28476317428529 5.10619216991714 15.84636032294661
N 5.41676057717216 4.17847606345688 15.30656393497484
N 5.47510294950904 6.23121672435096 16.13918955391614
N 12.02963374272962 5.69939216571719 17.15411418038125
N 11.96462702804298 3.59501824554612 16.45642194754285
C 5.10258878228293 4.95853500113542 16.35790091801538
C 4.51240373780483 4.45948316856648 17.65383817877587
H 4.05677427726828 3.46104952645950 17.53116245715702
H 3.73762397036737 5.15536512528386 18.02869855924603
H 5.29825149679741 4.38398681041381 18.43205981834168
C 5.39641919775162 2.71547367294961 15.38066201571021
H 4.47464654932428 2.40176087174785 15.92347981270811
C 5.32808109737071 2.12732055088941 13.97140724261433
H 6.20609973281057 2.46613856802632 13.38715452779013
H 4.41329779345468 2.45790948406387 13.44359261672389
H 5.33353795705633 1.01940313456842 14.00674290324400
C 6.61784129452881 2.17822031923148 16.14496459380552
H 6.66112909707272 2.58718335437916 17.17301606005530
H 7.54951564722479 2.49365488261165 15.63394671113851
H 6.59545062693504 1.07098052654104 16.21058735988805
C 5.47873443350769 7.24807935118584 17.19408837376632
H 4.91237944110193 6.85655336519100 18.06743831738190
C 4.77218910405285 8.52339129345846 16.72145749334470
H 3.73888428895817 8.30750768171868 16.38516666397093
H 5.32057725095027 8.97382577155655 15.87098503711706
H 4.72798272464549 9.27320179454104 17.53760307673555
C 6.91318953309397 7.54285630812820 17.65708049167630
H 7.51178686718517 7.93304043822617 16.81080379703907
H 7.42087016970980 6.62294223040710 18.00586878650575
H 6.91724730497701 8.28929993765658 18.47830607989092
C 8.07693533056618 6.33711515844007 13.04896776988337
C 7.19484319658364 7.43058202294481 13.46734678786137
C 5.85289578428626 7.02969382133713 13.08712397428891
C 5.89592703487840 5.73701577631006 12.49895042289437
C 7.26062463055458 5.26392351294355 12.47944917852776
C 9.57914552497881 6.38542415879720 13.03328082427797
H 9.96537471470469 6.81823635246230 12.08384516180884
H 10.01278202145534 5.37208898070887 13.13949078080198
H 9.97146742461407 7.00052275815621 13.86652862762155
C 7.63384158936041 8.80689260578339 13.87906697578384
H 6.88218365042183 9.29786338989118 14.52799167547094
H 7.79624442859233 9.47117255706456 12.99891722210897
H 8.58296573541884 8.77396421385442 14.44868824878280
C 4.59090195371025 7.81930261874960 13.27661170463670
H 3.87577396169709 7.29519554424866 13.94315560639310
H 4.07989567302121 7.98202955302096 12.30352083350886
H 4.79231120792047 8.81145902529753 13.71991886647001
C 4.68430732663593 5.00070479588120 12.00966214837193
H 4.94170170151547 3.99694458560844 11.62491464355985
H 4.19026895925532 5.56184484566834 11.18754698010843
H 3.93508708178704 4.87161250819551 12.81752000154566
C 7.79088267598854 4.07235544376350 11.73389976511626
H 8.09607707567362 4.34481304918375 10.69724709313943
H 7.03184138629987 3.27029355298458 11.65142048286861
H 8.67739365871194 3.63788662944757 12.23545442728755
C 12.32434090524294 4.85569856957848 16.14910065157070
C 12.87807884806210 5.29228514562787 14.81524243923786
H 13.70271330083093 6.01828016899089 14.95012511028206
H 13.25968906521157 4.43352939520354 14.23536286586266
H 12.09043463750945 5.78949755898929 14.21457051433494
C 12.09104171364528 7.15606037538759 17.00386060542870
H 12.68788994180169 7.39390830206292 16.09604100525779
C 12.80359222668058 7.79225609187603 18.20184157797741
H 12.23057709995255 7.60729319859223 19.13146052967315
H 13.81789185406583 7.36753813370666 18.33615452448129
H 12.89545739965090 8.88927150840686 18.06683482374621
C 10.68485342631950 7.74433321729666 16.81334050739434
H 10.17086711601779 7.28838964607575 15.94519706838324
H 10.06000883737543 7.52514469216154 17.70125111097497
H 10.73295189935508 8.84313665592077 16.66516218755828
C 11.91139012278906 2.52814560209823 15.45333520933872
H 12.81850256653784 2.60414414106042 14.80989843711121
C 11.94796451644206 1.16628519378110 16.14675629415222
H 12.87473504515700 1.04363106185047 16.73909569692839
H 11.08380356179979 1.07832707639078 16.83407039875267
H 11.89290838746011 0.34516085161834 15.40409127398779
C 10.66769404244673 2.65972388641010 14.55865225124317
H 9.75041211459392 2.59900182868477 15.17775989694044
H 10.64242939246182 3.63991603302078 14.04415875088611
H 10.64106271447443 1.86058181149395 13.78954605463615
C 9.34638985154173 3.91585839948768 19.59967003028094
C 10.10208886950362 2.69146872953877 19.33327254292029
C 11.48560110743268 2.99795802221023 19.61548053220132
C 11.59672055727868 4.35961444842767 20.00660095455795
C 10.28311934890684 4.97473267030196 19.98492475092709
C 7.84760595788947 4.01713671828614 19.65000574089909
H 7.45359564962951 3.76164868550577 20.65865468842344
H 7.50324294258118 5.04059964467453 19.40409454121727
H 7.37684662431009 3.32796400187469 18.92239997738523
C 9.50388874809146 1.32834073491662 19.13189032324602
H 10.22414807149382 0.63035125228992 18.66258687835450
H 9.19011144966282 0.87528727950134 20.10055566910055
H 8.60958853383294 1.36728588417509 18.48014061846964
C 12.65240322801095 2.06233357364389 19.50116872489611
H 13.41702139515379 2.45173209417563 18.79811083163203
H 13.14697287537819 1.93043736439607 20.48752925767835
H 12.34353582076304 1.06317679824137 19.14327788509735
C 12.89444387754409 5.02258529518709 20.36516663332094
H 12.74392594584715 6.07073535580375 20.68184089980776
H 13.39180641707774 4.48542167433840 21.20094026307866
H 13.60022653201067 5.03060856892112 19.50942246288538
C 9.90951975811597 6.30963851769112 20.56372136255970
H 9.75137136181042 6.25431497866504 21.66561398419892
H 10.69550893912455 7.06857271310284 20.38117699286121

H 8.97425597977058 6.69590107735058 20.11378660751535

108

1_int-1

Mo 6.03610991341582 5.89347352015484 14.28792543854402
Mo 8.95135449428415 5.50364880866018 17.06833961130963
N 7.46610987387152 4.47250692388536 16.51257919446018
N 7.23099800108972 5.35794547144911 15.58731700778875
N 4.71636896927178 4.32784075083445 14.82992030491736
N 4.42118533558594 6.37421462236478 15.62796143693567
N 9.48336399405113 7.55424187161701 16.49384525614687
N 10.32279102199592 5.82591476428802 15.46298670465550
C 4.17863823400634 5.07249702301153 15.81809725706714
C 3.52317829618525 4.48522102080777 17.04252964052685
H 2.86460595268600 3.63671818340018 16.78048638404093
H 2.92603427200321 5.24205059277316 17.58205113852501
H 4.30188697568910 4.10426116259582 17.73529545507638
C 4.82285949195775 2.86641054613771 14.97451797268307
H 4.92435234635420 2.63154934930892 16.05788795535144
C 3.56518435582989 2.15294192832973 14.44941804953948
H 3.47581883005286 2.27244577388779 13.35240175961603
H 2.64200176993442 2.56207537467825 14.90706581662423
H 3.60778899611018 1.06754897094735 14.67711031078647
C 6.08887377488219 2.34122984820485 14.29844462321004
H 6.98453695121052 2.83608125992735 14.71788236191915
H 6.06330067550307 2.53169230324607 13.20933201777764
H 6.17581092967485 1.24662402770088 14.45649578633960
C 4.18315233285005 7.37534988029070 16.66600800419333
H 3.17719072673243 7.18962800397635 17.11167756395752
C 4.15981897162529 8.77039289994591 16.04064048096786
H 3.38195043364916 8.83896057872810 15.25510609937228
H 5.13766983426381 8.99146071567170 15.57181542865855
H 3.95499262707452 9.54360442042563 16.80813213773807
C 5.23845429740026 7.27659287860462 17.78052943853343
H 6.25693809609370 7.32025399238748 17.34494676860126
H 5.17053352355136 6.31134800430980 18.31626396191932
H 5.12412746940550 8.09515148337238 18.52084885755249
C 7.02877327837992 6.67475125571571 12.50718000811653
C 5.87384909133763 7.55187952327620 12.75808908811439
C 4.69367684951560 6.76242599807061 12.46559187409296
C 5.10020284004960 5.46501504861576 12.04137125141644
C 6.53623517968985 5.37810147988146 12.06771584019767
C 8.46403065299441 7.11770552621958 12.51073694319281
H 8.70827048753572 7.72505988877144 11.61062242575614
H 9.14796628007601 6.24893041112990 12.52833368447906
H 8.69713417068687 7.73357961274998 13.39972452900348
C 5.90997531056243 9.05094317196772 12.84568016262424
H 5.01095982514177 9.45533633227281 13.34861804844282
H 5.95103456590597 9.50478566549593 11.82877188565815
H 6.79289065991960 9.41531493336730 13.40078575974395
C 3.26933980178218 7.21572264159089 12.58834109426008
H 2.64717803756912 6.45102621148657 13.09676499908221
H 2.81270285381160 7.41045976114477 11.59323303362195
H 3.19413157299960 8.14687316506727 13.18016490051517
C 4.15851988430032 4.37843800018513 11.62837628665206
H 4.63718272237701 3.38181649737039 11.66348331583166
H 3.80728770235897 4.54535512044297 10.58694368648634
H 3.26532862499883 4.35215245809220 12.28204396705445
C 7.37987297713816 4.27468627948241 11.49962459491331
H 7.66337525887279 4.49018616696069 10.44452864983562
H 6.84573312583966 3.30506017335333 11.50243598850552
H 8.31696888579282 4.13618469143080 12.07241146718359
C 10.33733513994487 7.16286371909219 15.54238225948086
C 11.21826918666110 8.07052593811684 14.71916111573738
H 12.15391029971030 8.30378971770103 15.27045893693871
H 11.50194797062371 7.58890393493328 13.76503539929313
H 10.72090645645315 9.03049945811550 14.48769475577970
C 9.13163887754571 8.95784365756789 16.69591859395214
H 10.04222250767496 9.58559687632883 16.54990551606524
C 8.63063391897676 9.19938465292076 18.11654260153597
H 7.77124046008484 8.53798672061495 18.33401059580822
H 9.42198513386621 8.98269000568144 18.85787725569097
H 8.31319388999780 10.25371004477264 18.24530609825025
C 8.07858918707075 9.38474011937935 15.66224372514688
H 8.50213773158273 9.37427348652562 14.63898641506279
H 7.23433687689508 8.66534601454272 15.67583364352707
H 7.68743557128190 10.40410953571994 15.85914935034289
C 10.96278857549631 5.04284823476606 14.41331789004755
H 10.82901454180357 5.59283581114703 13.45110231813597
C 12.47545832831232 4.84843411708769 14.60988744622851
H 12.99378234310181 5.82009475900612 14.73376399086772
H 12.68109287136490 4.23643861691824 15.50691596843610
H 12.91975129542801 4.33472519249426 13.73181467909080
C 10.21695998851443 3.71175982822096 14.27981934413444
H 10.29554909183261 3.12924241932972 15.21888954159876
H 9.13891291619819 3.89675807563084 14.10402097248411
H 10.62560801640799 3.10599481794069 13.44587102145774
C 8.28465131813223 4.44743669400614 19.09334415391523
C 9.20537103745901 3.54961483626304 18.45202100711468
C 10.46020724591750 4.22224875368544 18.30891028355717
C 10.30314408551712 5.54966253984280 18.86464108914700
C 8.93817285389157 5.70119479600385 19.36784814249016
C 6.87874029421924 4.08739816749810 19.46668104316875
H 6.86420014802975 3.41593138002241 20.35282983008316
H 6.28072653937370 4.98550692604279 19.71158927695353
H 6.37833233614523 3.55863269841820 18.63062705025141
C 8.87421312256824 2.15554607063269 18.01617734376539
H 9.65873537126045 1.73755988514360 17.35622088781165
H 8.76684166056371 1.47526949877916 18.88905594383376
H 7.91616092041928 2.14521680880325 17.45714807901215
C 11.75593039596338 3.59277564077683 17.89916493273013
H 12.47954534683173 4.35075084761858 17.54634740363269
H 12.22174814039011 3.07001190349964 18.76416390408068
H 11.62129329583314 2.84583333317251 17.09300309412814
C 11.40433461077914 6.56743728421232 18.97742867302634
H 10.99765215483795 7.59320482632057 19.05544213769639
H 12.03868871992278 6.38597515987438 19.87305725142457
H 12.06801895192497 6.54798572133719 18.08960338592548
C 8.44057673943815 6.71640966340738 20.35763454498830
H 8.46711064603934 6.30166583381755 21.39111326889347
H 9.06333976744300 7.62841792558850 20.35771638150766

H 7.39697724096788 7.02464652613669 20.14936839457283

108

1_int-2

Mo 4.37115929125229 1.36976808838897 5.44911211652874
Mo 3.24804220459452 -2.09767942697680 6.27557321645156
N 4.41591872638780 -0.53419043358433 5.46578299900941
N 3.18563765881572 -0.21704966942336 6.05290305100087
N 3.47467444257078 1.75880776273391 3.54576717897282
N 5.66694164935570 1.50786061278411 3.70457516410086
N 4.16084233574940 -2.22909284562538 8.23049253105399
N 1.99475141626452 -2.02606291169005 8.01332085136586
C 4.60984143097610 1.51669163529433 2.87430702078005
C 4.68127338031022 1.21424203777149 1.39744766306068
C 2.13909830911218 1.64964050272738 2.96175978072595
C 1.11084569956988 2.32185622533239 3.87094749728902
C 1.75439288710733 0.18001696397304 2.74181540746543
C 6.95764809160515 0.94973259090506 3.29594293571858
C 8.12368488079409 1.75696088173331 3.86988833211273
C 7.05944204150457 -0.52807098164473 3.70314334420496
C 4.34516754693592 2.04399410368775 7.54515315803759
C 5.63218273848242 2.46140692497530 7.01497035813760
C 5.36663282328090 3.52336924465602 6.05964586552641
C 3.96627713991964 3.75486502224176 5.99462101452699
C 3.30290435956352 2.82016729305184 6.86643182118056
C 4.10775533174937 1.14624729942789 8.72181330790780
C 6.97220649386375 2.10769790488667 7.59240241533590
C 6.37615142672019 4.32269220470611 5.29175001585138
C 3.33621757473252 4.79589157457006 5.11856171019437
C 1.85626104065893 2.79961733723560 7.26426768376337
H 4.79354984015874 0.12332149906751 1.22669004283706
H 5.55197242463080 1.71326864290541 0.92993955784611
H 3.76501468591990 1.54778199372446 0.87783172907499
H 2.13148089056795 2.17882331865220 1.97884284467445
H 1.10396868723831 1.81562599026653 4.85538841146505
H 0.09701552410152 2.24994238217616 3.42860010493679
H 1.34783311605151 3.38856541739781 4.03239825288758
H 2.42412329990651 -0.31191867910778 2.01349866129509
H 0.71341419771969 0.08939118773534 2.36760530755318
H 1.84138972423496 -0.36121553278543 3.70624619896189
H 7.03214054987194 1.00550938624369 2.18676647742548
H 8.09824688867978 2.80740233217245 3.52307168611464
H 9.09220316806697 1.31139542794145 3.56442637059388
H 8.07894852867635 1.75611665692039 4.97647699052231
H 6.94375923200718 -0.62316095322452 4.80115261195393
H 8.03423144584563 -0.96193445046205 3.39826649540265
H 6.24792384009298 -1.12094796337578 3.24083546146133
H 3.91702521737840 1.73282085744334 9.64826013449990
H 4.98187929442054 0.49773662084679 8.90421417023310
H 3.24865409709388 0.47217180789812 8.54709987015457
H 6.98916652870371 1.06848778227344 7.97261348835038
H 7.23681015702455 2.77679438685082 8.44252236717533
H 7.78143683774696 2.19392511245780 6.84198340395787
H 6.24112678501347 4.20514533037550 4.19713216574087
H 7.41023227252980 4.02141433496796 5.53677574099603
H 6.27514542631009 5.40425029878916 5.52658604402951
H 3.79287870127006 5.79107340192919 5.30406108023917
H 2.24957197688177 4.88609479929341 5.30475413443975
H 3.47973268410094 4.56211573555462 4.04293590963197
H 1.23164586105703 3.40912492983881 6.58507852623574
H 1.72007676307483 3.19915849440596 8.29406750870133
H 1.45403293931026 1.76694992008685 7.25604929570251
C 2.98919646157681 -2.31100503383059 8.87254403083685
C 2.76565309005740 -2.73787756971851 10.30359492110921
C 5.50148459908907 -2.52371373530579 8.73407087637134
C 6.50562602446975 -1.91971277764997 7.74557865687088
C 5.79362580287275 -2.00008636822488 10.15323383101358
C 0.62479906690477 -1.70186712871807 8.39775836719018
C -0.18607229169231 -1.39059338621160 7.13818932296213
C 0.57106376348145 -0.49568999192447 9.35396546435193
C 3.36498959368531 -3.19270524810518 4.34257790828272
C 2.03665418760778 -3.46812449525652 4.86097404910799
C 2.20342028529728 -4.30364246349353 6.03505530846198
C 3.58618702095108 -4.52722254951884 6.25466363031836
C 4.33954645246387 -3.80450304790262 5.24931418635267
C 3.71231367778445 -2.60781925687042 3.00139833215786
C 0.74913318487134 -3.23411987097437 4.12288423956458
C 1.10613240475407 -4.87457545679620 6.88204824335118
C 4.14764739546403 -5.38288833061759 7.34940679804256
C 5.79788586543123 -3.97691809458148 4.93667903762641
H 3.01968145480771 -1.93574752919591 11.02486317816231
H 1.70731752784989 -3.01843726204289 10.46073168123116
H 3.39613827353444 -3.61528734305403 10.54612988921577
H 5.65874749986407 -3.62919598327743 8.74772144020226
H 6.42139131528355 -0.81511969405254 7.74207323044354
H 7.54439749294744 -2.20302400321436 8.00946773444053
H 6.28139295292558 -2.24732765308911 6.71711200969511
H 5.24860402181936 -2.54554466723616 10.94418058091531
H 6.87616938856476 -2.10937491352750 10.36755473428440
H 5.53925778791446 -0.92429742145304 10.23611382298098
H 0.15402989047045 -2.57732774366679 8.91261744896134
H -0.22553138718426 -2.25938604917644 6.45899811352482
H -1.22316694293605 -1.09998880604947 7.40048055479800
H 0.29577501062372 -0.56274110111236 6.57936261305061
H 0.98974961489467 0.39875457267493 8.85157181142264
H -0.47640608778925 -0.27341418024958 9.64523019171308
H 1.15064606347668 -0.66453317440602 10.28129765128425
H 4.41639493950661 -3.27185241674937 2.45607145603028
H 2.81136562090514 -2.49053608774838 2.37119643398868
H 4.19259654454506 -1.61241840669811 3.09574492918212
H 0.71193571276864 -2.23073034111603 3.65865599634786
H 0.61623572782403 -3.98376601607548 3.31033170218468
H -0.12944883275734 -3.32409301794499 4.78999992520782
H 1.38191825533284 -4.87586647910214 7.95502501796131
H 0.16471994298293 -4.30364763614322 6.77859793382996
H 0.89146863369590 -5.92647538284322 6.59282467561401
H 3.96372119498732 -6.45992056510561 7.14484411782211
H 5.24043076558421 -5.24856400453586 7.45572567347602
H 3.68150265883499 -5.15596065703144 8.33012616901699
H 6.37837919279227 -4.27719635089522 5.83006287712983
H 5.95265680875333 -4.76536175839963 4.16491240230312

H	6.24286318142237	-3.04182295939195	4.54828485669581

108

1_dia

Mo	4.34862296094893	1.16986654898850	5.45065640762959
Mo	3.63014093033337	-1.16908748530140	6.42255227738426
N	5.27883852127417	-0.44952820538659	5.80780415911132
N	2.69983108732210	0.45014648434351	6.06500928341759
N	3.27779353550205	1.96307858151471	3.65985392611803
N	5.34622174454001	1.24259043153760	3.45434099991720
N	4.70084092803295	-1.96212711376680	8.21343823285419
N	2.63241955855058	-1.24157103730551	8.41886588760371
C	4.32760437380278	1.85188195530134	2.83932641359944
C	4.37037563682627	2.37809235603346	1.42779725941539
C	1.97921000367137	2.49095960961426	3.24219673109388
C	1.08478650681359	2.73810230432782	4.45796370714241
C	1.25228401024302	1.55283434227688	2.25813842152459
C	6.59493066104869	0.88155785946503	2.78377939020167
C	7.64681851568741	0.45001247720650	3.80665519371266
C	6.40203460584267	-0.24318795896398	1.74716706719707
C	4.88852138625974	2.14564237353221	7.46463709947224
C	6.11170264816213	2.27713680786338	6.70463958677875
C	5.85189413376092	3.22013093704421	5.66161519965247
C	4.49783556137081	3.69232434619088	5.79478437530413
C	3.89826416840652	3.04937560873110	6.92264761761349
C	4.76142178908153	1.41340606603838	8.76280668499197
C	7.38531156297415	1.57402265054617	7.05303158955237
C	6.81459357775723	3.78545388587440	4.66276715010555
C	3.93340651567759	4.79018559343959	4.94617983617102
C	2.54795249724921	3.26301931459702	7.53001497901617
H	4.03061668152345	1.61255275850593	0.70075010001061
H	5.40118503795794	2.66674418326826	1.14536661787322
H	3.70793828962166	3.25784649541389	1.31441462053290
H	2.13438983241739	3.46787314217887	2.71909782164686
H	1.05005759825120	1.82219844018505	5.08106995699263
H	0.06063940558513	3.01418343672522	4.13619106265857
H	1.47561894215402	3.55108953063973	5.09146336489242
H	1.89076143050097	1.26204032784370	1.40198521777474
H	0.34564709434358	2.04877819534544	1.85332184363986
H	0.93003580661476	0.62826796745499	2.77622658642868
H	6.98659080777372	1.77603462263207	2.23725558694621
H	7.95516749947186	1.28980024128064	4.45050212311218
H	8.54857268146038	0.05876317129467	3.29441823655623
H	7.21923924408308	-0.33104079526416	4.46649377670303
H	6.17058050429907	-1.19917399353568	2.25678470264954
H	7.33265891476956	-0.38817630967807	1.16003635678169
H	5.58233716290802	-0.02609365273044	1.03567323634053
H	5.07679795170171	2.06500847155473	9.60792877354988
H	5.38449000665008	0.50510695710368	8.77272374866954
H	3.72330305457173	1.09108736024592	8.94084087679367
H	7.26948780195393	0.47937173506522	6.88601545427997
H	7.64873807006789	1.73080441140249	8.12011359752181
H	8.23658849004794	1.92872899942528	6.44245844408890
H	6.40883192828407	3.74265546924268	3.63242265425657
H	7.78268281251285	3.25259357865276	4.66668963947618
H	7.01925897115664	4.85480856517700	4.88810710604083
H	4.47448988771774	5.74225097805117	5.13845792828342
H	2.86215793398298	4.96798147147695	5.15084789084565
H	4.03952871730822	4.56913686891488	3.86547571726370
H	2.00692003266209	4.10304350722091	7.05573186285303
H	2.62894100362223	3.48530261129000	8.61477436473868
H	1.93208259415902	2.34284606590377	7.41383662359631
C	3.65099297466386	-1.85090795459343	9.03392861082761
C	3.60794042679533	-2.37762135808349	10.44526545573410
C	5.99889226119553	-2.49169476948982	8.63068617776893
C	6.89206617485061	-2.74112710921961	7.41447399974320
C	6.72803622017556	-1.55390762846330	9.61342391620026
C	1.38312477154314	-0.88179675452513	9.08901654787933
C	0.33068965491304	-0.45266269557180	8.06567689893737
C	1.57419563367247	0.24421294395948	10.12461657539942
C	3.09000685155336	-2.14514403699516	4.40869430427038
C	1.86707347527364	-2.27668125926392	5.16906818143580
C	2.12741131942725	-3.21942571918479	6.21221864751739
C	3.48145917774132	-3.69156895155242	6.07859166870138
C	4.08054393371653	-3.04872282055059	4.95044132454899
C	3.21673077457594	-1.41268923630873	3.11059673013700
C	0.59333873261260	-1.57361770929228	4.82094950647864
C	1.16529216753695	-3.78448542168991	7.21176122539889
C	4.04645244945939	-4.78917231362635	6.92715989184312
C	5.43086452064462	-3.26193738133207	4.34296664502972
H	3.94743019528538	-1.61230119758138	11.17265477154206
H	2.57706844756595	-2.66642241642037	10.72735557082424
H	4.27045917173348	-3.25734214795593	10.55843300391750
H	5.84246931472895	-3.46792014726458	9.15466427515601
H	6.92803658174894	-1.82560269654113	6.79090161931406
H	7.91591481146476	-3.01893159327663	7.73570880720432
H	6.49930248247445	-3.55365157477114	6.78155694074280
H	6.09059508258176	-1.26109541896875	10.46964888462417
H	7.63400910748701	-2.05108996817690	10.01821259881266
H	7.05166111922558	-0.63042927221833	9.09426801144581
H	0.99270963375293	-1.77636124769324	9.63631481345461
H	0.02355028400029	-1.29340624631766	7.42245551336832
H	-0.57165899470748	-0.06229283517732	8.57754018419722
H	0.75714554832800	0.32841716476898	7.40514223411258
H	1.80399798746585	1.20011938982745	9.61409808866121
H	0.64335359400418	0.38818804554582	10.71164901229305
H	2.39428713365700	0.02920045898934	10.83625970864462
H	2.90103807899154	-2.06406699129142	2.26542725060976
H	2.59374710011038	-0.50430882637123	3.10109957012248
H	4.25482647222956	-1.09043641212201	2.93231882140968
H	0.70924540927570	-0.47896382695216	4.98796579394504
H	0.32971226588753	-1.73037276167722	3.75391639601912
H	-0.25791918364276	-1.92824481540981	5.43161470479502
H	1.57335103164828	-3.74498291046317	8.24131601646648
H	0.19861524204111	-3.24909952171730	7.21123836671979
H	0.95728247118327	-4.85281163478918	6.98454312133640
H	3.50625584978821	-5.74167326015374	6.73450963211926
H	5.11790547747449	-4.96610562169499	6.72278396793599
H	3.93986047470890	-4.56842645344361	8.00787503385497
H	5.97198547511151	-4.10215355790846	4.81682823612420
H	5.34999981430785	-3.48352260462335	3.25806462376285

H	6.04655738846146	-2.34169246839759	4.45969874823286

108

1_dia (triplet)

Mo	4.33107836587147	1.09793964004400	5.44987743747897
Mo	3.56793163440024	-1.36461728791347	6.46300485611016
N	5.18130452729153	-0.48554769930851	5.72400325589547
N	2.74622862279048	0.36418023956920	5.96640656078735
N	3.27742127060847	1.94650932458279	3.61795090367031
N	5.35611476472022	1.24881527566391	3.43309708160700
N	4.71020409271532	-1.95271777011846	8.23779221426944
N	2.65131548697175	-1.22759636991026	8.44913637123212
C	4.34175030266456	1.85739995748595	2.81516485537843
C	4.39533348911908	2.39110793652080	1.40595974055714
C	1.98041852990737	2.46274808483018	3.19111316638031
C	1.09761369168641	2.73985185582738	4.40970555627471
C	1.25671196107978	1.49219474444979	2.23688326761082
C	6.60056976398598	0.87611554694491	2.76872519948381
C	7.66243613391329	0.50037989830328	3.80409940845414
C	6.40169377629785	-0.29293162376543	1.78540856036616
C	4.87403074901611	2.12451141828872	7.45997196964178
C	6.10996674393503	2.30479625458950	6.72667377652332
C	5.87386815937501	3.28956470587906	5.72534397293886
C	4.50962295475806	3.75644492579755	5.85699473324279
C	3.90030585623924	3.06253923369087	6.94092070139021
C	4.74595989934550	1.38334925559431	8.75468050069839
C	7.38834696157354	1.60563691703516	7.07765590863383
C	6.83924961609568	3.85657117764509	4.73036946140639
C	3.93350793477691	4.84875821023619	5.00916890421472
C	2.54151126130824	3.26132195774310	7.54034890678778
H	4.06141536465293	1.62658031165767	0.67461985193280
H	5.42686736719202	2.68340517906699	1.13019471159658
H	3.73217459960970	3.26998140374621	1.28942480880011
H	2.12592594255532	3.42814269092611	2.64352010123681
H	1.02687313818700	1.82506053967953	5.03111952618674
H	0.08235912007118	3.05416421689971	4.09432715984749
H	1.52580705135263	3.53587885760189	5.04172405635277
H	1.88736531058174	1.21356790538199	1.37027961836001
H	0.32400390161497	1.95114257248969	1.84709900098269
H	0.98524394401409	0.56193400114736	2.77294187759974
H	6.98467305272271	1.74857184480350	2.18192039838044
H	7.93074833538796	1.36432369682733	4.43481761702755
H	8.58284728534363	0.13563211670958	3.30530244575512
H	7.26456609622047	-0.28972180551323	4.47160817183517
H	6.13769469663689	-1.21284878374736	2.34350843983581
H	7.33458750678236	-0.49098998743622	1.21731454293642
H	5.59372362228646	-0.09067616260568	1.05567713380177
H	5.03841217816987	2.03272288645413	9.61000027060180
H	5.38278771024553	0.48410376730885	8.76554274235077
H	3.71276478966787	1.03634068113039	8.91656896964447
H	7.28724172741540	0.50888898351149	6.93444433072582
H	7.65728085664029	1.78154398019299	8.14109080062086
H	8.23545644603139	1.95393729197023	6.45733782486010
H	6.49007802264355	3.69607978987770	3.68909522968753
H	7.84457886005224	3.40402108336663	4.82120733424813
H	6.95192457042610	4.95348950420326	4.86985378361563
H	4.53044275515744	5.78138375647734	5.10595513387070
H	2.89156391315681	5.08913774740040	5.29246285893706
H	3.93540150985771	4.57297396254908	3.93408707859541
H	1.99215474823575	4.09065608720777	7.05620907771331
H	2.61558092405137	3.49539719292332	8.62376939124188
H	1.93088710779022	2.33899842175173	7.43735654671770
C	3.66967275289347	-1.83549520679092	9.07572146466106
C	3.62825597997695	-2.36390380091848	10.48496127663909
C	6.00638279049526	-2.50656453110119	8.62704850959848
C	6.88697578537450	-2.70490099128826	7.39277055472761
C	6.74409433137649	-1.60871410727507	9.63950619809055
C	1.38519550510754	-0.89055790274805	9.09906752760420
C	0.36674838152064	-0.42347307885673	8.05862001280971
C	1.55211766417588	0.20202523450619	10.17340485433830
C	3.02422983571467	-2.27484808303599	4.34356068720284
C	1.82912230020632	-2.37484550543133	5.14125414884914
C	2.09358345009875	-3.29973052719413	6.20904213312409
C	3.44418395635607	-3.77326177532419	6.06699098456791
C	4.02149540851942	-3.14731405679641	4.90784914167284
C	3.14012725303315	-1.48786290742106	3.07808777349545
C	0.54627333453754	-1.68189212359561	4.79509002145253
C	1.13265830316019	-3.83425162148107	7.22836604728960
C	4.03587146013115	-4.85102300101211	6.92534869762180
C	5.37234140588251	-3.38600070337307	4.30194522603971
H	3.96646910967396	-1.60542448055340	11.22131993630179
H	2.59745368237316	-2.65680150778804	10.76417879119764
H	4.28909641346845	-3.24580117860420	10.59344162033827
H	5.85214819176490	-3.50416097586442	9.11008892308806
H	6.96218689230173	-1.75267947452926	6.83149902174514
H	7.89921839161569	-3.04661337199759	7.68761278420707
H	6.45759499595200	-3.45058796419514	6.70366114661789
H	6.11955633796110	-1.36065983565870	10.51872520818244
H	7.66081971239067	-2.11579712702924	10.00562933665463
H	7.05021828705048	-0.65895490824990	9.15802258071621
H	0.97546394679357	-1.80100844067333	9.60456553607629
H	0.11474302573463	-1.22751217489554	7.34795603689873
H	-0.56796972837628	-0.08916167728517	8.55140144614368
H	0.79462083950256	0.40848038951125	7.46534560016517
H	1.81145074734842	1.16968105550336	9.70021241300604
H	0.60253215520843	0.33948111598956	10.73101718167106
H	2.34377838053129	-0.04278607697159	10.90684149988575
H	2.41361702386230	-1.84856153088312	2.31846050095865
H	2.93671669701804	-0.41363445034387	3.25457813818754
H	4.15342191363206	-1.55916168057475	2.65074904995906
H	0.66205216161032	-0.58255904360731	4.90237540458360
H	0.26280805098257	-1.89094109405678	3.74201921733431
H	-0.29218032675272	-2.00955614385420	5.43781394302140
H	1.58139066417179	-3.85153354731512	8.24117481317407
H	0.20554380382073	-3.23503273817588	7.28124537197010
H	0.84372817245744	-4.87847830467008	6.97918906130734
H	3.57034496603201	-5.83315619017248	6.69134729332105
H	5.12570983202502	-4.95360898046228	6.77339588056679
H	3.86288456506372	-4.65454585934975	8.00181848053566
H	5.91338190146441	-4.20530761537181	4.81136379588491
H	5.27926047906940	-3.66419590066660	3.23152129669747

H 5.99286866021687 -2.46729380120315 4.36436677165667

110
2_lin
Mo 4.33382638155787 5.19841744651103 4.83390826855993
Mo 5.43462910149190 0.70478369707964 6.33296656715180
N 4.73716032372857 3.52684977748941 5.39395770257672
N 5.03223174525815 2.37613485194027 5.77151254365782
C 3.58471038599637 4.72569247247484 2.42893370107656
C 3.20508489923168 4.06325649911577 1.14759508297065
C 2.25931386131253 4.67544595406853 0.29663224643268
H 1.83101952610636 5.64980255463762 0.57740875294306
C 1.87898862042222 4.05247107210909 -0.90268034840973
H 1.14457493189372 4.53895490969911 -1.56281209662293
C 2.43594097907157 2.81204067560385 -1.26096986513719
H 2.13466996433557 2.32309069217931 -2.20007067569990
C 3.37995049009594 2.19905279773276 -0.41853531324195
H 3.81858501173981 1.22760442617741 -0.69342774182431
C 3.76517461608679 2.82265263754027 0.77834564951666
H 4.49570644116511 2.34211442991179 1.44301553738245
N 2.73103223628338 4.95379578782780 3.44123846819168
C 1.41317559760691 4.35601187585254 3.55594544406767
H 0.89351908589941 4.36117045920892 2.57229297415463
H 0.81588282922603 5.00165796394936 4.23465913089141
C 1.46370503326151 2.92919948674297 4.11407295917968
H 1.99322151401760 2.25655430018616 3.40864415261243
H 0.44380520366634 2.52496273061264 4.28005412375890
H 2.02362212925134 2.91355366369388 5.07124908947478
N 4.81413413095713 5.13513357201494 2.75942587691662
C 6.01225710483371 4.97083198738268 1.96599172011860
H 6.56267330976020 5.93845475526820 1.95808414222587
H 5.74889986875890 4.74644256857956 0.90850635233219
C 6.92584470514370 3.88294923733504 2.53980819424919
H 7.14582200526670 4.10025164717234 3.60453469331122
H 7.87843470883835 3.81913185820522 1.97489477245040
H 6.43092035596896 2.89083455904361 2.51180131341077
C 3.42062656043890 7.43297614519810 5.09824127233380
C 4.77150279603941 7.58253872045570 4.67736538533617
C 5.64227262682811 6.88193026665121 5.59993970365488
C 4.77433769987950 6.31964991238960 6.63747810947386
C 3.38063748923383 6.62887072699848 6.30412540651085
C 2.21592413392698 7.96116765130009 4.37902153327092
H 1.33299359639039 7.31474653260319 4.54729841368276
H 2.38567209826801 8.01637173407806 3.28611473020060
H 1.95378521575274 8.98255608330689 4.73190846078868
C 5.22720460266973 8.29442893559373 3.43897871246420
H 4.48082319790135 8.21536573334756 2.62443110359915
H 6.18071024182459 7.87562210297925 3.06274260078506
H 5.39567497786880 9.37535176402343 3.63679839513821
C 7.14244991818824 6.95051175282117 5.63209345299930
H 7.50550133030308 7.86319968042421 6.15949710196134
H 7.56851103788488 6.96741359840843 4.60916807188057
H 7.57265975070996 6.07345958570540 6.15343358622207
C 5.23272444042463 5.63698854767723 7.89510386257006
H 6.17339636983604 5.07665080299022 7.73338718688991
H 4.47547085219539 4.90549687405449 8.23943338592261
H 5.40479256536823 6.36112259675378 8.72193477237658
C 2.18292447868239 6.41231763190038 7.18461634454335
H 2.02095156227387 7.26535108563738 7.88425002802952
H 2.29449648096684 5.49733154377612 7.79831953425602
H 1.25771291071603 6.29564959327040 6.58596320444081
C 6.18299628154272 1.17848992247051 8.73829378901072
C 6.56272895997922 1.83994853030055 10.02008644485299
C 7.50983277770078 1.22784244956261 10.86959295336739
H 7.93911176984569 0.25438879367850 10.58724278073758
C 7.89038301518070 1.84977136747467 12.06937303904408
H 8.62607606825710 1.36343403942722 12.72817344433317
C 7.33215326529863 3.08900465095403 12.42977759008695
H 7.63377833049464 3.57726571119133 13.36914129329214
C 6.38656391387836 3.70175991602126 11.58900145899031
H 5.94699290592185 4.67232002862908 11.86544353931674
C 6.00127954500022 3.07928672664454 10.39155306964164
H 5.26939457798803 3.55970887375527 9.72835014181977
N 7.03686958807807 0.94985668346653 7.72619113871838
C 8.35491597498071 1.54713163558812 7.61128688279063
H 8.87545615672886 1.54045339198329 8.59445373040176
H 8.95118682112712 0.90198371284778 6.93125977150080
C 8.30482923081717 2.97464283245744 7.05499342959219
H 7.77656250135999 3.64672121307515 7.76185163507746
H 9.32482637042780 3.37839786653670 6.88854214018520
H 7.74401150140418 2.99187118169217 6.09838562331706
N 4.95348587687479 0.77002762984153 8.40710572176821
C 3.75494971003927 0.93357797281294 9.19998228369672
H 3.20464128921657 -0.03414304425400 9.20671892539758
H 4.01765501915592 1.15708822297964 10.25782805401738
C 2.84175735069618 2.02199917572820 8.62652255543847
H 2.62316462499813 1.80611642061849 7.56121433229109
H 1.88845455616375 2.08490137527786 9.19034502648522
H 3.33643728445838 3.01418414377548 8.65647523454030
C 6.34640867797825 -1.53071180252135 6.07171334469654
C 4.99523975060211 -1.67884519202019 6.49213837224347
C 4.12538035918363 -0.97874328104543 5.56832638808838
C 4.99412455557316 -0.41832898891150 4.53048746087978
C 6.38747939407428 -0.72807069018646 4.86487867397544
C 7.55053423310034 -2.05882996207645 6.79197466249948
H 8.43376291121485 -1.41281960636424 6.62370766636164
H 7.38021716923792 -2.11314542083499 7.88483114779186
H 7.81250432646236 -3.08056651781329 6.43997656060216
C 4.53825309473597 -2.38882275894681 7.73112213228957
H 5.28521411745863 -2.31108784776593 8.54525633968147
H 3.58604540778113 -1.96725805628124 8.10758618232976
H 4.36700028960852 -3.46944676073728 7.53399427900128
C 2.62518276316197 -1.04642502462620 5.53564486136501
H 2.26180489572131 -1.95945900105926 5.00905023332292
H 2.19871686049104 -1.06208609955550 6.55842593287064
H 2.19572436622920 -0.16968827599145 5.01314149502038
C 4.53664109178019 0.26279047664713 3.27169197306495
H 3.59747346008173 0.82586546341762 3.43277666435019
H 5.29564532812392 0.99138385277794 2.92507032194770
H 4.36209369922277 -0.46280120099344 2.44665351692680
C 7.58572432871645 -0.51363422007600 3.98459587955416

H	7.74743784617324	-1.36783783773674	3.28633012278434
H	7.47504870299704	0.40049414515113	3.36945040820938
H	8.51074706986918	-0.39669976667806	4.58348357242116

110

2_int-1

Mo	4.66809276532721	4.96790456857400	4.70213534917236
Mo	5.52064732503331	1.71746645337288	6.41201209151860
N	5.66500006889713	3.48591652725906	5.20951086549258
N	6.76780219303902	2.85075868996259	5.47218867667458
C	3.44018057820847	4.61332505532420	2.39559789684538
C	2.79470574835491	4.50653726559070	1.05788026622013
C	1.94558470318895	5.53249162607603	0.58689929569712
H	1.77409762875578	6.42009494201680	1.21542467232187
C	1.33567433389847	5.42758771144150	-0.67278961804963
H	0.67849463061794	6.23466588428751	-1.03168805197609
C	1.56727918004980	4.29709714539095	-1.47704157564050
H	1.08905037685188	4.21563497166134	-2.46518680547062
C	2.41059306996979	3.27124288521210	-1.01509955695857
H	2.59208092912260	2.38295400712921	-1.63957909178178
C	3.01983106221309	3.37358376845650	0.24574279259323
H	3.66881543603038	2.56592164157820	0.61688327959129
N	2.79708849226083	4.67038006904879	3.57670853965928
C	1.37678743056791	4.38390417625108	3.70910359358868
H	1.24292507255477	3.29243979363802	3.89904763755376
H	0.84620115489364	4.58540036782960	2.75321106728859
C	0.71530296589677	5.16537412678663	4.84049738160511
H	1.33362628522004	5.10764298201861	5.75943829033239
H	-0.28871676259699	4.75193335509150	5.06553706080509
H	0.59964045679455	6.23414451103657	4.58079766729839
N	4.75470183205642	4.66195950742386	2.61084441853805
C	5.80204047635559	4.59930853004440	1.61933018762773
H	6.42806232021387	5.51553399500184	1.69213167434082
H	5.36898676756666	4.59733913119774	0.59504381956902
C	6.69022835789689	3.37133151267911	1.83926438487058
H	7.06852946517038	3.35352160786465	2.88118872839214
H	7.55202290555111	3.37339873501608	1.14045823467499
H	6.11419281543127	2.43650754217148	1.68206524295899
C	3.99897403330759	7.28501250049251	5.17680007275383
C	5.02757913412656	7.21865797223809	4.18653767600275
C	6.19826412756014	6.55431097135321	4.75525152996309
C	5.83001761154224	6.20750642314782	6.12175074929032
C	4.45186807698018	6.62764549987356	6.36583237101784
C	2.68634287279709	7.97812793268092	4.98051343769000
H	1.95169558511126	7.70395648267787	5.75941919133066
H	2.24549951120553	7.74534230983414	3.99112641444802
H	2.82800064262839	9.08041649815682	5.02373487947565
C	4.91608346668376	7.80440655808127	2.80874296436379
H	3.96136157226069	7.52565478123509	2.31828105702031
H	5.73664700340850	7.45945389562110	2.15269071581133
H	4.95972241327008	8.91494894898531	2.83988174705159
C	7.59373742558637	6.52462115430257	4.20021039183296
H	8.19998659761945	7.38706825766739	4.56174355092624
H	7.59272023949728	6.56556682628917	3.09375165409464
H	8.11755451441871	5.59495081024722	4.49774319051490
C	6.78479325522767	5.66183632121567	7.14281736015180
H	7.40365731845970	4.84265912376579	6.72224715989889
H	6.23992982333285	5.24610501305354	8.00994739409333
H	7.46525099508262	6.45517864493696	7.52267637990511
C	3.72447721089435	6.55686328661680	7.67674767331676
H	3.95401608208249	7.43419644982332	8.32445488333507
H	4.00470747859892	5.64907630210443	8.24434526566395
H	2.62692871602327	6.52982467297249	7.52808927925728
C	6.98464413999395	1.73649265235292	8.51067978364306
C	7.99476644568113	2.09180466709964	9.54784369184931
C	8.42620479383777	1.12874168797340	10.48510995254234
H	7.99865558330881	0.11490964656669	10.45482719466689
C	9.37631018905046	1.46868854812905	11.46090880179872
H	9.70433313872863	0.71357593929219	12.19170220176912
C	9.90441394300629	2.77114766651988	11.50766213713163
H	10.64839951844582	3.03709794608558	12.27413641137738
C	9.48138144239117	3.73229805900585	10.57282647002902
H	9.89665175701968	4.75132093706309	10.60180192792572
C	8.53138853444330	3.39532540015101	9.59580431205119
H	8.20633139734371	4.13854933988048	8.85444048990381
N	7.20664066229735	0.89895568697718	7.49756107236912
C	8.49748633461658	0.31495918730346	7.18698890674323
H	9.02632208551038	0.03259284130326	8.12457895010395
H	8.30774993295631	-0.63193805126645	6.63707102530805
C	9.38796519945518	1.22926373609683	6.33939688768185
H	9.62806133599956	2.15914134192109	6.89380623150257
H	10.33855255482562	0.71844169115021	6.07995886961950
H	8.86255786275723	1.52683291470886	5.41230256772246
N	5.75604959919799	2.28876271731433	8.41563382477332
C	5.15706947873730	3.17636354859872	9.39580823085587
H	5.72650562012530	4.13259852950825	9.46384258996536
H	4.15930565136459	3.44833067464653	8.99441177021713
C	5.01710884359851	2.59254206094190	10.80637212899735
H	6.00660138714575	2.38880247017014	11.25959334663155
H	4.48593722783791	3.31164352614270	11.46331825789430
H	4.44501997693142	1.64557430010934	10.79399945559040
C	4.68566619775291	-0.49687362508971	6.85939739216684
C	3.65221530208777	0.34946943916544	7.35026472392767
C	3.25930088797623	1.25806262420984	6.31130239398774
C	3.99261661018044	0.87952281376915	5.09718929638057
C	4.91773862518347	-0.19100541690443	5.45392257265417
C	5.39248802839364	-1.55298031307404	7.65340544915477
H	6.35540072421615	-1.83173799791054	7.18688307710908
H	5.61035450464439	-1.20791133686362	8.68351744582663
H	4.77576234664873	-2.47514961045591	7.72853725778633
C	3.09899606545639	0.32394784335976	8.73958092400747
H	3.84612544657865	-0.04172293421744	9.46948519091712
H	2.75673126770549	1.32518544685984	9.06469502238281
H	2.22140932023338	-0.35654235916733	8.79472697626827
C	2.14654316048992	2.26020414461918	6.39501373476331
H	1.18003236311334	1.83987360967569	6.03443245718229
H	1.99384406264948	2.60350297781559	7.43695255415532
H	2.38163907243590	3.15382474261753	5.79030402882394
C	3.71677946306463	1.39351289594493	3.71659512406990
H	3.51915450386034	2.48204937059004	3.73219672111000
H	4.58085612378208	1.23010757976747	3.04511578917511

```
H  2.83353468045040   0.88789978188342   3.26816401822868
C  5.76611451235383  -0.98621937468469   4.50226194894480
H  5.20782678980873  -1.85299807808198   4.08063864084533
H  6.10740396796820  -0.36341144823835   3.65278245573260
H  6.67087416353669  -1.38399859274254   5.00212331090941
```

110

2_int-2

```
Mo 5.76798307687678   6.92176813515382   3.69667552180014
Mo 6.84024625981861   7.10958354317505   7.26521765146056
N  6.98546466650840   6.92311076883168   5.37439131848915
N  5.60702843611857   7.06854237775526   5.58370869494905
C  4.54609770288916   9.15692675682246   3.10582758789371
C  3.84059462347700   10.43490136335063   2.80269855424366
C  3.93124392087226   11.54667160674085   3.66806779687211
H  4.52290266080207   11.46630522422431   4.59070987859712
C  3.25849703864864   12.74030403828080   3.36246485491314
H  3.33229367130951   13.59886543094350   4.04749226190778
C  2.48912810910746   12.83835769328924   2.18961497955407
H  1.96231816834725   13.77516013578037   1.95155219537033
C  2.39311739212953   11.73557226268445   1.32271887081999
H  1.79292722160496   11.80687593365838   0.40260949618856
C  3.06378167115767   10.54063663363089   1.62773809820081
H  2.99572826088013   9.67620113206729   0.94949942797793
N  3.93705692465975   7.97809701562124   3.29467459022227
C  2.52453538228135   7.79073456582596   3.54411294391171
H  2.18104019781973   6.90653471189094   2.96393631795870
H  1.94111902860515   8.66030328593100   3.16833513510800
C  2.23308722686580   7.56266037222343   5.02929100819350
H  2.87085925817200   6.74320426764812   5.41733654810743
H  1.16706958871004   7.30376474654778   5.19691944014239
H  2.46754381679681   8.47454371932026   5.61467625962271
N  5.87101941411591   9.00857229336491   3.21914296670386
C  6.86785873907869   10.00971449767075   2.89843469535427
H  6.50631535895907   11.02284264601584   3.18180959577003
H  7.04683846042256   10.04787736770478   1.79725871845961
C  8.17851090945244   9.69846238691033   3.61547761608662
H  8.02088010463923   9.64147595778646   4.71043682973331
H  8.94616334915453   10.46777083832677   3.39662962496455
H  8.56280595999924   8.70789645969689   3.30787579540266
C  5.15016781453914   5.89716284709667   1.58837991488070
C  6.43543739459390   6.46953793648390   1.39007745939645
C  7.33869660789805   5.92952953539299   2.37728239806156
C  6.59005366291467   4.94552928182962   3.16661423653279
C  5.21120455294230   4.96264248768650   2.70157588354267
C  3.93895814019483   6.20242425056708   0.76094380033897
H  3.97562444013868   5.65799410296286  -0.20767005781342
H  3.86348332306774   7.28416629759569   0.53244166707194
H  3.00648526608158   5.90190375397867   1.27418515196373
C  6.76707515927120   7.50464733091246   0.35775670543481
H  5.95024532727833   8.24657243779410   0.25071801003002
H  6.92304763098794   7.03996729194504  -0.63995922335545
H  7.69200251756221   8.05510368463971   0.61547868396637
C  8.82483807292672   6.12287338052727   2.43309613548920
H  9.13367015099801   7.05769781088873   1.92591073689979
H  9.36138435027975   5.28438657949991   1.93427978104773
H  9.18150769188411   6.17396394300560   3.47806385945271
C  7.20011428732576   4.00148128556380   4.16235875722257
H  6.41723087972404   3.51788000422594   4.77581316900969
H  7.87151401635245   4.53762081881479   4.86207267826999
H  7.78365794650938   3.19784677933199   3.66117733395862
C  4.11239940349156   4.02648414334506   3.11735169462238
H  4.24066465911036   3.69897026969590   4.16607954708412
H  4.08486746174588   3.11330999745349   2.48013606792797
H  3.11864165992395   4.51084037742320   3.04585069071870
C  7.92570433542863   4.83426101306678   7.94140424742900
C  8.53310121237697   3.50772641450722   8.22855986728284
C  8.20403216778166   2.38324756044266   7.43890909326278
H  7.50422642580363   2.50332093520916   6.59936216143075
C  8.77727733118585   1.13169481658189   7.71110816072179
H  8.51727770370440   0.26406045124422   7.08539815489136
C  9.68489596043912   0.98676019848044   8.77563855446322
H  10.13437382387193   0.00463947547315   8.98823220898190
C  10.01566497569122   2.10009902678088   9.56879162793419
H  10.72050486925735   1.99016552004739   10.40729699763773
C  9.44423635007582   3.35303076406795   9.29763471849458
H  9.68906640441480   4.22266837238085   9.92636913721142
N  8.60867389542184   5.97371896568621   7.73874845652638
C  10.02567699211144   6.07166344917721   7.45554954374697
H  10.44096101805439   6.91810711741279   8.04496216503637
H  10.55974857172067   5.15792521106875   7.79690643827437
C  10.29693333396211   6.31079010410508   5.96873250982303
H  9.70880450941215   7.18018097008569   5.61200313007968
H  11.37455364689892   6.49761167905172   5.78013215140556
H  9.98490038070517   5.43004386058864   5.37246861572305
N  6.61346114913557   5.07014215694373   7.81867458599241
C  5.52981355026589   4.19720960254624   8.20765622307053
H  5.92156589803911   3.22460946373930   8.57965330666532
H  4.98651352147463   4.66006015431382   9.06265278337863
C  4.54384351142264   3.98521937724464   7.05788266843326
H  5.00320348420490   3.38161175137708   6.24993647199781
H  3.62811090823712   3.46427708035132   7.40438220970577
H  4.26455565328512   4.96422087266499   6.61788267958566
C  7.53017472896009   8.27683804083643   9.28456114626131
C  6.26729440642499   7.68416808879856   9.55380277302803
C  5.31980110884629   8.14255593260782   8.56020795164664
C  6.02788262965324   9.10548924567655   7.70165837779967
C  7.41563517209013   9.14926455135529   8.13289073412706
C  8.77389534885804   8.03532996065678   10.08394045633184
H  8.74731734587806   8.61042729068359   11.03489620086430
H  8.88685671213671   6.96390323632314   10.34351463582745
H  9.68090401626980   8.34763675901254   9.53277130499719
C  6.00293353134195   6.71081477399117   10.66306089450797
H  6.78126149414425   5.92125083613213   10.70681863200008
H  5.99687119429892   7.22147390410632   11.65019573054508
H  5.02220781024886   6.21227725103532   10.54422533167675
C  3.84283188391088   7.87871987135835   8.54486255929959
H  3.60460746643557   6.89166724795983   8.98793567782598
H  3.27635624382671   8.64878449161997   9.11631869254773
H  3.45584652722739   7.87460497335646   7.50949317779627
C  5.38934844062711   9.97944270406505   6.66113334901496
```

H 6.11981442087958 10.26674749785389 5.88115223338326
H 4.57211033556643 9.44224378889231 6.14346017352229
H 4.97340392772314 10.91105734113106 7.10619251968473
C 8.47796597786491 10.09090933318944 7.64326462684994
H 8.29353844212242 10.39456049111950 6.59528226606050
H 8.51811103592102 11.02017238750700 8.25608884171334
H 9.48302353057762 9.62570173836820 7.67743954219828

110
2_dia
Mo 5.81925179449806 7.17539005096579 3.78078469028758
Mo 6.49406728998093 6.92003585751285 6.30036871194275
N 7.38705836282092 7.63036264887980 4.79196066480533
N 4.92631645031170 6.46503603334263 5.28913291069606
C 4.54657586225503 9.36822379776064 2.91055199314475
C 3.81747570754976 10.59395936693577 2.47431647479467
C 3.86942111790101 11.78294549874853 3.23306906095804
H 4.46018511476912 11.81094187435236 4.15959222271513
C 3.16005432094235 12.92046603468136 2.81675872326630
H 3.20332300433602 13.84102991250558 3.41877044051843
C 2.39890342542146 12.88599227171770 1.63521570023660
H 1.84483225051947 13.77954182352154 1.30922691966068
C 2.35107007119414 11.70835305186163 0.86878477888259
H 1.76311829391410 11.67762467398847 -0.06129560077193
C 3.05506440927714 10.56813923268495 1.28659274583825
H 3.02421007588187 9.64793657849346 0.68365175628873
N 3.95734025881417 8.22910169929230 3.26980824965522
C 2.53352983595373 7.98033520247933 3.36400049307998
H 2.41387612992774 6.88809092884652 3.51217533527626
H 2.02098689629324 8.22714708134185 2.40696520060876
C 1.84071655446768 8.71914197730025 4.51383904202978
H 2.27820077219865 8.41950277936777 5.48414232682733
H 0.75719609228108 8.47935904206540 4.53231341072296
H 1.94616819682540 9.81738428727374 4.40721678973342
N 5.87488146338860 9.24743338671741 2.98466185291513
C 6.80448974534520 10.35827943079588 2.93116420383094
H 6.85356880515960 10.85692022846768 3.93109299065916
H 6.43556757079584 11.13518561506464 2.22342893512862
C 8.21580047734202 9.91426079216983 2.56123657381953
H 8.53566610127014 9.11231060344468 3.25489411847540
H 8.92182726381098 10.76558570993509 2.64027327789903
H 8.26043155849545 9.52320321742283 1.52739321810805
C 5.04163876141148 6.12934962412822 1.60045322323126
C 6.31488986784054 6.73877373161228 1.32004841167563
C 7.29994016105400 6.14806020847518 2.17131963848649
C 6.61214983130146 5.18097693170043 3.00572436126492
C 5.21192667310783 5.15412471954947 2.63127058474839
C 3.79681361536622 6.40908701847411 0.81815773601052
H 3.84840315684322 5.91334466198863 -0.17608163790201
H 3.66649643463961 7.49323712580029 0.63439131095731
H 2.89013717283981 6.03866667125381 1.32981149027424
C 6.52434694024458 7.74850287190081 0.23327910611556
H 5.92228899115310 8.66426379089422 0.40386587704648
H 6.22373789472031 7.32972116255303 -0.75094574779854
H 7.58419441372146 8.05479476293277 0.15847165697942
C 8.77081384216675 6.41307282539362 2.21404066517496
H 9.08411134253512 7.11438304575960 1.41819637041893
H 9.35733693915412 5.47771370590722 2.09693010299810
H 9.03943904009980 6.86828543705745 3.19493360823983
C 7.28509874756788 4.17858372303988 3.89267471642607
H 6.57475808656611 3.77034681322093 4.63298348477032
H 8.12982670460839 4.62401852176513 4.44542001235161
H 7.67721451889243 3.33160232766720 3.28712426459973
C 4.17814627952718 4.25391051946106 3.23211543774141
H 4.08749508387636 4.46822095675302 4.32176749290303
H 4.43891648948906 3.18184480022012 3.10709482632913
H 3.18387743033309 4.41476330174859 2.77222766884276
C 7.76692154288250 4.72731372973819 7.17057057713914
C 8.49620541897507 3.50162303757162 7.60666059806057
C 8.44527508852862 2.31303978183402 6.84719377496220
H 7.85483123349360 2.28519646671299 5.92046012611271
C 9.15496773429259 1.17564154775021 7.26332242106874
H 9.11239449647460 0.25533884919005 6.66086165873333
C 9.91562938810752 1.20990633253041 8.44519629210563
H 10.46992924125848 0.31644003318837 8.77102083709953
C 9.96266414399268 2.38722367266344 9.21216700002222
H 10.55014613645187 2.41777012329112 10.14254800477705
C 9.25817149388866 3.52725165144650 8.79467184498722
H 9.28844379879285 4.44722567955602 9.39800068295599
N 8.35604838546250 5.86646002877235 6.81123620995051
C 9.77980057380462 6.11508574861198 6.71622203459073
H 9.89954204499119 7.20728079437981 6.56780511422031
H 10.29288344237264 5.86842947461621 7.67301816557960
C 10.47187225259372 5.37592724629527 5.56617163522653
H 10.03372360040865 5.67528347567399 4.59607454107845
H 11.55537420419172 5.61572825617093 5.54690949192282
H 10.36652887627843 4.27770703577595 5.67319124764854
N 6.43860933051931 4.84788592336360 7.09634006287932
C 5.50920780947687 3.73685204106401 7.15000603659019
H 5.46064732165545 3.23763393745862 6.15033989480400
H 5.87794855743968 2.96040239292533 7.85833678614703
C 4.09766734216204 4.18092587031416 7.51903757457671
H 3.77810811737266 4.98258237416844 6.82487614088487
H 3.39173499038536 3.32951668756534 7.44006822255585
H 4.05246452546819 4.57248104735360 8.55266804528936
C 7.27127151382591 7.96568923493588 8.48093970989669
C 5.99789576972827 7.35640616184952 8.76100361699490
C 5.01311706610942 7.94730943326406 7.90954885174454
C 5.70123671053846 8.91444719586423 7.07544061039035
C 7.10137530567952 8.94102533579645 7.45015541376026
C 8.51592080648745 7.68580658109126 9.26343672682057
H 8.46408145838383 8.18129285193107 10.25779184103690
H 8.64627496074590 6.60162247770789 9.44697413376040
H 9.42257624399398 8.05649776650973 8.75196902727410
C 5.78808117710770 6.34662934065044 9.84765954770956
H 6.39005636461444 5.43080177541965 9.67714184122213
H 6.08855745853722 6.76531455737140 10.83196599641725
H 4.72817494738192 6.04048059322242 9.92224909585461
C 3.54223463819232 7.68239743259286 7.86644237754302
H 3.22869607498960 6.98108376445541 8.66219723882846
H 2.95575573289036 8.61780275230917 7.98340439278434

```
H  3.27385275965496    7.22720795960581    6.88546423709783
C  5.02863128606749    9.91716215995522    6.18858407873448
H  5.73945300163705    10.32604413646014   5.44906514988668
H  4.18442853798738    9.47185145101051    5.63496153423717
H  4.63586873242505    10.76366829589156   6.79437807790764
C  8.13548787312669    9.84100602790558    6.84953426362730
H  8.22558091256600    9.62713213270952    5.75975929830442
H  7.87529224411626    10.91314400245919   6.97515534860753
H  9.12979763848822    9.67923738985323    7.30905069367052
```

110

2_dia (triplet)

```
Mo 5.80952272742269    7.19382225732527    3.85738209593826
Mo 6.53375557506906    6.89539012890745    6.50547747790726
N  7.29668058329885    7.62035280475963    4.84467113489148
N  4.94137347446379    6.60242182014321    5.33421402707950
C  4.52147307407372    9.39339298975644    2.90093610856011
C  3.82722324959837    10.61580354441153   2.40227163901392
C  3.74121460592880    11.77508516445051   3.20290553457125
H  4.20178000718551    11.77850526675602   4.20162145014368
C  3.06806373598523    12.91361345514090   2.73228291778236
H  3.00513878060020    13.81166119328065   3.36587831883092
C  2.47749600968192    12.90852065043184   1.45621935101450
H  1.95151196481294    13.80256160568724   1.08774638097003
C  2.56417598995377    11.75952920881751   0.65070668352404
H  2.10882294319594    11.75168996125989   -0.35150344140709
C  3.23463492722537    10.61906956535189   1.12105698627686
H  3.30991114188460    9.72060982379761    0.48954074667121
N  3.90531604353407    8.26390161424586    3.22440068071426
C  2.47423358359189    8.05555522787523    3.24692203969177
H  2.29959434538325    6.96076593311989    3.20353954675049
H  1.98829990579330    8.48634492791172    2.34153405214898
C  1.80366339193342    8.61782588014703    4.50536536038560
H  2.24996664538985    8.16320901184899    5.41044994173940
H  0.71442580721816    8.40369214990343    4.50431471060630
H  1.93956849084542    9.71653972218867    4.56805775246617
N  5.84238591004524    9.28606935552464    3.08042116101170
C  6.77019359179795    10.39618859302557   3.01308759398408
H  6.81055754659117    10.91670983493072   4.00293060312156
H  6.40860480803556    11.15906623888360   2.28656837366346
C  8.18384578544272    9.94581773292524    2.65454299137692
H  8.51683410528160    9.16698564087166    3.36816049697777
H  8.88835880416106    10.80107013181757   2.69882129863523
H  8.22119540792554    9.51708101769857    1.63492791839635
C  5.02940136923923    6.08861259437105    1.57469333157141
C  6.30709138519009    6.70851354908146    1.30178255473460
C  7.27921674036180    6.13194528598567    2.16857394865561
C  6.59436121003298    5.18022853814338    3.02188736897853
C  5.20078906784359    5.14175956643642    2.62479502472115
C  3.79126461303070    6.35506812199129    0.77713461203830
H  3.87638737347551    5.90519422775309    -0.23659619386400
H  3.62211945977771    7.44108893936828    0.63763736776481
H  2.88845744002418    5.93084781301661    1.25406083872180
C  6.51494484093862    7.73871294517660    0.23428049570484
H  5.94408240585615    8.66651556169879    0.45011060918258
H  6.17317758460276    7.36283899565546    -0.75363170320647
H  7.58088561309835    8.01906964197423    0.13717592818485
C  8.75008224213492    6.40609833621859    2.22019567853618
H  9.06014841295363    7.12774004504164    1.44122130918005
H  9.33982148305362    5.47564406142958    2.07883263837918
H  9.02326725157906    6.83503753962070    3.20909838693038
C  7.27171307033881    4.17590407772222    3.90509482650339
H  6.55485018381859    3.72897674113200    4.61572205328633
H  8.08425203684458    4.63301217517343    4.49527136981557
H  7.70818409165146    3.35489614414038    3.29437629240183
C  4.16549883448030    4.23593371825360    3.22163339132346
H  4.14978247996956    4.34301967137824    4.32636308523582
H  4.36436259132282    3.16914188580321    2.98233700292154
H  3.15128543918885    4.47927342145887    2.84970969222896
C  7.79602912769281    4.66359917209239    7.15031027866477
C  8.51221061596574    3.43846009132769    7.57774675724281
C  8.31639453881327    2.20357976937202    6.91546545188660
H  7.62007459370271    2.14741784745185    6.06690302894107
C  9.02517937139306    1.06086042830149    7.31520631421476
H  8.86745586175854    0.10898291108926    6.78497369905285
C  9.93664265351998    1.12902512353213    8.38444510326399
H  10.49191644626889   0.23126942070306    8.69633030996844
C  10.13296975341196   2.35037828646033    9.05474355942482
H  10.83740829793718   2.40983260056764    9.89872479952841
C  9.42786564477194    3.49489277270493    8.65556588710784
H  9.57067963692323    4.44419682521528    9.19286502403999
N  8.37657335059013    5.83098587487331    6.83816785092645
C  9.79409167228537    6.09976448847747    6.70837617141409
H  9.88854880525958    7.18623992370410    6.50652554178757
H  10.33461118307424   5.91241394416556    7.66379542817419
C  10.48065357471668   5.32223497366829    5.58013585398563
H  10.02617841867377   5.57337378526803    4.60364108399457
H  11.56016699305830   5.57615463493598    5.53613958956579
H  10.39247476208529   4.22822805003003    5.73357492038035
N  6.46486453935853    4.80175008850711    7.01905839152477
C  5.50345198778589    3.72531315324272    7.14305585186888
H  5.44269792412071    3.16457268425117    6.17718079801077
H  5.84682713702030    2.98519284102873    7.90102393516740
C  4.10464072505858    4.22982977606675    7.48187434904866
H  3.80150729310432    5.00334723310553    6.74930751308141
H  3.37512212945063    3.39553964491041    7.45253967805705
H  4.07237063124554    4.68076486743533    8.49174291349069
C  7.30002501780921    8.02280881616649    8.52605997684206
C  6.05132043189111    7.38071720262753    8.83905215528346
C  5.02936950895478    7.97520564179697    8.02126846488658
C  5.65841747496206    8.97885426363643    7.19471894971831
C  7.06359714257069    9.00948276445986    7.50964958892225
C  8.58094824149890    7.77027491271792    9.25977574907725
H  8.57656726022234    8.30526202106389    10.23462873500761
H  8.71869056454207    6.69324264393105    9.47485273293078
H  9.46166340379398    8.12072272040210    8.69144129756512
C  5.89021942364866    6.34594533928724    9.91302681521774
H  6.56438333030962    5.48039545074680    9.75070580679956
H  6.12966257818957    6.77321208766201    10.91029981043587
H  4.85531064723760    5.95878093502004    9.95417946998646
C  3.55744071708866    7.69700458522826    8.03539848800238
```

H	3.28962281790699	6.93220870269440	8.78774530660170
H	2.98175301756849	8.61849131568296	8.26427638743828
H	3.22796916400521	7.32852777530904	7.04108451808947
C	4.93810596254058	9.90258467628359	6.26428998497108
H	5.64694569469582	10.40613347944444	5.58286877226303
H	4.20788285432760	9.35362772160448	5.64148154035383
H	4.38602667672379	10.68478557447957	6.82987834836323
C	8.07045675391647	9.93257213867871	6.89228149210974
H	8.02552161571733	9.86258164308602	5.78611610878861
H	7.88893053426851	10.98845528034532	7.18490661806771
H	9.10092531240404	9.67007904393564	7.19999195509415

110

2_lin (no dispersion)

Mo	4.28954737890346	5.13182201687421	4.66107216071965
Mo	5.47850502630489	0.77120162557487	6.50757110255856
N	4.72697152406802	3.50180042059054	5.33409868396298
N	5.04050586527203	2.40147615241805	5.83553717960398
C	3.52501404527689	4.66763309789766	2.20334977342064
C	3.11441427604382	4.21370192819877	0.83215185083406
C	2.35447126660900	5.06740140383009	0.00094014544898
H	2.07319733220081	6.06827536525580	0.36385601078447
C	1.96616933240111	4.64973321624980	-1.28331708036252
H	1.37798600555041	5.32584406265717	-1.92333213093207
C	2.32863443919193	3.37403660101352	-1.75063613958613
H	2.02299142707063	3.04694143921963	-2.75667469211802
C	3.08289703101827	2.51752001264716	-0.92923991093064
H	3.36783206896228	1.51652394291107	-1.28893164798445
C	3.47503806603136	2.93436300660471	0.35398797002088
H	4.06143837999825	2.26080434807790	0.99743757291085
N	2.69303157475410	4.78829584096090	3.24763787659428
C	1.31103262500345	4.34548353347248	3.27532896942517
H	0.87033403075894	4.38534331182991	2.25389174149944
H	0.73418466471859	5.06815136110673	3.89351026251302
C	1.14119884329538	2.93769992062898	3.86112817319468
H	1.61673238008280	2.17717298704765	3.20815633740464
H	0.06668820287035	2.67890488759316	3.96666633375607
H	1.62344266867064	2.87609826655812	4.85790588362900
N	4.77102778801510	4.99824523440173	2.56225311363487
C	5.93111772862680	4.99876337593448	1.69309713635400
H	6.45177070146427	5.97724304593970	1.80172940920979
H	5.61402306446201	4.93039454877570	0.62817253914033
C	6.92057310221619	3.87509555184396	2.02341849982702
H	7.19712517982524	3.91280060140056	3.09669485841593
H	7.84335620419316	3.96831143821555	1.41354797321293
H	6.47464071858371	2.87828911100509	1.82802473394725
C	3.30917658346685	7.37900855886597	4.88656946806998
C	4.64790589286238	7.55566935530343	4.43944046283338
C	5.55493610909173	6.89484578379645	5.36267873656699
C	4.71726019225753	6.33710737934745	6.42953966510214
C	3.30960260953534	6.59725078912590	6.11065157638648
C	2.07727182216943	7.91184726995508	4.21193483916870
H	1.20966423648026	7.23717434493999	4.35215995711198
H	2.22551466502420	8.04903784732695	3.12276256720778
H	1.79233988558065	8.90125865030828	4.63380536919753
C	5.07280775708669	8.31141501001185	3.21233746357720
H	4.29887562059615	8.28941109894913	2.41982493010875
H	6.00829651461524	7.90203355469308	2.78221176284629
H	5.26758102488406	9.37998819004421	3.45342256174941
C	7.05025302302218	7.06916904673494	5.39886262633172
H	7.34281792051415	8.02054295670065	5.90285422087912
H	7.48548618089116	7.09213311171762	4.37958016961917
H	7.54315860151920	6.24298642856744	5.94739899713934
C	5.22407019256537	5.75469505557145	7.72359569494933
H	6.19113059524758	5.23275006180768	7.58515073180589
H	4.51185371796474	5.01554706648935	8.14095465277593
H	5.37554574855949	6.54727399437865	8.49067427994848
C	2.13492567132790	6.41718614530913	7.03547891344198
H	1.98545880566923	7.30729191476760	7.69133160212564
H	2.26920928626468	5.54066207892829	7.69900375722816
H	1.19129867323127	6.26295337793091	6.47453634983352
C	6.24231561982826	1.23392763016087	8.96575755394574
C	6.65252541609084	1.68738576777196	10.33720412935610
C	7.41502994535815	0.83466722705609	11.16708729482249
H	7.69851294585361	-0.16513599756343	10.80294516546650
C	7.80298093243525	1.25191713352649	12.45158393800954
H	8.39325236981781	0.57661391243868	13.09052903118568
C	7.43766162636214	2.52623655901712	12.92044414029704
H	7.74321994467595	2.85308773479657	13.92658878144417
C	6.68086965742711	3.38177714433188	12.10035689514085
H	6.39383570565015	4.38175757061984	12.46121087160135
C	6.28908976068188	2.96537581811413	10.81686829893416
H	5.70063788493748	3.63816453477311	10.17448023296497
N	7.07466703774122	1.11386912789392	7.92160475978925
C	8.45600477355054	1.55876996869531	7.89399343137074
H	8.89684491571238	1.51924406749796	8.91538003747929
H	9.03395424797665	0.83720927371878	7.27554103767539
C	8.62347715779486	2.96708595757709	7.30881404677569
H	8.14699038564872	3.72657453274813	7.96230271403213
H	9.69753142658527	3.22757411711708	7.20282317500652
H	8.14053185620049	3.02839732881752	6.31234515808297
N	4.99652424870154	0.90324746803784	8.60629111851604
C	3.83609494280033	0.90137789054146	9.47498178722422
H	3.31583587566708	-0.07713395720999	9.36487748147510
H	4.15270609032965	0.96859942847875	10.54012768809615
C	2.84655365818243	2.02519667310666	9.14540154856953
H	2.57102225886758	1.98889902381370	8.07181379242619
H	1.92325602358078	1.93088255335351	9.75432460527751
H	3.29202743652197	3.02185870472179	9.34258674879407
C	6.46022006219871	-1.47525837271370	6.27988187315844
C	5.12164935097403	-1.65303302423442	6.72710418768234
C	4.21410488243007	-0.99187546535300	5.80458455867503
C	5.05132672249009	-0.43272103337832	4.73810473627061
C	6.45917192720580	-0.69238231662540	5.05653190101554
C	7.69260219953205	-2.00792310132897	6.95376470206793
H	8.55954082860116	-1.33214414361376	6.81472174394188
H	7.54440401314108	-2.14701359298515	8.04269668883010
H	7.97856833413125	-2.99635269217243	6.53029783592698
C	4.69714115551967	-2.40990912084918	7.95366858456082
H	5.47228465326425	-2.39040536648010	8.74505225948880
H	3.76299228214627	-1.99952007974092	8.38580602513420

H 4.50021180170151 -3.47778656298414 7.71124685951057
C 2.71889115902295 -1.16713177720869 5.76849318853721
H 2.42690014962386 -2.11844034991064 5.26404009446085
H 2.28381308876431 -1.19085009269651 6.78782005471478
H 2.22543157296885 -0.34099190674319 5.22038909952693
C 4.54389633206453 0.15075080509658 3.44477199102678
H 3.57707654730247 0.67285512902773 3.58432367183927
H 5.25607156811500 0.89005864451238 3.02761457624269
H 4.39171754460142 -0.64118023126144 2.67716788830565
C 7.63355597041495 -0.51075477136674 4.13161667394267
H 7.78299358661491 -1.39987625704168 3.47441776251138
H 7.49900346994202 0.36671925957707 3.46940264997417
H 8.57731637937758 -0.35724350178536 4.69254088278057

110

2_int-1 (no dispersion)

Mo 4.66659609858929 5.04937724571257 4.68291254460165
Mo 5.48754998922833 1.68403784985552 6.48126036531389
N 5.58721334596936 3.53837173985188 5.26464807394282
N 6.66693330847148 2.86088349735947 5.52420189251932
C 3.45211722386474 4.62724003232357 2.36619432579441
C 2.80221949266950 4.50810532927307 1.01900789892486
C 1.98364683423326 5.54886142566028 0.52186563135800
H 1.83029717900605 6.45400857821835 1.13014919847992
C 1.37562275209811 5.44053703034693 -0.73965057050013
H 0.74441847512449 6.26154733641511 -1.11431168045727
C 1.57484820730357 4.28990712590453 -1.52371187894617
H 1.09782678634996 4.20514429606121 -2.51254493973776
C 2.38583524128218 3.24879276486655 -1.03905614152903
H 2.54437105300339 2.34388356483258 -1.64632161545540
C 2.99491985538372 3.35632504064125 0.22276341301106
H 3.62136028297984 2.53456077656572 0.60238601371636
N 2.80441722057663 4.68077511728623 3.54473176142536
C 1.38135130341599 4.38787741680062 3.67589704149453
H 1.25474542356091 3.30656261836697 3.92289028435022
H 0.86377621686136 4.53089739757749 2.70198035322509
C 0.67747727906989 5.21346556100450 4.75142758595435
H 1.23156703247586 5.16054813585678 5.71113415702460
H -0.34918582058763 4.82837705583500 4.92063093593987
H 0.60133281406959 6.27949985311532 4.46314487578091
N 4.76975307732225 4.70971561869905 2.57516576487240
C 5.80149072560320 4.68156033610992 1.55750879769996
H 6.44663657631062 5.57991860477661 1.67442510841266
H 5.34951423397736 4.75233667644917 0.54321885035568
C 6.68212430398367 3.42971596211676 1.66240929521775
H 7.09592674553152 3.33327132806758 2.68651008271083
H 7.52373302320913 3.47980071712229 0.94020594487919
H 6.09819510463699 2.51063790141126 1.44941877533017
C 3.97116654705963 7.41238264596604 5.09498958765555
C 5.03471082867318 7.34096090204091 4.14425268089321
C 6.18636030717414 6.68113989541428 4.75806814597109
C 5.77039183206301 6.34392842793268 6.11528953935542
C 4.38197655590246 6.76322693268557 6.30796397430002
C 2.67313719723200 8.13199648841212 4.86773645400804
H 1.90281672500989 7.84865757153829 5.60896686432434
H 2.26259136761465 7.94530728676722 3.85533225935990
H 2.82837524088164 9.23062618422064 4.95433517956570
C 4.99568356128327 7.96017441206190 2.77390959273994
H 4.03224191191167 7.77427928700538 2.25713052404015
H 5.80214746957898 7.57312159555481 2.12274259389203
H 5.12536149799983 9.06390274468526 2.83308886371382
C 7.60688045805885 6.70822761532590 4.25832898199411
H 8.14222741335918 7.62776893715462 4.59261602677961
H 7.65521152184434 6.68942424818519 3.15152658031243
H 8.18171286588132 5.83639793625919 4.62789502273944
C 6.69922522313715 5.85284291379723 7.19166029726363
H 7.41279009981472 5.10011640373693 6.80021367387158
H 6.14362050381665 5.37563540768348 8.02034053716713
H 7.28836235557230 6.69215056074398 7.62483506111394
C 3.62771970641675 6.76479265902730 7.60986238372134
H 3.84445001717112 7.68123400140956 8.20705528904963
H 3.89766108742219 5.89603376820080 8.24135866304631
H 2.53171801742513 6.72906952677173 7.44921847579373
C 7.04360147255321 1.73756749845547 8.55177986455897
C 8.07254078024248 2.06347896294587 9.59620351159769
C 8.38556383810459 1.13081686833001 10.61088348311691
H 7.86043786521765 0.16331959221988 10.64001588633265
C 9.35628949329880 1.43280923923487 11.58100796478973
H 9.59098003132561 0.69743581377788 12.36640321334298
C 10.02472887261037 2.66951024082245 11.55009386175470
H 10.78461517185561 2.90592967815972 12.31112984119796
C 9.72111948333467 3.60192912078738 10.54253294720599
H 10.24463919625430 4.57012806141294 10.50979873645050
C 8.75234299715525 3.30153622455711 9.56982039785872
H 8.52429358400473 4.02781567155518 8.77513472411822
N 7.25791570948120 0.93126555806971 7.51221706355228
C 8.53408196876654 0.31043676039323 7.19875258546794
H 9.11715641557026 0.14815918278645 8.13308821254267
H 8.32584702613114 -0.70084828358730 6.78443599427780
C 9.38026847828508 1.09708930018181 6.18954327156882
H 9.67180087115970 2.08502742174058 6.59956086975160
H 10.30488477195424 0.53490096408881 5.94062657708254
H 8.80899349778131 1.28056886557121 5.25931009624183
N 5.80490504242574 2.27573698671454 8.50259728854434
C 5.28186275503257 3.20887020368474 9.49293670029418
H 6.00722166347318 4.03635847595773 9.67010319831515
H 4.38436303331192 3.67885474897838 9.04101901956056
C 4.91009685324157 2.59041472066113 10.84885016596590
H 5.79381197808712 2.13803175896190 11.34195627338042
H 4.50982880846630 3.37320706131122 11.52682000476605
H 4.13949420802437 1.80408699077437 10.73865425880219
C 4.74078744969892 -0.59425006993572 6.93068191639713
C 3.66745034425933 0.19044878534823 7.44050291589942
C 3.22576845728300 1.09671081139108 6.41769013487348
C 3.97010845552503 0.77373411656600 5.19050099526010
C 4.94872175685835 -0.25783568332746 5.52449965517316
C 5.46851884317499 -1.66693545067684 7.68873310408279
H 6.45307243662410 -1.89206306636519 7.23708439332038
H 5.64258135886553 -1.38122836794537 8.74535396925840
H 4.88419812717805 -2.61404369373276 7.69366546501784
C 3.08772068688183 0.07561513982361 8.81865175469968

H 3.84512366014125 -0.23662535885687 9.56380052750409
H 2.63585788574858 1.02687410656204 9.16123650100822
H 2.28102523475056 -0.69071793096131 8.83305361814902
C 2.02883594050646 2.00235612379073 6.50707566458898
H 1.10254167331520 1.49115864102844 6.15642032773256
H 1.84685565043145 2.33524183228761 7.54831101507333
H 2.16661444652682 2.90992590379377 5.89136375501860
C 3.62811442184349 1.25681103975463 3.80872572509716
H 3.44590886304457 2.34969891601536 3.79203435237445
H 4.45130875509798 1.05219803228910 3.09708239484557
H 2.71600336377876 0.74888325280690 3.42216494532571
C 5.78066988744641 -1.04666004741854 4.54718666940944
H 5.22262286363524 -1.92867406138721 4.15613208352345
H 6.07870119699687 -0.43172164703108 3.67562990117624
H 6.71121927563604 -1.42715872703160 5.01270420610732

110

2_int-2 (no dispersion)

Mo 5.72475524584807 6.96253775990163 3.66175539562902
Mo 6.82267303810729 7.04328516876750 7.28531632022691
N 6.95346733195169 6.92640021424718 5.37832478658099
N 5.59308119429062 7.07983779405288 5.56865036244321
C 4.55762266245357 9.24019576452690 3.02994748943849
C 3.90158780619460 10.53786188391723 2.66936948088380
C 4.11990161050338 11.69829343806735 3.44780099157781
H 4.76722954546869 11.63720845222636 4.33563468435020
C 3.50940905454168 12.91582627368040 3.10460826959959
H 3.68608752218830 13.80884357046311 3.72434997785498
C 2.67299298804972 12.99457349711937 1.97674563338181
H 2.19513210400141 13.94948354987973 1.70777914510677
C 2.45047707890669 11.84799238610663 1.19333525238930
H 1.80122230707953 11.90336443487156 0.30552304311271
C 3.05816275408114 10.62917826282239 1.53720212087339
H 2.89064317459100 9.73692156301030 0.91422010200679
N 3.91037966189718 8.09208826452493 3.28849982807783
C 2.48169240813231 7.95788433679650 3.50978397296545
H 2.11962071539215 7.08007275814357 2.93005528096334
H 1.93894124286869 8.84106209298199 3.10591617387319
C 2.11453388213820 7.76025067140672 4.98522408036974
H 2.68529096535995 6.91270037327991 5.41476528380980
H 1.02931586770843 7.55274971683604 5.09618887316057
H 2.35464318268315 8.66599384716857 5.57853834930585
N 5.88259142778625 9.05493778575367 3.14038707602998
C 6.90297492018375 9.96918398072698 2.65930468829830
H 6.43585582725833 10.87915614838374 2.22062122967814
H 7.45836275327685 9.47907824310353 1.82796576750369
C 7.90864830290100 10.36316977195532 3.74517460302963
H 7.43529320089808 10.99868616766930 4.52137271409855
H 8.75678643415442 10.93079435983970 3.30890865561057
H 8.30658479635271 9.45739785351497 4.24625889803702
C 5.03211035678059 5.88876598127256 1.56266973333492
C 6.31328360253362 6.45047175065778 1.30982332175748
C 7.24545369528089 5.94106076643124 2.29162179947310
C 6.51617654156158 4.96578318672914 3.11587330635847
C 5.12331113551231 4.97623451617394 2.69258117771947
C 3.80401549060114 6.14238104268857 0.73706379356781
H 3.82494117713681 5.52956170834004 -0.19146580803934
H 3.72335427957103 7.20348771084908 0.42707693912372
H 2.87723167078408 5.87711122796264 1.28091731301782
C 6.63176849024587 7.39432459657198 0.18536059266015
H 5.84954129196250 8.16963322899868 0.05487238593234
H 6.70651702909855 6.84381189050258 -0.77842719335104
H 7.59954520329094 7.91016164905133 0.33835563073514
C 8.73800901678891 6.12849590564571 2.28069040519849
H 9.02498089191740 7.11413354736346 1.86296770576127
H 9.24507434443861 5.34888226847550 1.66642283108666
H 9.15693366108984 6.07007232924716 3.30302075477827
C 7.15528915337478 3.98559184240847 4.06028800324734
H 6.41884306261374 3.58719228962331 4.78358194511264
H 7.96298406533256 4.46004113751422 4.65080105409715
H 7.59109061278331 3.12144690673493 3.50909987039737
C 4.05115516352169 4.00882982941107 3.11972485712858
H 4.19903333816458 3.67493204489735 4.16447292476386
H 4.04190418713158 3.09791546090120 2.47723260630188
H 3.04109740357607 4.46113517218976 3.06053077086525
C 7.98769164568441 4.76435717642712 7.91658317431072
C 8.64241857111202 3.46529826666544 8.27496231083275
C 8.42411227375112 2.30700006320120 7.49334766295070
H 7.77745232324868 2.37074126516324 6.60521875378104
C 9.03337997505823 1.08817342259021 7.83412841212846
H 8.85652093622186 0.19679167632119 7.21208800430685
C 9.86878125226805 1.00605891573905 8.96250082039556
H 10.34551512650843 0.05008264515539 9.22967438889570
C 10.09158694501577 2.15057255075599 9.74881822995693
H 10.73994205428328 2.09254488921474 10.63711507435690
C 9.48483050352033 3.37058534249507 9.40754627864604
H 9.65316477608766 4.26135093454723 10.03242608485641
N 8.63623213519185 5.91204249418028 7.65925556275127
C 10.06531425220698 6.04462426775864 7.43914437745698
H 10.42717097044134 6.92359188626279 8.01716302908858
H 10.60690732415455 5.16196729243029 7.84568457728041
C 10.43423315014491 6.23874148084631 5.96367220323827
H 9.86513408479455 7.08624175892902 5.53189441636122
H 11.51982077305355 6.44472191416355 5.85338651679526
H 10.19377806184328 5.33198984778280 5.37203910627411
N 6.66296697734866 4.95091448707471 7.80610386193328
C 5.64149412553639 4.03756218365307 8.28657618230439
H 6.10714635210574 3.11989582763739 8.71044984641773
H 5.09629257157958 4.52101564589385 9.12863739412023
C 4.62446441516649 3.66219648437035 7.20490214053730
H 5.08834076899059 3.03494085173930 6.41642934115777
H 3.77776713935314 3.09262702361807 7.64142320709689
H 4.22620228354648 4.57598971904150 6.71889667030193
C 7.51744794334268 8.11954824828338 9.38301524194378
C 6.23769252032835 7.55553635426781 9.63760403679615
C 5.30329364995955 8.06302255124424 8.65666527924189
C 6.03005742285760 9.03923408437898 7.83120104522339
C 7.42341253931169 9.03126188980128 8.25287141224668
C 8.74687364010641 7.86830275449610 10.20732774046338
H 8.72513818056530 8.48016695082503 11.13644473805759
H 8.83011447582666 6.80719090923644 10.51658291184531

H	9.67267979549967	8.13605986855613	9.66303455807997
C	5.92219308865408	6.61165224555303	10.76290235485177
H	6.70437044187973	5.83582844663730	10.89094280200815
H	5.85125066883887	7.16208188695325	11.72704911852712
H	4.95341599181453	6.09685439825972	10.61287546697920
C	3.81113551692347	7.87267249003288	8.67021119854827
H	3.52741186016130	6.88642146976809	9.08868215884457
H	3.30346350282760	8.65122223934436	9.28532964947711
H	3.39020635817027	7.93001671153034	7.64863031006893
C	5.38890727029121	10.01810074178905	6.88690719291071
H	6.12134085210321	10.40801820639332	6.15504981516920
H	4.57348568534740	9.54527530396891	6.30590528491825
H	4.96272631543463	10.88765680986833	7.43710312124953
C	8.49356733983726	9.99992166683331	7.82370683784042
H	8.34355303737816	10.33332746020979	6.77914488432330
H	8.50256561987201	10.91100922854829	8.46593948779264
H	9.50435705784470	9.54902370469909	7.88118811123076

110

2_dia (no dispersion)

Mo	5.76512804082839	7.10740188681108	3.77110731141595
Mo	6.54630486736293	6.98802381735346	6.31095769585473
N	7.36490839671050	7.58979098121711	4.70856857093191
N	4.94541072275525	6.50869670015385	5.37543097200990
C	4.56302668091196	9.35248128360225	2.87231092813457
C	3.92075481314400	10.63850697807699	2.44427058241722
C	3.04237654608447	11.34505500585367	3.29685915643296
H	2.83011472094175	10.95345873687506	4.30255582753039
C	2.45364714778538	12.55004406584450	2.87826934961115
H	1.77768918732543	13.09162664844105	3.55821922947561
C	2.72656123984430	13.06362361525456	1.59838996625722
H	2.26232284914446	14.00659325324376	1.26999686613434
C	3.59621899717842	12.36767187277166	0.73981842832052
H	3.81308813580111	12.76174429633195	-0.26529983739767
C	4.19148126892495	11.16764933960823	1.16089137774335
H	4.87057312001727	10.62346813134773	0.48689416237142
N	3.90666053856099	8.27106663959584	3.29451284279491
C	2.46554085758896	8.08690878496752	3.27039360710323
H	2.24227210278520	7.12373241617725	2.76537543955712
H	1.98722822931448	8.87882057509644	2.65065365284588
C	1.82158250326374	8.04654589422137	4.66083713444699
H	2.31010283682633	7.26988544110875	5.28038870946941
H	0.73966091465734	7.81173587712711	4.57797474837042
H	1.92031131216522	9.01614294658583	5.19100563055067
N	5.88671507457070	9.13676662695945	2.85626341605949
C	6.88736178934556	10.18991192692052	2.92126434590563
H	7.44121399564532	10.06208333363745	3.87990332504769
H	6.39452675479124	11.18670323184497	2.96347220175970
C	7.91166654550583	10.16985518128639	1.78337433794533
H	8.43530883804736	9.19459881272477	1.74352535642175
H	8.67571556554947	10.95890395889056	1.94471209857319
H	7.43891917418974	10.34893063815487	0.79618112464093
C	4.87786618836789	5.99893387256995	1.55857152654470
C	6.15658944953613	6.56733182501143	1.22455965635221
C	7.14888070457673	5.99869299948101	2.08103507597247
C	6.46397931332847	5.07998560263067	2.97675350962285
C	5.04993657264818	5.07191167939005	2.63550749398575
C	3.63723638105281	6.24164400771774	0.74996551704574
H	3.75968304109680	5.81267222809672	-0.26949575334807
H	3.42494003789635	7.32200606695393	0.61720765410226
H	2.74238958347944	5.76785683585556	1.19633216399596
C	6.37037425405728	7.49462185813029	0.06577317693366
H	5.73881122901862	8.40324896467970	0.14343065145495
H	6.10112410979677	6.99208411535287	-0.88942687434821
H	7.42169549897513	7.82699455835273	-0.01318383677568
C	8.63200292290152	6.21498610883130	2.03169463958579
H	8.92299469123932	6.83423108336912	1.16078570297139
H	9.18398911690712	5.25383578226550	1.95543820752226
H	8.98131173113854	6.73222750948819	2.95198409755200
C	7.15623036403580	4.05777776554010	3.83489226703445
H	6.49191591497905	3.66777485815007	4.62699071228871
H	8.05421085538271	4.48105130789065	4.32221330137487
H	7.47996623039341	3.19397640186699	3.21113813472874
C	4.01310427477484	4.17477439387252	3.24566592477687
H	3.93423524627500	4.35671096062589	4.34068351616745
H	4.25505042347618	3.10015053515566	3.09607299761957
H	3.01181103145010	4.35408026658583	2.80716851930275
C	7.75366060258912	4.74357942574813	7.20607259630033
C	8.39784184095378	3.45778606300638	7.63185897758136
C	9.27748423797504	2.75417928234975	6.77805435268043
H	9.48967875960075	3.14800268836963	5.77318038746891
C	9.86798500066531	1.54931086186065	7.19446047539996
H	10.54501853362483	1.01015624730334	6.51365079375036
C	9.59542109737490	1.03269606361899	8.47319820769925
H	10.06106677787860	0.08982205175637	8.79986445179168
C	8.72429500287672	1.72550219146603	9.33282350544265
H	8.50773499815911	1.32911396614173	10.33709571173037
C	8.12747916480874	2.92553801692757	8.91400019319809
H	7.44703451022302	3.46713090448238	9.58868954237021
N	8.40821841767998	5.82634490253411	6.78478576658521
C	9.84902474670811	6.01277269329557	6.81060236057672
H	10.07111208679668	6.97266390492719	7.32241341379867
H	10.32826366908196	5.21735441704654	7.42510382145516
C	10.49438420975369	6.06299309580247	5.42112585803020
H	10.01125890565606	6.84755269238494	4.80725773193642
H	11.57732280270766	6.29174669193268	5.50776398694726
H	10.39161783164711	5.09860249128178	4.88236065933744
N	6.42957079245702	4.95734148200143	7.22284009675656
C	5.43013257650255	3.90299799743393	7.15743042328622
H	4.87575322901367	4.03044941878092	6.19909672630817
H	5.92421647660874	2.90683273271500	7.11472475422611
C	4.40601994599450	3.92111183467109	8.29554160607624
H	3.88148301186064	4.89583758233642	8.33636275193050
H	3.64272370325488	3.13145075773820	8.13359221865879
H	4.87898160467740	3.74153513872008	9.28253761983997
C	7.43028977844331	8.09375498862225	8.52631246281334
C	6.15125214599767	7.52539492185949	8.85940407673663
C	5.15943004478530	8.09508826559624	8.00330366078797
C	5.84473081644896	9.01430154709664	7.10854167794520
C	7.25848958547050	9.02203297336769	7.45030878430499
C	8.67023331747420	7.85063501173967	9.33590783123850

H	8.54489462518799	8.27477779851354	10.35702908513999
H	8.88530178099439	6.77028495142243	9.46441943563503
H	9.56461138068131	8.32893981253970	8.89339221301447
C	5.93689259774615	6.59709102585183	10.01726016409170
H	6.56762958893111	5.68798779420220	9.93840195031547
H	6.20699157502333	7.09829331073239	10.97292819675178
H	4.88523753086835	6.26577042809462	10.09618208441347
C	3.67667103468023	7.87638786912812	8.05037057841447
H	3.38460413267707	7.26234124142738	8.92462668540155
H	3.12257556422555	8.83688127852864	8.11842960934567
H	3.33104959357502	7.35244818841307	7.13228349304251
C	5.15378273361431	10.03632158529574	6.24906181178563
H	5.81928845157835	10.42529884733277	5.45739073324559
H	4.25612378177243	9.61329237619844	5.76098753042866
H	4.82996523455001	10.90087536686605	6.87172485068663
C	8.29485862506972	9.92069138521749	6.84181716784718
H	8.36615927754414	9.74792536205725	5.74487136779150
H	8.05776138017626	10.99489555336307	7.00210950724064
H	9.29828195100143	9.73397603394951	7.27222050921867

96

3_lin

Mo	4.37724174970483	5.22932666552576	4.90357918135999
Mo	5.39018611111583	0.67306824458605	6.26567300234266
N	4.75981724576244	3.54296880711337	5.43050894249411
N	5.00629358487374	2.35917470944973	5.73887190229493
C	3.71706666462672	4.61665801942253	2.56199981141004
N	2.80836320910944	4.93249775565912	3.50201877464866
C	1.49879088358877	4.31909972499812	3.61262119636398
H	1.03514494659356	4.21134185918740	2.60510068306038
H	0.85139350509403	5.02497500098145	4.17615681333354
C	1.51857745356112	2.96688548706324	4.33523301049536
H	2.11793744677198	2.22191613345007	3.77447786715888
H	0.49215346326174	2.56476837379120	4.46077301354676
H	1.99209131862885	3.07757980958230	5.33108155870026
N	4.89513958560868	5.20242095590243	2.84299367166334
C	6.13673219608464	4.91657260535335	2.14954605502592
H	6.80802917222966	5.78820305533899	2.30592268179074
H	5.96147689119111	4.85407451813824	1.05081919168619
C	6.83482719344193	3.64597726614719	2.64877822071050
H	6.99254548612600	3.70704569939606	3.74408470708539
H	7.81316848261927	3.50224427061864	2.14557759758301
H	6.21259078315078	2.74791914959793	2.46164628106919
C	3.42903699616645	7.45226419641800	5.19346571210693
C	4.77604936401924	7.62698893496260	4.76874770602306
C	5.65913346883386	6.93234706200597	5.68286715578337
C	4.80527996149400	6.34830332958809	6.71930664611673
C	3.40792203752223	6.64022013440735	6.39274568262766
C	2.21129334420151	7.96071606668447	4.48228756344632
H	1.34454239125205	7.29033374001743	4.64219596873272
H	2.37872636498589	8.03540369228539	3.39033101902002
H	1.92310194723871	8.96970062265662	4.85000369235811
C	5.22072769847560	8.35062129383660	3.53345090287382
H	4.45273947877221	8.30670648960274	2.73702253130250
H	6.15154618271540	7.91125550984176	3.12498831170789
H	5.42523077668601	9.42245216998568	3.74717095979442
C	7.15821110752076	7.02543318759856	5.71024182130685
H	7.50802564710346	7.93316723074311	6.25495032701681
H	7.57828987833738	7.07205095913282	4.68587421251077
H	7.60749693451005	6.14534431497301	6.20991630742475
C	5.28857489498154	5.68884171147104	7.98046930488653
H	6.24453225832895	5.15531908228783	7.81208019667057
H	4.55681217212191	4.94264223159379	8.34709201070365
H	5.45341873050301	6.42882915451557	8.79454372062854
C	2.21501418731476	6.38202189117655	7.26854790429636
H	2.03602596492024	7.21807068412414	7.98409840381891
H	2.34538628185011	5.45785742603191	7.86436981399403
H	1.29251064407710	6.25802447793034	6.66726839651019
C	6.05083258260121	1.28691850495295	8.60646603689544
N	6.95952181631170	0.97115049456218	7.66636673993666
C	8.26829838737843	1.58613272330088	7.55465784476373
H	8.73128338944091	1.69731406717648	8.56209449858335
H	8.91707984233755	0.87977670209713	6.99329231122717
C	8.24668056220955	2.93633985450131	6.82836551696864
H	7.64441044844416	3.68135040701746	7.38595920237962
H	9.27236226528692	3.34054822366107	6.70347442697268
H	7.77508548154217	2.82185334828779	5.83204746074848
N	4.87317732354004	0.69978192884743	8.32640498211204
C	3.63153651469523	0.98542516351084	9.01988294452952
H	2.96138987593440	0.11242351353401	8.86612846082273
H	3.80733509760538	1.05095864759178	10.11833217913645
C	2.93132082985168	2.25373002273383	8.51780621673906
H	2.77227461551986	2.18922281368074	7.42289553221772
H	1.95353430806751	2.39798964849694	9.02192456073199
H	3.55303460745034	3.15291825485816	8.70120386478610
C	6.34029201978020	-1.54883049055686	5.97440715151180
C	4.99380966429406	-1.72486039915453	6.40028810095915
C	4.10928216510700	-1.03081447739263	5.48706429644696
C	4.96172997033077	-0.44578079506971	4.45001744788157
C	6.35960287520025	-0.73654232842235	4.77527571356807
C	7.55918221651292	-2.05612979501117	6.68451484047699
H	8.42528177970459	-1.38522373705819	6.52327671226118
H	7.39308901346373	-2.13043077136226	7.77672655439227
H	7.84762809503298	-3.06510646112280	6.31698732119364
C	4.55085307271855	-2.44888218433716	7.63598268629736
H	5.31975294702523	-2.40478068998984	8.43151571358539
H	3.62041164490754	-2.00977311811785	8.04557090676328
H	4.34647263078544	-3.52075619200614	7.42236578691178
C	2.61026014174173	-1.12530950707535	5.46081487741605
H	2.26090142742062	-2.03329440158113	4.91623301368191
H	2.19098681588808	-1.17256410365510	6.48548735116840
H	2.15982936195385	-0.24557365458253	4.96154784588780
C	4.47666156130683	0.21402894533671	3.18970354847436
H	3.52116865408632	0.74779018429388	3.35991901208283
H	5.20818072524711	0.96007585874145	2.82228790358099
H	4.31033855327120	-0.52576210530517	2.37575472373534
C	7.55154302738796	-0.47654736247036	3.89871941601319
H	7.73102716822450	-1.31209241560983	3.18270238455321
H	7.41945392935138	0.44770029754798	3.30340900387040
H	8.47431197696900	-0.35168017325103	4.49942164050008
C	6.28078356683930	2.25876700376963	9.73222116818624

H	5.62866300697348	2.03319222535636	10.59668041724110
H	7.33533589077843	2.24460112096166	10.06588611866763
H	6.04787777612241	3.28856390873577	9.39286503545819
C	3.48669624809062	3.64608757154013	1.43522682634697
H	4.13876952507391	3.87242052585453	0.57093989588819
H	3.71971594227706	2.61603301307487	1.77369836032378
H	2.43207490016540	3.66056971546647	1.10181146025513

96

3_int-1

Mo	4.78142215965772	4.86598309045371	4.66950488030920
Mo	5.60627759686202	1.65978757199004	6.23936530540692
N	5.84803218867340	3.39587254642172	5.04329268788078
N	6.94211978087913	2.81873218589602	5.46875686956268
C	2.62017055828210	4.70863501078547	3.26582257573742
N	2.67108480514135	4.65386758010695	4.62103008404125
C	1.50073697418895	4.93095149772595	5.43474193981979
H	0.62867356016198	4.36196963813938	5.03617780860192
H	1.20952633059551	6.00900925260227	5.37099564333801
C	1.72602949056325	4.54987391022257	6.89159968900261
H	2.03449187636842	3.49061804503734	6.97190490511363
H	0.80480760442407	4.70222838845917	7.48896816140997
H	2.53559672116401	5.15986285923704	7.32884336962532
N	3.83315741703906	4.43748333645996	2.78263042038896
C	4.18883283864396	4.51916461064788	1.37664077966081
H	4.17813475255096	5.57675716733473	1.02408801919961
H	3.42970635465608	3.98288968983225	0.76261429173281
C	5.56864457700400	3.91434199884306	1.12512289910964
H	6.29946997313703	4.33347462412257	1.84516979122086
H	5.91317435168162	4.12776785481255	0.09345962482785
H	5.55979935320011	2.81806055482677	1.27230667504337
C	4.66106651476737	7.13978992722626	4.67581925373697
C	5.78502376831017	6.81282486756299	3.82901578247002
C	6.76689430549350	6.16574248422397	4.64537324044188
C	6.25758345169278	6.09229183055378	5.99225624145501
C	4.94122616088498	6.69159937482285	6.03786967524889
C	3.43913520410742	7.89925415257225	4.24175076089089
H	2.54769586508939	7.61311396568188	4.83378784845218
H	3.20170413648213	7.71873101273628	3.17441998642714
H	3.57609037021860	8.99680946958158	4.36480056568270
C	5.96795885562257	7.25667588443375	2.40889613612678
H	5.00017248228997	7.35209979521743	1.88062036281312
H	6.60243574351046	6.55680447248464	1.83298205136175
H	6.45744812431822	8.25535932185729	2.37662938193709
C	8.10846299376743	5.65796112319251	4.20797059242708
H	8.92198806399768	6.33221452690665	4.55587187642437
H	8.17740679700909	5.59119587242507	3.10472931336566
H	8.30112006957901	4.64931313175671	4.62828613794258
C	7.03320994825878	5.55012911485151	7.15697982829028
H	7.39065495787652	4.51846652305983	6.95160418495513
H	6.41749965759606	5.52671042916931	8.07453344041840
H	7.92517646949451	6.18022344368320	7.36266432996821
C	4.21333665574007	7.14539040981203	7.27088880057422
H	4.59490276253371	8.13053728857099	7.62573299026238
H	4.32876760688843	6.42705391698608	8.10544929383998
H	3.12916329719478	7.26667694793764	7.08200391116134
C	6.56462291221914	2.01798434620490	8.58747484714603
N	7.06963436412886	1.12760391133038	7.73652653627899
C	8.43350648533921	0.64279181060446	7.76694861508270
H	8.73550548812707	0.41306720083738	8.81559828944509
H	8.45396409722714	-0.32786363630216	7.22813495515301
C	9.44195241694581	1.60009609327010	7.12087024499230
H	9.48889581837172	2.56000264422017	7.67451657303388
H	10.45812829218999	1.15386277562574	7.11118257126217
H	9.13270238717381	1.83418705714030	6.08471394587500
N	5.33481539863906	2.41001540622122	8.18450969236781
C	4.60501670043522	3.50966832622519	8.78767456878652
H	5.29238461630581	4.16528838434423	9.36695641713587
H	4.21722177293473	4.13163719462845	7.95459213973719
C	3.44917734294270	3.06574294946165	9.68541728033174
H	3.80928822134049	2.43655517295233	10.52576713382818
H	2.92517680833013	3.94681568167484	10.11047158377707
H	2.71236430610198	2.47373451200878	9.11060932946969
C	5.00243596843136	-0.61883041376374	6.62197561622713
C	3.74062466942380	0.04057608844955	6.71043719023110
C	3.51964211068019	0.77942704890777	5.50643005924015
C	4.65566503782130	0.52864147352963	4.61798225562775
C	5.58976616495583	-0.34866208362498	5.31440133904290
C	5.57674863128113	-1.50644482516991	7.68443815108000
H	6.65508125586233	-1.68923871368964	7.52356150829369
H	5.46058115835492	-1.06077855055420	8.69223338319623
H	5.06715638277837	-2.49513290540828	7.69304591361074
C	2.83158745929892	-0.03223195628468	7.89759558267074
H	3.39133818772729	0.11031361094072	8.84310990590815
H	2.03681545099169	0.73652080355315	7.85323351087713
H	2.33628053390373	-1.02577286430620	7.95378666378954
C	2.25203544645002	1.45339350534477	5.08159138007223
H	1.72934658466028	0.84361752146601	4.31143594142915
H	1.55484111372787	1.58135993661908	5.93203235298243
H	2.44369723137664	2.45627647712189	4.65175040579280
C	4.72727246793496	0.98530872717385	3.19103255760397
H	4.38544871738519	2.03626640878259	3.09883129578036
H	5.76511348736723	0.94027932800484	2.80797244948677
H	4.09340545995168	0.35383330810479	2.52914322605123
C	6.78272958138976	-1.03618915556942	4.71378553312696
H	6.49981446208814	-1.99904402191059	4.23030447848636
H	7.26522052572320	-0.40338844572153	3.94378345038535
H	7.54978701734564	-1.26378456551943	5.47953587550400
C	7.28612249756761	2.59722386487069	9.77508474668175
H	6.60268837197731	2.71270916100081	10.63827208369015
H	8.13417148795569	1.95607834557500	10.07751107304530
H	7.68952369026811	3.60130375733966	9.52911307121566
C	1.41090000890928	5.13155868744226	2.47937025062870
H	1.56362505117196	5.00701434602577	1.39213022385760
H	0.51958442177203	4.54420072895224	2.78007269353412
H	1.17181001759829	6.19883036231073	2.67656016619212

96

3_int-2

Mo	5.74508378160999	6.87413215724164	3.69353076906107
Mo	6.80492069194048	7.13372838992525	7.25296437423918
N	6.97181960001068	6.93808527816750	5.37602570757779
N	5.57838095510973	7.06942641694637	5.57037010913459
C	4.54560799275108	9.11434113504668	3.12820253433949
N	3.92642265415346	7.93311751187952	3.25682904753718
C	2.51510843976227	7.73933209600461	3.49653608486059
H	2.21237719563478	6.79131493099016	3.00282534548448
H	1.91402394931504	8.54198063184453	3.01050371148612
C	2.17941935996335	7.65564818725456	4.98821075277829
H	2.81851555997768	6.88907450198205	5.46987671750657
H	1.11330988259626	7.39268369366995	5.15021845119976
H	2.37981175465269	8.62344655060483	5.49128157990851
N	5.87294212021312	8.93572186412634	3.12741814551608
C	6.83602672323219	10.01586717711682	3.07297881418017
H	6.48530137341254	10.88407928560584	3.68006969439935
H	6.94344072051925	10.39841163350285	2.02807418169982
C	8.19626426714413	9.55889378212983	3.59318089833649
H	8.09989167596014	9.15068811482539	4.61981286060549
H	8.92134111342716	10.39789165942173	3.59323327710510
H	8.60200465577888	8.74180340144252	2.96682913337654
C	5.12660019938692	5.75960053745273	1.62738028679807
C	6.42184964977773	6.30489597469457	1.41613053284249
C	7.30776077116270	5.79912098998544	2.43583765893119
C	6.53616915152301	4.86874481816971	3.26891435850154
C	5.16247075486515	4.88194506422272	2.78735163434222
C	3.92331245362138	6.04695625856120	0.78140394504479
H	3.96398001396551	5.47524220308885	-0.17112740276759
H	3.85303043598605	7.12262763893286	0.52458220675567
H	2.98621559402111	5.76307044437772	1.29594347655124
C	6.78093713057171	7.30840973648453	0.36150891733718
H	5.93256042889018	7.98807116769099	0.14788425726079
H	7.06002979837915	6.81126293563733	-0.59310208935733
H	7.63895936925076	7.93350857464814	0.67578851670287
C	8.79481848953162	5.97835358045486	2.49936810725603
H	9.11875101773473	6.88427476193376	1.95065481090537
H	9.32842088993019	5.11135158160630	2.04807415044807
H	9.13955635130575	6.07848413124986	3.54472152583533
C	7.11146696327952	3.96957150960397	4.32425214839267
H	6.33531938459985	3.65449764178071	5.04762604378446
H	7.89332181942227	4.49783417631432	4.90351963799476
H	7.55897080273634	3.05043403757121	3.88451812805159
C	4.05224305840807	3.97843602868093	3.24420566276069
H	4.16788939383908	3.71209371244282	4.31248924304740
H	4.02585565916553	3.02832227688061	2.66306788818223
H	3.06167552179899	4.46213974231495	3.13354327816795
C	8.00378698422957	4.89364226965264	7.81969695768017
N	8.62318320635218	6.07484016557829	7.69145545061717
C	10.03473257871828	6.26832317266449	7.45279596215008
H	10.33720940441616	7.21646656026794	7.94636019879622
H	10.63525886368830	5.46581355230836	7.93974792630945
C	10.37156986593190	6.35125392948134	5.96134013686333
H	9.73320652515281	7.11805519325966	5.47906265701276
H	11.43792718474999	6.61357727418102	5.79993214526908
H	10.17087700928091	5.38339804724274	5.45849175961953
N	6.67649932150072	5.07232845910513	7.81925541143435
C	5.71330544997894	3.99227197580057	7.87346873314913
H	6.06473321414925	3.12363919411525	7.26740565123722
H	5.60480101066069	3.61056872361631	8.91856580781785
C	4.35359982641147	4.44875927960366	7.35145022459598
H	4.45097386865797	4.85596608791697	6.32452131657691
H	3.62861683076658	3.60966808465974	7.35141688445727
H	3.94710554153065	5.26636255544341	7.97660644113732
C	7.42487873209241	8.25173421458260	9.31770993183514
C	6.13113802761400	7.70363851616221	9.53070080333429
C	5.24281199465704	8.20682849137537	8.51156344274851
C	6.01177942685107	9.13825486790441	7.67704942154992
C	7.38594748474037	9.12819059722978	8.15715722556803
C	8.62958326503190	7.96724508989098	10.16266054553540
H	8.58454523352629	8.53300482352722	11.11850393062623
H	8.70670515054774	6.89050440609677	10.41307886605661
H	9.56504943783612	8.26031772391724	9.65030339439996
C	5.77570839635683	6.70007199029201	10.58650523345048
H	6.62573543958106	6.02207813570835	10.79895158715526
H	5.49735397802967	7.19712527111205	11.54139073194477
H	4.91831426577509	6.07320470080996	10.27401435012599
C	3.75581121109342	8.02580305558186	8.45122945921573
H	3.43420082117904	7.11966764559170	9.00096655882896
H	3.22210424911725	8.89228961371252	8.90340756315622
H	3.40879143953096	7.92483952602882	7.40670604736636
C	5.43381775135924	10.03582661148991	6.62180159352871
H	6.20813558126116	10.34952053042081	5.89587467827377
H	4.65046539805192	9.50667672329078	6.04543944794242
H	4.98758860787702	10.95573266962053	7.06122245289712
C	8.49404605556282	10.03295389695624	7.69763384599106
H	8.37609833799787	10.29823224452427	6.62934809530652
H	8.52002023829793	10.98361177755742	8.27789337760192
H	9.48552582041928	9.55082068681473	7.80693385438701
C	8.66788186682561	3.54387351748988	7.88261602487556
H	8.27023382337283	2.95237374128959	8.73125601298542
H	8.45711228204408	2.96908164584115	6.95661391597449
H	9.76419877561652	3.62762809885577	7.99292419357550
C	3.88134976780666	10.46406848896875	3.06600040164303
H	4.27823688269678	11.05577811316597	2.21715365091662
H	2.78495023799279	10.38014945036397	2.95664028328273
H	4.09275344328859	11.03879871854385	3.99188195986803

96

3_dia

Mo	5.82511674272242	7.16884032777466	3.77736809597929
Mo	6.48818781636223	6.92646938871849	6.30360497499719
N	7.38497822440011	7.63094471568198	4.78555247147687
N	4.92834608553445	6.46438468144879	5.29544832037134
C	4.55722431956679	9.36995014115788	2.91144765610275
N	3.96897533883907	8.23770129624734	3.29222526935125
C	2.54634900618803	7.98656561364463	3.35704995773855
H	2.41639009651832	6.88708434996427	3.41534026321050

H	2.04463912523578	8.30916370633691	2.41432458731386
C	1.84271389994407	8.62717208421286	4.55889954282712
H	2.28838655798070	8.25988787907326	5.50114046900508
H	0.76257663348526	8.37115202909487	4.56065757017585
H	1.93006414751503	9.73230756091801	4.54444626476807
N	5.88625748018329	9.22003070212381	2.92776116406455
C	6.81455783723868	10.33228476036654	2.90403150830583
H	6.82551777597575	10.84472629335270	3.89897891497090
H	6.47760689895641	11.10381006912951	2.17260151816788
C	8.23775398806979	9.88900625104265	2.58225196436687
H	8.53729185297174	9.09640067171350	3.29571202727510
H	8.94028891197519	10.74258811835928	2.66857925582005
H	8.31126205685847	9.48207851542148	1.55578042754283
C	5.03872812568892	6.10972753285803	1.60214642079633
C	6.31414638946790	6.71051937794171	1.31480162667111
C	7.29748194134200	6.12500812767930	2.16996481219694
C	6.60820231615983	5.16798997476458	3.01569438244894
C	5.20722597770509	5.14150475963962	2.64008554512627
C	3.79397476320742	6.39825382971825	0.82186673724075
H	3.85226578368298	5.92624782643568	-0.18360270672241
H	3.65431405132384	7.48577639282099	0.66483588889262
H	2.88914210303129	6.00600865886251	1.32085064667664
C	6.52859480401163	7.71060003687803	0.22077933988730
H	5.91128469162306	8.61880148419944	0.37527042888511
H	6.24984060404853	7.27815743079068	-0.76419579422301
H	7.58514519062368	8.03164290991371	0.16108240876699
C	8.76827727467990	6.39139628120146	2.20424062241934
H	9.08080118645463	7.06601888349125	1.38524725653495
H	9.35614354586247	5.45353473898598	2.11851321232070
H	9.03654979242590	6.87970716881929	3.16887536096820
C	7.28431251971075	4.16943611081591	3.90642346293394
H	6.58274940312922	3.76706168777356	4.65844361648893
H	8.13443663395120	4.61994556548982	4.44682797255821
H	7.66826041076116	3.31635368863811	3.30411937926657
C	4.17067863003330	4.25038071163882	3.24950553527686
H	4.08327540968288	4.47417149066008	4.33762238689232
H	4.42607577895358	3.17601782569263	3.13262413460893
H	3.17624824882804	4.41222789664710	2.78988434556763
C	7.75653223952091	4.72572501336501	7.16973932849294
N	8.34455494079635	5.85807079770220	6.78893763944502
C	9.76712162079793	6.10950538718163	6.72392551611316
H	9.89680653094467	7.20902176304878	6.66558652795352
H	10.26908643123388	5.78701630813536	7.66654717901143
C	10.47055730910381	5.46899477767050	5.52191845476436
H	10.02471324350832	5.83627623907856	4.57974613760714
H	11.55069216513555	5.72502923382046	5.51992058902256
H	10.38325169857898	4.36386437600705	5.53637045662319
N	6.42745156671590	4.87528380459434	7.15321440467258
C	5.49959371919958	3.76262905031743	7.17695874729777
H	5.48998397240223	3.24929327511196	6.18247569537218
H	5.83610598331127	2.99189800393589	7.90944762766964
C	4.07588241902482	4.20551614991244	7.49697279640272
H	3.77662426959812	4.99743075032582	6.78263593619502
H	3.37388703060794	3.35147991378939	7.41068592733130
H	4.00115540683502	4.61319762811769	8.52305476478737
C	7.27402364694251	7.98533516765900	8.47906738200666
C	5.99858229221086	7.38445879089573	8.76612793199967
C	5.01538470311537	7.97002216274406	7.91086043045424
C	5.70477214343492	8.92715607784246	7.06535893790097
C	7.10566860965434	8.95368809215981	7.44121088249527
C	8.51869826692720	7.69669834902636	9.25943581136588
H	8.46040321786262	8.16865594248846	10.26492694778521
H	8.65825871623833	6.60915441041073	9.41641838758099
H	9.42359314569174	8.08885356058589	8.76048969013882
C	5.78398772166062	6.38421185231359	9.85996897434452
H	6.40185445227082	5.47633811681389	9.70580803235657
H	6.06195926095751	6.81673990782744	10.84513242688268
H	4.72756251987239	6.06265309871920	9.91905046635928
C	3.54465065939920	7.70335724023621	7.87602516558067
H	3.23201591592980	7.02839216183307	8.69469311704854
H	2.95656665089244	8.64106747125799	7.96189873816600
H	3.27685887239124	7.21531073730612	6.91111195705740
C	5.02872390138011	9.92573200523025	6.17461987386464
H	5.73018563683971	10.32765071037566	5.42227337279430
H	4.17831421781399	9.47535733585769	5.63455113258851
H	4.64524018903943	10.77909431626429	6.77681429070578
C	8.14222923832820	9.84532560486656	6.83257727327314
H	8.23036214995655	9.62248743411295	5.74433065389012
H	7.88649044229454	10.91955509199465	6.95001606538032
H	9.13649280196091	9.68351505371472	7.29257645528674
C	8.47132249507045	3.45238351631216	7.53053620730228
H	7.96094346414403	2.93199351045738	8.36442099720647
H	8.47705353128933	2.75819962671405	6.66386725100689
H	9.52109753920345	3.64624789607334	7.81943391896446
C	3.84257237880291	10.64345411811274	2.55100189442550
H	4.35390633135496	11.16498813571443	1.71843439893630
H	2.79324409019087	10.44944196037955	2.26056505236141
H	3.83530574354655	11.33660702779908	3.41849486404393

86

4_lin

Mo	4.35090511927871	5.25669029219319	5.07397005958904
Mo	5.07114999767594	0.61944910557147	6.20963438827643
N	4.68216060259298	3.49295265772107	5.34341045579293
N	4.93536116236031	2.39672240776039	5.87011035128042
C	5.05241399794981	5.22017512812893	2.97041040602067
C	5.56105328324502	4.02733922086309	2.24755673857186
C	4.95541110076403	3.60081870197643	1.04476297523573
H	4.11147577066433	4.17269154542597	0.62877226034284
C	5.43092663987177	2.46439561713468	0.37168142628918
H	4.95398376314359	2.14729902549053	-0.56840815240527
C	6.51070898784383	1.73115734364210	0.89534458388683
H	6.87786060956747	0.83590962329331	0.37097338353492
C	7.11543183695730	2.14517168694807	2.09748065524716
H	7.94820794915830	1.56581456403954	2.52447938780801
C	6.64938474852583	3.28607964940710	2.76320363856335
H	7.09856555468911	3.58720377199668	3.72138048988772
N	3.72138377898044	5.56891254714339	3.06072682990270
H	3.09613993435560	4.89825712266216	2.60634328888423
N	5.82631862536292	6.00549830955297	3.79375748559296
H	6.81345267476820	5.74491966594220	3.82887218032319

```
C  2.61045924126370   6.55475552842330   5.96430904997143
C  3.80929558072336   7.35767169100078   5.88860630889506
C  4.81637165907923   6.76128250724787   6.74721924478653
C  4.21145116870366   5.61238417378426   7.37758530185554
C  2.85911770345645   5.48738167384343   6.89609544235485
C  1.32547390037778   6.83987794065020   5.24659028703687
H  0.68279387952592   5.93992465286699   5.19499510050873
H  1.51735218962315   7.17933313106040   4.20942161792475
H  0.74806138594342   7.63898322809613   5.76062090893043
C  3.98192785041165   8.59636558391188   5.05905150997658
H  3.39385790578364   8.53872571527565   4.12281447768444
H  5.04119330310390   8.74447723002681   4.77603702525681
H  3.64721963613626   9.49971093375642   5.61524752075201
C  6.17829395708486   7.32126980690261   7.03011141927006
H  6.13345173845081   8.12132880074005   7.80171710727962
H  6.62569766555775   7.76231681681741   6.11774182650678
H  6.86291892889385   6.53372013456251   7.39839761410404
C  4.86870449520005   4.69841042145457   8.36918651015369
H  5.96991942227719   4.73577727069211   8.28468728968317
H  4.56648861686511   3.64395067176193   8.21467886636351
H  4.59770183137696   4.98078578294726   9.40937607805648
C  1.88985970447954   4.42574638956485   7.32226937738815
H  1.37761539286844   4.71499462025168   8.26603158335054
H  2.40388006395913   3.46245009148133   7.50557944428352
H  1.10871139695561   4.25438722403773   6.55694782660902
C  6.85798555847910   0.85311074797430   7.50322599226121
C  7.52542091342977   2.13696132661444   7.83567356473191
C  7.98092153042327   2.99468938504754   6.80770673320244
H  7.80866093861528   2.71332008221091   5.75811862051847
C  8.58896878993239   4.21819692045162   7.11615192516735
H  8.91605908901837   4.88533457935803   6.30409538627379
C  8.76594322566890   4.60137781211503   8.45937393290086
H  9.24416433876607   5.56257857315983   8.70169400296285
C  8.32085993578115   3.75303262243339   9.48891065854357
H  8.45697813443992   4.04550453787749   10.54139244394923
C  7.70013978181251   2.53223565633268   9.18042133659896
H  7.35604227472239   1.87049269063031   9.99038117173835
N  7.10172182812036   0.14017109590850   6.35191381131483
H  7.83473347761657   0.52841977548817   5.75567674091407
N  5.78966809056775   0.32508219955590   8.19690904936283
H  5.46280808491132   0.92868878317792   8.95590269100664
C  4.45696376810490   -1.57339024915382   5.77460616675660
C  3.34235015957992   -0.95095194087568   6.45076852695410
C  2.84561387905007   0.09661069850818   5.59920100151878
C  3.66198497189034   0.14059629105365   4.41250310145637
C  4.67556306488432   -0.88184310365527   4.51715091076779
C  5.25350845468795   -2.73729138093547   6.28747246210396
H  6.29248649159363   -2.71049996687297   5.90807463856428
H  5.30475041154178   -2.73414927265569   7.39333831744539
H  4.79924416055658   -3.70238651494122   5.97176041820316
C  2.77722945364039   -1.37871126965873   7.77184808454655
H  3.58591811605739   -1.63131088830426   8.48600337870535
H  2.16171908277892   -0.57871697893893   8.22649229162779
H  2.13389160231974   -2.27858410428220   7.66021766039332
C  1.67295106393852   0.99073080530408   5.87018576299208
H  0.75165877837052   0.58876851645933   5.39438979385621
H  1.47454035086619   1.08666186980570   6.95495527912114
H  1.83998144811374   2.00691679990013   5.46384259567887
C  3.48099996841320   1.09147068393225   3.26635217616179
H  3.16952808463250   2.09573791516708   3.61468551972842
H  4.42045019311798   1.22232850518252   2.69913794537877
H  2.70636290153111   0.72065270675187   2.56094293436694
C  5.67600829806075   -1.25917691987311   3.46577306903446
H  5.30307002429254   -2.09682928469517   2.83587807633841
H  5.89260973723537   -0.40388795304177   2.79778758074015
H  6.63131223802549   -1.58487293729994   3.92239854324448
```

86

4_int-1

```
Mo  5.08582563862086   4.40197879234351   4.24776554442001
Mo  5.62103078867159   1.59262164615803   6.35825284469187
N  6.13390182858112   2.98101281073331   4.77940958134901
N  7.08828877384588   2.27852447463577   5.29407190946000
C  3.51623341183818   4.38821974767390   2.21451404270542
C  2.67301702384878   4.96153968310526   1.14997976771727
C  1.27280043076831   5.09610173303315   1.32764817818922
H  0.80563243465841   4.76350676567136   2.26688557189558
C  0.46912157988209   5.64125218581363   0.31798566222361
H  -0.61557071875647   5.73879940935156   0.47885878566504
C  1.04222541923414   6.05988806702657   -0.89751174609405
H  0.41075491356358   6.48885948460522   -1.69019638407413
C  2.42953892350793   5.92372410396206   -1.09125555157506
H  2.88823750033801   6.24730630480118   -2.03825774524309
C  3.23555193182612   5.38351772028110   -0.08084149336021
H  4.31954399340743   5.29578949334770   -0.24710132802736
N  3.10634406514087   4.05460149662727   3.45951162338953
H  2.17306828170122   4.35995138954309   3.73080344950098
N  4.84960456998338   4.19678656703301   2.16334141323247
H  5.35606464793577   4.47072023323836   1.32534226610063
C  4.21991674005891   6.60515781256052   4.77947465868303
C  5.21645450569137   6.73951828728304   3.77193839221831
C  6.46710755912015   6.17422886399392   4.25879086450504
C  6.21381626962195   5.73554575485553   5.62407266799027
C  4.80505429002145   5.95227793118948   5.92668763875900
C  2.78774695967185   7.03464477975825   4.66909663408551
H  2.12032331672384   6.36412087639126   5.24600238312264
H  2.44015572290509   7.03990738387217   3.61742563223761
H  2.64856947644483   8.06212697148630   5.07022014971837
C  5.03645092562807   7.38505691080833   2.43106435582097
H  3.98325797051476   7.35892005704139   2.09169520534730
H  5.65192272443282   6.89016187137940   1.65448404354345
H  5.35074675930427   8.45089960874858   2.46635484502705
C  7.80794473688520   6.31338283382758   3.59827119142925
H  8.27454234412736   7.30213270068934   3.81390185274300
H  7.72627937178774   6.21892635496441   2.49711653788020
H  8.50841800175010   5.53008824907759   3.94689130694398
C  7.26076382518630   5.27182522560919   6.59441031401616
H  8.10608080457872   4.78168264337194   6.07559197699214
H  6.85184148894682   4.52815998528016   7.30316114513112
H  7.66535827167104   6.12658479125070   7.17900590418776
C  4.13823300437243   5.74625580714553   7.25495124077874
```

H	4.22899100316586	6.64577528310058	7.90585914973717
H	4.59229498309908	4.89404114449388	7.79332622466918
H	3.05887964079560	5.52666093666891	7.13631374592437
C	6.93573327302307	1.94996818714179	8.57296211262516
C	7.77849840566996	2.34802419447344	9.72277339285422
C	8.65267786908920	1.41396023072921	10.32530894912236
H	8.69626678095881	0.38433418856983	9.93867893536395
C	9.44124953885420	1.78521713499684	11.42295361964229
H	10.11410965010786	1.04807944109287	11.88709262088104
C	9.36866331757088	3.09407528057834	11.93507734076010
H	9.99000355767763	3.38506277249033	12.79577312304414
C	8.50191586365094	4.02961602608621	11.34267749021540
H	8.44792961569180	5.05706181049498	11.73372284894892
C	7.71211049162758	3.66081586293450	10.24386189645217
H	7.05667459069271	4.40389470642879	9.76576230064321
N	7.18262539253668	0.94934620339577	7.72776234297296
H	8.11347197418480	0.53778336491763	7.70959376650008
N	5.80894304051885	2.58616787312888	8.19646447968249
H	5.35641089225755	3.24001728079200	8.82922164592849
C	4.59601964951889	-0.34176664095531	7.30882359892061
C	3.62602367435867	0.68362936288360	7.50152967360324
C	3.31284400438683	1.27669533798861	6.23368990221862
C	4.06915977286432	0.55278397500662	5.20738595438216
C	4.89476076731017	-0.44284030766415	5.88649661990379
C	5.16806145027213	-1.20994450505147	8.38745595861372
H	6.12871962849934	-1.66031046617320	8.07765649926733
H	5.35213700515119	-0.63642698358591	9.31697388358941
H	4.46707537610222	-2.03712457991515	8.63464264553627
C	3.06058488532168	1.11783521780103	8.81787944517593
H	3.77650151302937	0.94765290767566	9.64527810712097
H	2.79350760959806	2.19251687010469	8.80716897758561
H	2.13470300704636	0.54990546687112	9.05499399698192
C	2.20453029179988	2.26004203226468	6.00460945584865
H	1.21236075056140	1.76241916791507	6.08997731739135
H	2.23105804024111	3.08714440310615	6.74131558890688
H	2.28700087196298	2.70240587430622	4.99825618918248
C	3.91023079499605	0.71715347941014	3.72413258583738
H	3.74335357946608	1.78048455059728	3.45801298079621
H	4.81001661559858	0.36952234192451	3.18078389804876
H	3.04165686895664	0.13187454422881	3.34973736715721
C	5.72055877931050	-1.51662099486803	5.23743099936616
H	5.12964652592933	-2.44837191825597	5.08741511016564
H	6.09119512998952	-1.19397275148774	4.24544970143530
H	6.60396175801236	-1.77659827415975	5.85348896116904

86

4_int-2

Mo	5.81546989985249	6.87617693350643	3.67836532255643
Mo	6.73029671061104	7.13258961658957	7.27003296613765
N	6.96700564647713	6.94282236122218	5.39032497665365
N	5.57970967848091	7.06478309136744	5.55832329044681
C	4.60653606247224	9.11126581961448	3.06685036561982
C	3.91698105807956	10.41426257125974	2.93013429994936
C	4.59228001468419	11.62272843437217	3.22107186104441
H	5.63183027097486	11.59217165045083	3.57994947859479
C	3.93796596987170	12.85567642168881	3.08759879918971
H	4.47461725065297	13.78693228153062	3.32534920386174
C	2.59739360198820	12.90307300455663	2.66434036191434
H	2.08310565306782	13.87100455476457	2.56363222070155
C	1.91605607365854	11.70689507977256	2.37212540180510
H	0.86883504158909	11.73721845369557	2.03411397774339
C	2.56939821304873	10.47367540586920	2.50330788168949
H	2.03705239449602	9.54301670298301	2.25404210335735
N	3.99857738744615	7.92269231318963	3.20623322351197
H	2.99807838015313	7.89633407788403	3.38341216907118
N	5.93290312112226	8.93525046988426	3.11243126739434
H	6.55626131957449	9.71643759238400	2.93152562997395
C	5.32728576433775	5.74360746958601	1.61505001235513
C	6.63960049422946	6.27777559018612	1.46956183296895
C	7.46019900901667	5.77899365345000	2.54530385907268
C	6.64031189718528	4.85676260045827	3.33733996904153
C	5.29889204760730	4.86848456958012	2.77868342697335
C	4.16012461672935	6.02592388981980	0.71804513449162
H	4.13639297078714	5.31523107262394	-0.13712096100626
H	4.20401642512019	7.05152059506153	0.30281033404350
H	3.20159364077013	5.92792960734239	1.26199356443207
C	7.07565923029252	7.26021965618752	0.42460595121185
H	6.23938299541924	7.91365377390361	0.10798125300130
H	7.45479552928885	6.73786681753445	-0.48057800963536
H	7.88980338043436	7.91131750198887	0.79801868209415
C	8.92722231746522	6.02055324890905	2.74488545649088
H	9.23579752967109	7.00218642148986	2.33429677317190
H	9.54342718898866	5.23903132586693	2.24615003412638
H	9.18328353317646	6.01357213695549	3.82269866516317
C	7.13360624955752	3.97131638182794	4.44527366906716
H	6.34730522321205	3.80761331998616	5.20694866722951
H	7.99023964186538	4.43228769957944	4.97195345521353
H	7.45539013964343	2.97778275227802	4.06236951438573
C	4.16031449736460	3.97319534442122	3.17679752291000
H	4.23295006811100	3.68495509774782	4.24332505495275
H	4.14692993901951	3.03541052533727	2.57582198108709
H	3.18113291164850	4.47158065094612	3.03262243762499
C	7.94074846636132	4.89872416718354	7.88095018518864
C	8.63123705850492	3.59575790072731	8.01417044819832
C	7.95660290744005	2.38752619745216	7.72080808679043
H	6.91681614295663	2.41823613587759	7.36264956493391
C	8.61193574170204	1.15475592529195	7.85093824433899
H	8.07585609773187	0.22366261721655	7.61126637721544
C	9.95278457029136	1.10736083251702	8.27329101007523
H	10.46785568773168	0.13957547951519	8.37137443853435
C	10.63340960184765	2.30332169525971	8.56800270343174
H	11.68083202496024	2.27294687876237	8.90538492648798
C	9.97908810917055	3.53637664420240	8.44012095783114
H	10.51081837941160	4.46686357415706	8.69131858616493
N	8.54818304754413	6.08780308972723	7.74341227512215
H	9.54835513056796	6.11434219316897	7.56440351108237
N	6.61425490915676	5.07398802911181	7.83671652702728
H	5.99149640807526	4.29174993072643	8.01499620887581
C	7.21727645910243	8.26856689233654	9.33236081654820
C	5.90696345009106	7.72991942895970	9.47905409914039
C	5.08380911135652	8.22537473099584	8.40352117611301

C	5.90012709968888	9.15010026817719	7.61060575386385
C	7.24181228960911	9.14314860810415	8.16842144569128
C	8.38625652761576	7.99041984353266	10.22828556536385
H	8.40970670727754	8.70294376438868	11.08192531317538
H	8.34489946017823	6.96558241777555	10.64560095074035
H	9.34384756821406	8.08926562648293	9.68281649563646
C	5.47489908055760	6.74658225848801	10.52484711423352
H	6.31367112090018	6.09640780860281	10.84156981594069
H	5.09415349228527	7.26817740768621	11.42979256738860
H	4.66306603138681	6.09224069286071	10.15210369264178
C	3.61739100066361	7.97876160179107	8.20563803735696
H	3.31316309540174	6.99536469615662	8.61527538751756
H	2.99894498438946	8.75717719530058	8.70648070975908
H	3.35967948253890	7.98626733125609	7.12822507141787
C	5.40357758597769	10.03342682163855	6.50241506232965
H	6.18672950572255	10.19357920892686	5.73673311435782
H	4.54385997768654	9.57255520387727	5.98074330716674
H	5.08528881361832	11.02853837901700	6.88412046850463
C	8.37695742936363	10.04227131036402	7.76920586794653
H	8.30336812327750	10.32893875749295	6.70231596689449
H	8.38651316886559	10.98088272735690	8.36895334768380
H	9.35806346701579	9.54792780198812	7.91418951771514

86

4_dia

Mo	5.86663843796450	7.07457063949485	3.57824061311848
Mo	6.51624116423536	7.04584114322247	6.12883247956364
N	7.37369685929118	7.79651722200226	4.73354960737152
N	4.86319973506465	6.83116023899515	5.11853787648606
C	4.44018456189751	9.11089440529711	2.77963839462281
C	3.64301727290264	10.36467016480535	2.78057562272389
C	4.20786086004592	11.57775267066397	3.23270194223961
H	5.24959543481883	11.59691471253726	3.58623535718217
C	3.44313871824056	12.75316921567400	3.25830630831401
H	3.89205807096761	13.68983355290512	3.62211698883998
C	2.10474807707205	12.73204529272360	2.82764693598359
H	1.50431695152307	13.65436157848383	2.84791194083289
C	1.53559536694689	11.53023546130291	2.36962874933635
H	0.49071472716087	11.51114079166438	2.02428367318660
C	2.29930419583306	10.35412061506256	2.34638037277424
H	1.85846742375654	9.41724232226228	1.97327186073243
N	3.93374850086792	7.87741839245780	2.78692766826393
H	2.97235930055198	7.78032247152721	3.11526599991180
N	5.77791263107519	9.05953071105269	2.83739381390183
H	6.33361972344400	9.90960115263148	2.88327419451552
C	5.87422607566243	6.25441734287317	1.31657994668785
C	7.24997580349981	6.61968963400567	1.50060260398349
C	7.78343175629559	5.84434409205579	2.56964148116564
C	6.73263802629853	4.97639811265445	3.06567356289217
C	5.54293163607019	5.22318416761674	2.28487619845466
C	4.98708689837981	6.77871720125447	0.22868795366956
H	5.29362398884909	6.36544028279548	-0.75748710755536
H	5.03926539485703	7.88366680925805	0.15722396270226
H	3.93056061856631	6.50784424897525	0.40240247246295
C	7.96925215351086	7.69317463446311	0.74099125295084
H	7.27604904276281	8.51014212551910	0.45899133177645
H	8.41515290580076	7.29686119966627	-0.19718379772502
H	8.78700257268461	8.13625705689899	1.34143677208406
C	9.16593577171095	5.94307453621087	3.13056004748931
H	9.89502742183197	6.27036098905825	2.36435706266476
H	9.50742803516441	4.97338703136494	3.54288236484948
H	9.17195701581447	6.68841680887205	3.96135662733195
C	6.91025126444710	3.82558750244170	4.01058084796451
H	6.04699403280450	3.71684443859387	4.69191016271073
H	7.80658865547132	3.95817917527715	4.64134940280851
H	7.02550589387729	2.87842694137098	3.43909053183473
C	4.26517940390207	4.43973702285104	2.35345159839182
H	4.08167324381645	4.06753378499050	3.37990683662545
H	4.28899229953762	3.55931792286985	1.67295935380752
H	3.39692444845337	5.06343251572635	2.06374128246668
C	7.31554661346557	4.63796001208490	7.12944887989135
C	7.90501600880518	3.39862632466085	7.69420848967165
C	7.17035037122736	2.19119771922030	7.68565413405870
H	6.16153144916040	2.17598855987027	7.24563634775947
C	7.72703882630480	1.01490572681140	8.20802300326072
H	7.14697936809275	0.07955901483384	8.18861012089275
C	9.02582757377885	1.02749298758503	8.74853745711359
H	9.46161061954562	0.10417164986059	9.15994016201443
C	9.76586980820614	2.22331829806252	8.76055042712535
H	10.78054974097673	2.23983449994613	9.18703400978390
C	9.21108810780509	3.39968577627330	8.23493597268291
H	9.78642743456286	4.33775005702082	8.26041224923668
N	8.04639941407301	5.65551858089264	6.64099800595818
H	9.04733408180434	5.56182655816173	6.50053530588036
N	6.02687139816693	4.91145322121633	7.01840215249087
H	5.36023701457402	4.28531251853096	7.46643018159211
C	7.47536136912235	8.53875936738742	7.80442653762664
C	6.60989653308543	7.62236348389428	8.51547540933311
C	5.26341533648194	7.86509127956563	8.09221054224003
C	5.28974600587411	8.92139390282989	7.11844811850122
C	6.65594655578776	9.36504954551438	6.97330342134175
C	8.96371375418021	8.64596921483854	7.95757970777191
H	9.24481451245593	9.38442075681115	8.74054072705363
H	9.40466600463553	7.67199584062538	8.24804835086153
H	9.44186159471342	8.96339103000872	7.01004226946058
C	7.05683202370100	6.66903260655940	9.58100592673837
H	8.00295959302309	6.16560186512932	9.30065453311602
H	7.23913315548663	7.20464550253747	10.53799112020543
H	6.30214634792308	5.88312172377599	9.76995069592887
C	4.02231693056632	7.15010202784209	8.53259335873938
H	4.26267283191964	6.24498659317042	9.12218067768267
H	3.37986192230027	7.80364795586078	9.16192975655550
H	3.42184239962232	6.83668166798906	7.65430380348238
C	4.07734742790724	9.48128505905356	6.44143135834840
H	4.33792072942591	10.29843521952444	5.74328010220604
H	3.57480815244831	8.68478958042558	5.84993206309436
H	3.34856235219618	9.87669764895490	7.17947674050205
C	7.15277274576611	10.44954344526666	6.07494758910930
H	7.61094592803623	9.98306841396498	5.16850877390582
H	6.32872749162505	11.11096054762080	5.74374323299980
H	7.91989956545755	11.07473660398910	6.57344524379784
